# 3^rd^ Guideline for Perioperative Cardiovascular Evaluation of the Brazilian Society of Cardiology

**DOI:** 10.5935/abc.20170140

**Published:** 2017

**Authors:** Danielle Menosi Gualandro, Pai Ching Yu, Bruno Caramelli, André Coelho Marques, Daniela Calderaro, Luciana Savoy Fornari, Claudio Pinho, Alina Coutinho Rodrigues Feitosa, Carisi Anne Polanczyk, Carlos Eduardo Rochitte, Carlos Jardim, Carolina L. Z. Vieira, Debora Y. M. Nakamura, Denise Iezzi, Dirk Schreen, Eduardo Leal Adam, Elbio Antonio D’Amico, Emerson Q. de Lima, Emmanuel de Almeida Burdmann, Enrique Indalecio Pachón Mateo, Fabiana Goulart Marcondes Braga, Fabio S. Machado, Flavio J. de Paula, Gabriel Assis Lopes do Carmo, Gilson Soares Feitosa-Filho, Gustavo Faibischew Prado, Heno Ferreira Lopes, João R. C. Fernandes, José J. G. de Lima, Luciana Sacilotto, Luciano Ferreira Drager, Luciano Janussi Vacanti, Luis Eduardo Paim Rohde, Luis F. L. Prada, Luis Henrique Wolff Gowdak, Marcelo Luiz Campos Vieira, Maristela Camargo Monachini, Milena Frota Macatrão-Costa, Milena Ribeiro Paixão, Mucio Tavares de Oliveira Junior, Patricia Cury, Paula R. Villaça, Pedro Silvio Farsky, Rinaldo F. Siciliano, Roberto Henrique Heinisch, Rogerio Souza, Sandra F.M. Gualandro, Tarso Augusto Duenhas Accorsi, Wilson Mathias Júnior

## 1. Definition of the Problem

### A) Purpose of the Guideline

The main aim of the guidelines is to update the concepts promulgated by its two predecessors, namely, the I and II Guidelines for Perioperative Evaluation of the Brazilian Society of Cardiology published in 2007 and 2011, respectively.^1^ When the systematic review of the collected evidence was conducted after five years since the last publication, we noticed a remarkable evolution of the knowledge on the subject, particularly in cardiology. In the perioperative environment, the physician needs to simultaneously gather concepts from different specialties to understand different aspects of the same problem and to optimize the language among clinicians, surgeons, anesthesiologists, and intensivists. Although problems related to other disciplines are addressed in this III Guidelines, we decided that the text should adopt the point of view of a cardiologist. In line with this decision, the III Guidelines incorporated the term cardiovascular and was thus termed Guidelines for Perioperative Cardiovascular Evaluation. Based on new findings, some novelties were included, such as new oral anticoagulants and surgical interventions in patients with dual antiplatelet therapy (DAPT), in patients with last generation stents. Anticoagulants and antiplatelet agents were discussed in more detail for specific surgical procedures, such as dental, dermatological, endoscopic, and ophthalmologic.

### B) Methodology and Evidence

The systematic review performed to elaborate the III Guidelines considered aspects related to the serious allegations of fraud involving the work of the group led by Don Poldermans of the Erasmus Medical Center in the Netherlands. The group published studies, known as DECREASE trials, which investigated important aspects in the perioperative environment, such as the use of β-blockers and biomarkers and invasive stratification, in significant groups of patients. The report released by the Erasmus Medical Center describes several problems in these studies, including neglect and scientific inaccuracies, especially in DECREASE IV.^2^ Other studies from the same group, such as DECREASE V and VI, also presented similar problems, although to a lesser extent.^3,4^ The conclusions of the report led to the dismissal of Don Poldermans from the Erasmus Medical Center and to the notification of the journals where these papers were published. However, as of the date of the publication of the III Guidelines, the published studies are still available on the sites of the journals and have not been withdrawn. The members of the writing committee of the III Guidelines discussed the matter and unanimously decided that the recommendations should NOT consider the findings of DECREASE IV, V, and VI and that the readers would be informed of this decision.

The methodology and levels of evidence considered for III Guidelines are as follows:

## 2. Preoperative Evaluation

### A) History

Collection of clinical history is the first approach in perioperative evaluation. Anamnesis performed with the patient or family members may provide information on the clinical conditions that determine the estimated surgical risk. The algorithms for perioperative risk assessment use the data obtained by clinical history and physical examination. The study of medical records in medical charts and anesthetic records is useful for retrieving previous information.^5,6^

To guide the evaluation of surgical risk, the following data are obtained in clinical history: Information of the underlying disease, which indicates the surgical procedure, including information from the surgeon on the risk and location of the procedure, the availability of technical support regarding personnel and equipment, the type of anesthesia, the estimated surgical time, and the need for transfusion; clinical, sociodemographic, and cultural data, such as age, gender, blood type, positive serology for hepatitis C virus, and acceptance of transfusion; data to assess the patient’s psychological/psychiatric condition; thorough investigation of surgical or anesthetic history that may reveal potentially preventable complications or allergies; determination of functional capacity, investigating daily activities (Chart 1).

**Chart 1:** Questionnaire to evaluate the functional capacity

Can you...	METS*
Take care of yourself: dressing, eating, and bathing?	2.75
Walk a block or two, with no hills?	2.75
Climb stairs or go up a hill?	5.50
Run a short distance?	8.00
Do light work at home, such as picking up trash or washing dishes?	2.70
Do moderate work at home, such as vacuuming, sweeping floors, or storing/carrying groceries?	3.50
Do heavy work at home, such as scrubbing/washing floors or lifting or moving heavy furniture?	8.00
Do garden/yard work, such as using a scrub, gathering leaves, or using a lawn mower?	4.50
Have sexual activity?	5.25
Participate in moderate recreational activities, such as bowling, dancing, or playing tennis in doubles?	6.00
Participate in sports activities, such as swimming, individual tennis, or football?	7.50

Adapted from Hlatky et al.^7^

* MET < 4 is considered low functional capacity. MET: metabolic equivalent.

Investigation of the clinical condition and the need to compensate for coexisting diseases, with a focus on identifying the presence of serious cardiovascular conditions in the perioperative stage (Chart 2).

**Chart 2:** Severe cardiovascular conditions in the perioperative period

Acute coronary syndrome
Unstable diseases of the thoracic aorta
Acute pulmonary edema
Cardiogenic shock
Heart failure NYHA functional class III/IV*
Angina CCS functional class III/IV*
Severe bradyarrhythmias or tachyarrhythmias (third degree AV block, VT)
Uncontrolled systemic hypertension (BP > 180 × 110 mmHg)
Atrial fibrillation with high ventricular rate (HR > 120 bpm)
Symptomatic pulmonary arterial hypertension

* Patients with these conditions who are stable and whose treatment was already optimized should have the risk-benefit ratio of the surgical intervention analyzed due to the risk of complications. NYHA: New York Heart Association; CCS: Canadian Cardiovascular Society; BP: blood pressure; HR: heart rate; AV: atrioventricular; VT: ventricular tachycardia

In patients more than 65 years old, verification of the degree of fragility;^8-18^ identification of the presence of valvular heart disease (item 4.D), valvular prostheses, and the need for prophylaxis for bacterial endocarditis (item 7.E); investigation of risk factors for cardiopathies; record of the presence of pacemaker or cardioverter/implantable defibrillator and adequate management (item 4.G); diagnosis of peripheral vascular disease, renal insufficiency, cerebral vascular disease, diabetes mellitus (DM), liver disease, hemorrhagic disorders, thyroid disorders, obstructive sleep apnea, and chronic lung disease; use of drugs, phytotherapics, alcohol, illicit drugs, and evaluation of potential interference with the operative procedure.

Doubts of the patient and their relatives regarding the procedure and its risks. Awareness and agreement on the risks and benefits of the procedures. Awareness that surgical risk is not limited to the intraoperative period and, eventually, a prolonged follow-up will be needed. Awareness that complications are not limited to the cardiovascular system;

The data obtained in the clinical evaluation should be dated and recorded in appropriate documents. The day and time of receiving the request and writing the evaluation response should be recorded. Establish a system that expedites preoperative consultation requests in the institution. The information must be available in a legible and explicit format, and the most relevant should be underlined. The preoperative consultation may not be finalized in the first evaluation; ensure that the preoperative consultation has been forwarded and, if necessary, contact the surgeon or anesthesiologist in person or by other means of communication;

Consider the patient’s expectations regarding return to appointments, performance of tests, scheduled date for surgery, waiting list for the surgical procedure, precocity of the procedure, availability of appointments, and operating room.

### B) Physical Examination

Physical examination is useful during the perioperative risk assessment process and should not be limited to the cardiovascular system. This examination aims to identify pre-existing or potential cardiopathy (risk factors), define the severity and stability of the heart disease, and identify possible comorbidities.

Patients with heart disease whose general condition is compromised by other conditions, such as neurological diseases, renal failure, infections, liver abnormalities, malnutrition, and pulmonary dysfunction, are at a higher risk of cardiac complications because such conditions exacerbate surgical stress. Patients with peripheral vascular disease have a high incidence of ischemic heart disease, which is a prognostic factor of perioperative complication.

Evidence of, for example, changes in arterial pulses or carotid bruit should be investigated in physical examination. On the other hand, turgid jugulars in preoperative consultation, indicating high central venous pressure (CVP), suggest that the patient may develop postoperative pulmonary edema.^19^ The evidence of a third heart sound (S3) in the preoperative evaluation is indicative of poor prognosis with an increased risk of pulmonary edema, myocardial infarction, or cardiac death. The evidence of lower limb edema (bilateral) should be analyzed in combination with the presence or absence of jugular venous distention. If CVP is increased, visualized by the height of the oscillation of the pulse of the internal jugular vein, then cardiopathy and pulmonary hypertension (PH) are responsible, at least partially, for the patient’s edema. If CVP is not increased, other causes, such as liver disease, nephrotic syndrome, chronic peripheral venous insufficiency, or use of some medication, are responsible for the edema. Evidence of edema alone and without knowledge of the patient’s CVP is not a definite sign of heart disease.^20^ In the presence of heart murmurs, the physician should be able to distinguish between organic and functional murmurs, determine if they are significant or not, and identify their origin. The origin will indicate whether endocarditis prophylaxis or evaluation of valvular lesion severity is necessary (items 4.D and 7.E).

In elderly patients, a brief assessment of fragility can be performed using the timed up and go test. In this test, time is measured in seconds with a timer starting from the point when the patient is given the command to get up from a chair and walk three meters forward and return, and ending when the patient sits back in the chair. A test time equal to or greater than 20 seconds is considered poor/low. A test time equal to or greater than 15 seconds is associated with postoperative complications and increased mortality in a period of one year.^15^

### C) Additional Tests

In the perioperative evaluation of patients scheduled for surgical procedures, requesting of preoperative tests [electrocardiogram (ECG), chest X-ray, and laboratory tests] is a common and routine clinical practice. However, this is not related to the reduction or prediction of perioperative complications and has a high economic cost for the health system. Therefore, revisions elaborated by several societies have recommended the rational use of tests.^21-23^

In the literature, few studies have evaluated the benefit and impact of preoperative routine tests. Cataract surgery is the surgical procedure that presents the best evidence. Three randomized studies compared performing and not performing routine preoperative examinations and the occurrence of postoperative events in patients undergoing cataract surgery.^24-26^ The systematic review of these three studies, involving 21,531 patients, showed a similar frequency of complications between the two groups. The authors concluded that performing preoperative tests does not increase the safety of cataract surgery and is associated with a 2.5-fold higher cost when compared to the group that did not perform preoperative tests.^27^ Despite the evidence in the literature, routinely requesting preoperative tests is still common in clinical practice. In a cohort study with 440,857 patients, the authors observed that more than half of the patients undergoing cataract surgery had a preoperative test, especially when the evaluation is performed by ophthalmologists.^28^

For other surgeries, only one randomized study investigated the effect of routine preoperative tests on the occurrence of postoperative events and complications.^29^ The population of this study mainly consisted of patients with low clinical risk, without serious diseases or decompensated clinical conditions, who underwent small and outpatient surgeries. In this study, the patients were randomized to perform the proposed surgery with or without preoperative tests (ECG, chest X-ray, blood count, urea, creatinine, electrolytes, and glucose). No difference in perioperative morbidity and mortality was found between patients who performed preoperative evaluation with complementary tests and those who did not perform additional tests. The American National Institute of Health conducted an observational study on 73,596 patients undergoing low-risk and selected outpatient procedures (hernia surgeries). The authors reported that 54% of those without comorbidities underwent preoperative tests. The frequency of perioperative complications was extremely low (0.3%). The performance of preoperative tests or the presence of abnormalities in these tests did not predict complications.^30^

An extensive review of the literature has shown very limited evidence of clinical effectiveness to recommend routine preoperative tests. No study has demonstrated the cost-effectiveness of preoperative tests in healthy individuals undergoing low-risk or intermediate non-cardiac surgeries.^31^ Abnormal findings in routine tests are relatively frequent, but these results rarely lead to changes in surgical procedure or surgery suspension. In addition, changes in preoperative tests do not predict complications.

In conclusion, there is no indication to perform routine laboratory tests in the preoperative evaluation in asymptomatic patients submitted to low-risk procedures. The indication of preoperative tests should be customized in accordance to the history and physical examination, the diseases and comorbidities presented by the patients, as well as the type and extent of the proposed surgery.

#### I. Electrocardiogram

The ECG analysis may complement cardiologic evaluation and allow the identification of patients at high cardiac risk. The ECG can detect arrhythmias, conduction disorders, previous myocardial ischemia or acute myocardial infarction (MI), ventricular overloads, and changes due to electrolyte disorders or drug effects. In addition, a baseline electrocardiographic tracing is important for perioperative comparative evaluation in patients at high risk for cardiovascular events.

However, routine application of a test with limited specificity may lead to false-positive results in asymptomatic patients, since electrocardiographic abnormalities often concern the surgical and anesthetic staff and may prompt the unnecessary cancelation of the surgery.^32^ Abnormalities found on the ECG tend to increase with age and the presence of comorbidities, and these electrocardiographic changes usually have a low prognostic value regarding complications.^33,34^ In a retrospective study involving more than 23,000 patients, the presence of preoperative electrocardiographic changes was associated with higher incidence of cardiac deaths within 30 days.^35^ This result was corroborated by two prospective studies that found similar results, where preoperative ECG abnormalities predicted perioperative cardiovascular events.^36,37^ However, in the group of patients submitted to low to moderate risk surgery, preoperative ECG presented limited prognostic information.

Therefore, the indication for preoperative ECG depends on clinical history, surgery type, and diseases presented by the patient.

#### II. Chest X-ray

Studies evaluating the routine use of chest radiography (X-ray) in the preoperative evaluation have shown that the result of the test rarely interferes with the management of the anesthetic technique and does not predict perioperative complications. Abnormalities found in the X-ray are usually related to chronic diseases, such as COPD and/or cardiomegaly, and are more frequent in male patients older than 60 years, with a higher cardiac risk and with more comorbidities.^40,41^ The indication for preoperative chest X-ray should be based on an initial careful evaluation by using the clinical history and physical exams of the patients. There is no indication for routine chest X-rays in asymptomatic patients as part of the preoperative evaluation.

### D) Perioperative Evaluation Algorithms

Over the years, several indices have been developed to estimate the risk of perioperative events in noncardiac surgeries. Based on these risk indexes, algorithms/flowcharts are suggested to facilitate the perioperative evaluation process and propose strategies to reduce the risk of the events.

#### I. Risk Indices

Several papers in the literature have compared the accuracy of existing indices for different populations of surgical patients.^42-44^ Most of these studies show that the various existing indices, although not very accurate, can predict events and should be used in perioperative assessment.

Among the risk indices with cardiovascular outcomes, we highlight the Lee’s Revised Cardiac Risk Index (RCRI),^45^ the index developed by the American College of Physicians (ACP),^46,47^ and the Multicenter Perioperative Evaluation Study (EMAPO-www.consultoriodigital.com.br^48^ - the Multicenter Perioperative Evaluation Study was developed and validated in the Brazilian population. All indices have advantages and disadvantages that must be considered during their use. When estimating risk, we should consider which outcome we are predicting: the ACP algorithm predicts the occurrence of AMI and cardiovascular death. The RCRI estimates the risk of AMI, acute pulmonary edema, total atrioventricular block, and cardiorespiratory arrest. The RCRI is widely validated in the literature and shows moderate level of accuracy in predicting events in noncardiac surgeries; this index is less accurate in patients undergoing arterial aortic vascular surgeries and peripheral revascularizations.^49^ Thus, in a specific evaluation guideline on risk assessment in patients undergoing vascular surgeries,^50^ the VSG-CRI (Vascular Study Group of New England Cardiac Risk Index) is proposed as an alternative to the RCRI; it is adapted from RCRI with additional variables.^51^

When the aim is to estimate global risk, not only related to cardiovascular morbidity and mortality, the ACS NSQIP Surgical Risk Calculator (www.riskcalculator.facs.org), which has been recently developed by the American College of Surgeons, can be used. This tool was developed using data from more than 1 million surgeries in 393 hospitals in the United States. It had good prediction accuracy in that population. This index includes, in addition to the specific type of surgical procedure, 21 clinical variables, providing a risk assessment of 8 different outcomes.^52^ On the other hand, this tool presents some limitations related to subjective variables and still needs validation in other populations.

As already discussed, risk indices have advantages and limitations, and none of them is perfect. It should be kept in mind that the risk index selected should be used as a complement, but never a replacement, to the evaluator’s opinion. Data or evidence is not always available in the literature for all situations. Thus, assessment should be customized. In those cases where the evaluating physician considers that the index is underestimating the actual risk, this observation should be mentioned in the evaluation.

**Figure 1:**
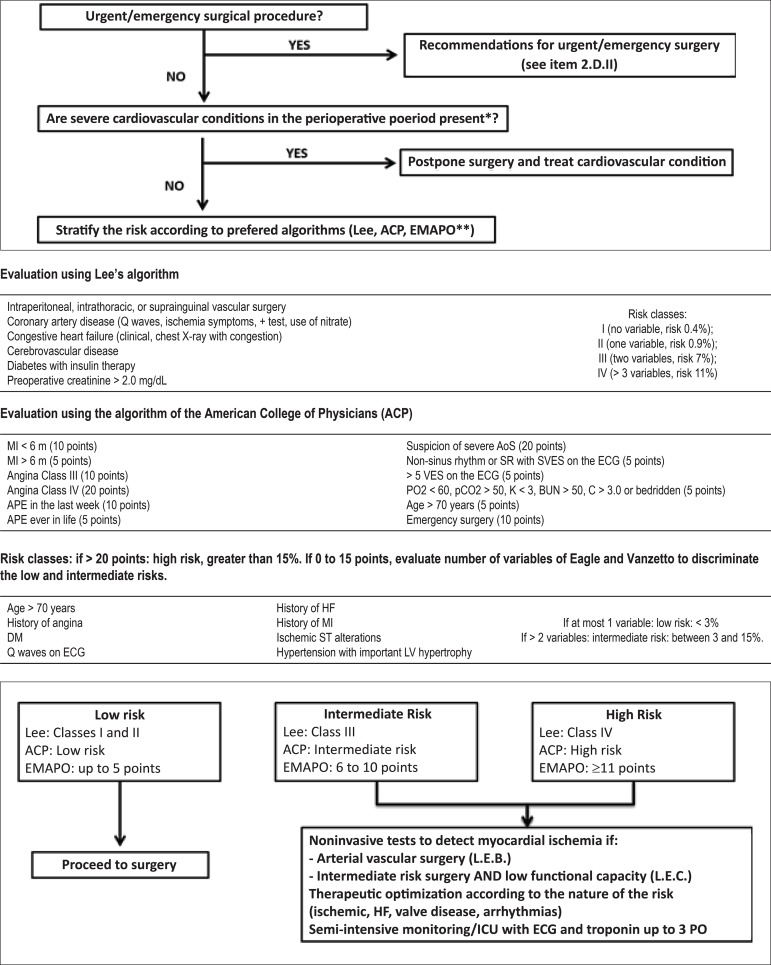
Flowchart of the III Guideline Perioperative Cardiovascular Evaluation

In addition to the risk indices already mentioned, other features related to surgical procedure and patient should be considered in the evaluation of the risk of perioperative events. We recommend using a flowchart proposed in this guideline (Figure 1).

#### II. Emergency and Urgent Surgeries *vs.* Elective Surgeries

In situations when the prognosis of the underlying disease that led to surgical indication demands an emergency intervention, the role of the cardiologist should be restricted to monitoring measures and interventions to reduce the risk in the intra and postoperative periods, with no indication of complementary tests that delay the proposed surgery. For urgent surgeries, there is time to optimize the cardiovascular therapy or to perform complementary tests, such as transthoracic echocardiography, when indicated (item 3.A). On the other hand, the request of functional tests to evaluate myocardial ischemia should not be performed, because the result will not change the plan and the proposed surgery cannot be postponed for 6 weeks (time required for preoperative myocardial revascularization or antiplatelet therapy, if indicated - see items 7.A.V and 7.B).

#### III. Severe Cardiovascular Conditions in the Perioperative

The first step in elective surgeries is the verification of the patient’s baseline clinical conditions. There are clinical circumstances in which the spontaneous risk of complications is very high, regardless of the surgical procedure. Identification of such conditions is fundamental, because their treatment should take priority over elective surgery, which should be, whenever possible, postponed and reconsidered only after clinical compensation (Chart 2).

#### IV. Intrinsic Risk of the Procedure

The intrinsic risk of the surgical procedure corresponds to the probability of occurrence of perioperative cardiovascular events, independently of the clinical variables of the patients. It is related to the duration of the procedure, hemodynamic stress, and loss of blood and fluids that occurs during the intervention. Patients with stable clinical conditions who do not present high-risk cardiac conditions may be referred for low intrinsic risk procedures without the need for further evaluation. Despite the difficulty in determining a specific risk for surgical procedures, since they occur in different circumstances, a risk classification of cardiovascular events (death or non-fatal AMI) was proposed for noncardiac surgeries (Chart 3).^53^

**Chart 3:** Classification of the intrinsic risk of cardiac complications of non-cardiac surgeries

HIGH (Cardiac risk > 5%)	Vascular surgeries (aortic and other major vascular surgery, peripheral vascular surgery)
	Urgent or emergency surgeries
**INTERMEDIATE (Cardiac risk between 1 and 5%)**	Carotid endarterectomy and endovascular repair of abdominal aortic aneurysm
	Head and neck surgery
	Intraperitoneal and intrathoracic surgeries
	Orthopedic surgeries
	Prostate surgeries
**LOW (Cardiac risk < 1%)**	Endoscopic procedures
	Superficial procedures
	Cataract surgery
	Breast surgery
	Outpatient surgery

Adapted from Fleisher et al.53

#### V. Functional Capacity

Patients with low functional capacity are more prone to perioperative complications.^18,54^ Functional capacity can be measured objectively using the exercise stress test (which is not always possible or desirable) or clinical history. Limitations in performing activities of daily living, such as walking quickly, climbing stairs, doing household activities or exercising regularly, are evaluated (Chart 1). In addition to the greater probability of poor perioperative evolution, patients with low functional capacity may have their symptoms underestimated due to their restrictions. Therefore, this can be considered when deciding to request complementary tests, such as, for example, non-invasive testing of ischaemic heart disease.

#### VI. Perioperative Evaluation Flowchart

Based on the above, the III Guideline for Perioperative Cardiovascular Evaluation of the Brazilian Society of Cardiology proposes a flowchart for perioperative evaluation by using the existing risk indices and relevant risk variables for this period (Figure 1). The algorithm contains the conditions that must be analyzed sequentially according to their relevance for the determination of the risk.

Depending on the estimated risk and the nature of the risk, interventions for clinical treatment or additional risk stratification with complementary tests are proposed. This applies to increased risk of ischemic events, decompensation of HF/valvular heart disease, and arrhythmias, considering the current specific guidelines for each case. As an example, if the nature of the risk is ischemic, non-invasive testing of ischaemic heart disease should be considered.

In addition, for patients classified as intermediate or high risk by the algorithms, surveillance for postoperative cardiac events must be performed, including, monitoring in a semi-intensive or intensive care unit (ICU), ECG and troponin being performed once daily up to the third postoperative day.

Perioperative evaluation is a unique opportunity to identify and advice patients about cardiovascular risk factors. During this period, diagnosis of previously unknown diseases, which can be optimized for a better perioperative evolution and, more importantly, for a better long-term prognosis, is often possible.^55^

## 3. Additional Preoperative Evaluation

### A) Evaluation of Ventricular Function at Rest

Resting echocardiography in the preoperative period of noncardiac surgery is not a routine test. However, in specific situations, it may offer additional risk information that may be useful for future therapeutic decisions. The use of this procedure in preoperative patients is to evaluate right and left ventricular dysfunction and signs of myocardial ischemia or valvular abnormalities, which are not detected previously in the clinical examination, chest X-ray, or even the ECG. Although controversial, it may be indicated in patients with a surgical risk that justifies this investigation.^56,57^

Transthoracic echocardiography is the main diagnostic method in patients with suspected or known HF. By using this method, including refined methods of analysis, such as myocardial strain imaging and three-dimensional echocardiography, we can accurately assess ventricular volume, ejection fraction, cardiac output, longitudinal strain, and degree of hemodynamic impairment. Assessment can be performed by determining the diastolic function and pressures in the pulmonary artery and left atrium using the E/e’ ratio and the presence and location of cardiac dys-synchrony in patients with a left ventricular ejection fraction < 35% or with a QRS > 120 ms.^58-60^ However, routine echocardiography is not indicated in all patients because no evidence exists to support that its use is associated with increased survival or shorter hospital stays. Several studies suggest that echocardiography increases hospitalization time, without leading to clinical benefit.^61^ Additionally, in patients with acute HF, clinical compensation should be performed, whenever possible, prior to the intervention.^62^

In patients with known or suspected valve disease, particularly those with moderate or severe aortic stenosis, severe mitral stenosis, severe mitral or aortic regurgitation, and those with intracardiac prostheses, transthoracic or transesophageal echocardiography should be used to determine the severity of the valve disease, help preoperative clinical treatment and guide prophylaxis or therapy for infective endocarditis (item 7.E).^63-67^

### B) Noninvasive Tests to Detect Myocardial Ischemia

#### I. Electrocardiogram Exercise Testing

The pathophysiology of perioperative MI differs from that of spontaneous MI. Perioperative MI can be caused by plaque rupture in approximately half of the cases or by imbalance between myocardial oxygen supply (anemia, low flow, etc.) and demand (tachycardia and hypertension).^68-70^

Exercise ECG testing is a safe, useful, and effective tool to detect myocardial ischemia, which is produced by an imbalance between supply and demand. Therefore, it is reasonable to assume that detection of abnormalities while performing this test may be reproducible during the perioperative period and its varying levels of stress. However, whether this strategy leads to reduction of perioperative cardiovascular events in all cases is unknown. It should also be considered that lower prevalence of coronary disease in a population results in lower positive predictive value of the exercise ECG testing.

Considering that risk stratification aims to reduce perioperative risk, performing the test in a population already stratified as low risk by the recommended algorithms is not logical. Therefore, in cases of low prevalence of coronary artery disease (CAD), the exercise ECG testing would not add value to the perioperative clinical stratification. It could also delay the surgery and require more specific tests to differentiate the true results from the false-positive.^71,72^

Even in high-risk individuals, such as those undergoing preoperative vascular surgery, the predictive value, sensitivity, and specificity of the exercise ECG testing (10%, 74%, and 69%, respectively) are low, but with a high negative predictive value (98%).^73^ On the other hand, in a cohort study, performance of provocative ischemic preoperative tests in high-risk patients, with three or more clinical risk factors, is associated with shorter hospital stays and lower mortality.^71^ Thus, among asymptomatic individuals with a higher prevalence of the disease, the exercise ECG testing could be requested only if the result would influence the prognosis and, consequently, preoperative decisions, or to provide a more intensive clinical therapy or even a myocardial revascularization procedure.^57^ In this case, the onset of ischemic response at low load is associated with a higher number of perioperative cardiac events. On the other hand, patients with exercise tolerance up to 4-5 METS have a good perioperative prognosis.^65,74^

#### II. Stress Myocardial Perfusion Scintigraphy

The exercise ECG testing is safe, useful, and effective to detect myocardial ischemia and has a good cost-risk-benefit ratio,^75^ but has limitations, such as patients with physical limitations, patients who present abnormal ST-segment changes or left bundle bock in the baseline ECG. The alternative for such patients is an imaging method with pharmacological stress (adenosine, dobutamine, or dipyridamole).

In this context, myocardial perfusion scintigraphy (MPS), when possible associated with exercise and, within the physical limitations, pharmacological stress, has a good accuracy and prognostic value.^70^ In a meta-analysis of 1,179 patients submitted to vascular surgery, MPS with dipyridamole demonstrated a greater number of perioperative cardiovascular events, proportional to the presence and extent of perfusion defects. Those with reversible ischemia in up to 20% of the left ventricular extension had the same events with those without ischemia. However, when the area affected was 20-29%, 30-49%, and above 50%, the probability of cardiac events was 1.6, 2.9, and 11 times higher, respectively.^76^

Another meta-analysis with the same method and similar profile of patients showed that patients without perfusion defect, with fixed defect, and with reversible defect presented mortality and nonfatal MI rates of 1%, 7%, and 9%, respectively. Patients with two or more perfusion defects have a high incidence of cardiac events.^77^

Gated-associated MPS, which allows assessment of both myocardial perfusion and cardiac function, has been recently shown to be a useful tool for risk stratification in vascular surgeries. In one study, abnormal final systolic volume (more than twice the standard deviation) was the only independent variable to predict cardiac events. Patients with normal perfusion but with changes in contractility had significantly more cardiac events than those with normal contractility and perfusion (16% x 2%; p < 0.0001).^78^

In conclusion, in the perioperative evaluation, the indications for Gated-associated MPS are similar to those of the exercise ECG testing. It is the best option for patients with physical limitations. It is also the best alternative when the ECG is impossible to interpret due to baseline changes of the ST-segment and when the result of the exercise ECG testing is a possible false positive

#### III. Stress Echocardiography with Dobutamine

Stress echocardiography is an accurate and reliable tool to identify patients with CAD and has an important role in the prognosis of cardiac events^79,80^

Dobutamine and exercise stress echocardiography have similar diagnostic accuracies, which are higher than that of dipyridamole stress.^81^ If a dobutamine stress echocardiography does not demonstrate the presence of residual ischemia in a patient with a history of infarction, the prognosis is good and the probability of reinfarction, death, and acute pulmonary edema is low in the perioperative period of a noncardiac surgery.^73^

The use of dobutamine stress echocardiography in perioperative risk assessment is already well documented in the literature, with a positive predictive value ranging from 25-55% and a negative predictive value of 93-100% for cardiac events in patients undergoing noncardiac surgery,^73,82^ The results were generally used to determine preoperative clinical decisions, particularly the decision to perform coronary angiography or myocardial revascularization before or after the elective surgery.

A meta-analysis of 15 studies was performed to compare dipyridamole thallium-201 and dobutamine stress echocardiography in vascular risk stratification before surgery. It demonstrated that the prognostic value of the abnormalities is similar in both imaging methods for perioperative ischemic events.^77^

### C) Invasive Coronary Angiography

Coronary angiography is a well-established invasive procedure, but it is rarely indicated for risk assessment in noncardiac surgeries. The available data are insufficient to recommend the use of coronary angiography in all patients (i.e., routine tests), including those undergoing high-risk surgeries. In general, the indications for coronary angiography in the preoperative period are similar to those for angiography in other situations. In addition, invasive coronary angiography assessment may cause unnecessary and unpredictable delay to an already scheduled surgical intervention, as well as add the risk of the procedure.^83^ Notably, in services where non-invasive tests are unavailable for the detection of myocardial ischemia, coronary angiography should not be requested as an alternative to these tests.

### D) Additional Tests

#### I. Coronary Computed Tomography Angiography

Coronary Angio-CT has been increasingly used to evaluate patients with suspected CAD. It presents high sensitivity for the detection of coronary stenosis, including multiarterial disease and lesion in the left coronary trunk.^84-87^ However, it has not been extensively investigated in the perioperative period of noncardiac surgeries.

Ahn et al.^88^ analyzed retrospective data and concluded that angiotomography may be advantageous in reclassifying the risk of patients assessed by Lee’s revised score (RCRI), when submitted to intermediate risk procedures.^45,88^ On the other hand, a cohort showed a fivefold higher probability of overestimating the risk of angiotomography in patients who present an event.^89^

A few studies have shown an association between elevated coronary calcium score and cardiovascular events. Angiotomography could still be applied as an instrument for risk reclassification.^88,90^

Nevertheless, the information obtained through such tests was still not correlated with new interventions (revascularization, pharmacoprotection, or monitoring) to reduce perioperative coronary events. Therefore, angiotomography or coronary calcium score is not recommended in the preoperative period.^91^

#### II. Ankle-brachial Index

The ankle-brachial index (ABI) is one of the preferred methods for the diagnosis of peripheral occlusive arterial disease (PAOD). Values ≤ 0.9 show good accuracy for the diagnosis. ABI is associated with poor cardiovascular prognosis, significantly increasing the risk of amputation, CAD, and cerebrovascular disease.^92,93^ It can be used as a risk reclassification tool in conjunction with the Framingham risk score, increasing the mortality risk due to all causes in all risk categories.^94^ Although ABI is a promising method because of its low cost, rapid standardization, good acceptance by patients, and low intra- and inter-observer variability, it is poorly investigated in the perioperative context.^95^ Flu et al.^96^ showed that patients with ABI ≤0.9 submitted to vascular surgery had an odds ratio (OR) of 2.4 for the occurrence of myocardial injury. Two other studies evaluated ABI in patients undergoing noncardiac and nonvascular surgeries and obtained ORs of 10.2 and 7.0 for the occurrence of major cardiovascular events, including isolated increase of troponin.^97,98^

Association of this test with perioperative risk scores has not yet been studied, and the risk reclassification capacity is unknown. Therefore, routine use of ABI is not recommended as a risk estimation tool. For patients with previously known vasculopathies, focusing on the pharmacological prevention of cardiovascular events and monitoring, as discussed in other topics of this guideline, is recommended.

#### III. Holter

Holter is a continuous electrocardiographic monitoring tool that identifies the presence of atrial and ventricular arrhythmias and their complexity. It also identifies dynamic changes of the ST-segment that are indicative of myocardial ischemia. This method is not routinely used in the evaluation of preoperative ischemia, because other diagnostic methods are more sensitive and specific for this purpose. Its possible application during the perioperative period is monitoring ischemic events that occur in the intra- and postoperative periods, which can have a particularly high incidence in some specific groups of patients.

Electrocardiographic monitoring in the postoperative period using Holter was not very sensitive (50%), although very specific (92%), for the diagnosis of reinfarction in patients undergoing noncardiac surgeries and who had a previous history of myocardial infarction.^99^ Therefore, routine use of this test is not recommended. Requesting a Holter in the preoperative period follows the same indications of other contexts.

### E) Biomarkers

#### I. Cardiac Troponins

The increased sensitivity of the available troponin kits provided a greater accuracy and rapidity in the diagnosis of MI in patients with chest pain in the emergency room.^100^ On the other hand, in the perioperative period of noncardiac operations, the available scientific evidence does not include all troponins (I and T) or all assays used, which have different detection limits and reference values. For this reason and due to the importance of this diagnostic tool, we decided to include a detailed explanation of the available methods before the recommendations in this guideline.

The detection limit represents the minimum value that is detected by the method. The reference value of normality is determined using the 99th percentile, which is obtained by performing the test in a normal population, and indicates that 99% of normal individuals have values below this cut-off.

Troponin assays can be classified as low sensitivity (conventional), medium sensitivity (contemporary or sensitive), or high sensitivity. This classification is based on the percentage of healthy individuals in whom troponin can be detected. Contemporary troponin assays (medium sensitivity) can detect values above the 99% percentile (altered values); however, troponin is only detected in a few normal individuals. High sensitivity assays can determine values of this marker (limit of detection) in 50-95% of normal individuals.^101^

Table 1 presents some troponin assays and their classification and respective reference values of normality.^101,102^ To properly interpret and request troponin as a biomarker in the perioperative period, the physician must be familiar with the troponin assay used in their hospital. Notably, in the preoperative period, only the Roche high-sensitivity troponin T (hs-TnT) assay has been tested in the available studies and may have clinical applicability. In the postoperative period, most studied troponins are conventional and some are sensitive (as specified in item 7.F).

**Table 1:** Troponin assays, sensitivity, and reference values

Troponin assay	Detection limit (ng/L)	Reference value (99th percentile) – (ng/L)
Conventional (low sensitivity) *		
Troponin T 4th generation Roche Elecsys	10	Unknown **
**Contemporary (medium sensitivity/sensitive)**		
Troponin I Siemens ADVIA Centaur Ultra s-cTnI	6	40
Troponin I Abbott Architect s-cTnI	9	28
Beckman-Coulter Access Accu-cTnI	10	40
Troponin I Roche Elecsys TnI	100	160
**High sensitivity (hs)**		
Troponin T hs-TnT Roche Elecsys	5	14
Troponin I Siemens Dimension Vista hs-TnI	0,5	9
Troponin I Abbott Architect hs-cTnI	1,9	26,2
Beckman-Coulter Access hs-cTnI	2	9,2

* No longer used in modern hospitals;

** Most individuals have values below the detection limit, thus, the 99th percentile is impossible to determine.

Some published papers demonstrate the efficacy of high sensitivity preoperative troponin as a marker of perioperative cardiovascular complications and general mortality in noncardiac surgeries. Nagele et al.^103^ studied 608 patients who underwent noncardiac surgery. They showed that 41% had increased values of hs-TnT above the 99th percentile (> 14 ng/L) in the preoperative period. The increase in preoperative hs-TnT was associated with a higher total mortality during a 3-year follow-up.^103^ These findings were confirmed in a study involving 455 patients undergoing vascular surgeries. In this study, patients with increased hs-TnT in the preoperative period presented a greater number of cardiovascular events in the postoperative period.^104^ In a comparison to Lee’s RCRI algorithm in 979 patients aged more than 55 years with at least one cardiovascular risk factor submitted to noncardiac surgeries, preoperative hs-TnT presented an area under the ROC curve (0.78) similar to the RCRI (0.68; p = 0.07) in predicting combined cardiovascular events (mortality, MI, recovered cardiac arrest, and acute HF). In addition, in a multivariate analysis, increased hs-TnT in the preoperative period was an independent predictor of these combined events (HR 2.6; p = 0.008). As for general mortality, hs-TnT was superior to RCRI (area under the curve 0.81 × 0.66, p = 0.006).^105^ The prevalence of preoperative hs-TnT increases varies between 21% and 41%, depending on the age and risk factors, such as diabetes, CAD, systemic arterial hypertension, and renal failure.^103-107^

No study has evaluated the role of high-sensitivity troponin I (hs-TnI) in predicting cardiovascular events in the preoperative period. Contemporary/sensitive TnI was evaluated in 560 patients undergoing noncardiac surgeries, with only 5% presenting preoperative values above the 99th percentile. Its use did not improve the prediction of risk of perioperative cardiovascular events.^108^

In conclusion, measurement of troponins with conventional or contemporary/sensitive assays is not useful in the preoperative period and should not be performed. On the other hand, the hs-TnT measurement in the preoperative period can be used as a tool for risk stratification associated with the use of the algorithms. In addition, these data help to establish a baseline value in patients with indication for postoperative monitoring, facilitating the interpretation of postoperative values of hs-TnT and the diagnosis of postoperative MI (items 7.F and 8.A).

#### II. Natriuretic Peptides

OThe risk scores usually used in the perioperative evaluation enable to estimate the risk of cardiovascular events in the perioperative period with moderate accuracy. Tests to evaluate ischemia, as well as biomarkers (troponins and natriuretic peptides), allow to refine the risk assessment before surgery.^109^

Natriuretic peptides are released into the bloodstream by the heart in response to multiple physiological stimuli, such as myocardial stress and ischemia. Several studies demonstrated that high preoperative levels of BNP are potent predictors of perioperative cardiovascular complications.^110^

The two studies by Biccard et al.^111^ conducted investigations on patients submitted to arterial vascular surgery. In 2011, they reported that preoperative BNP was an independent predictor of increased postoperative troponin levels in a cohort of 267 patients undergoing vascular surgery. They also reported that the use of this biomarker improved the risk prediction of Lee’s RCRI in 38-70% in patients classified as intermediate risk.^111^ In 2012, the authors evaluated 788 patients undergoing vascular surgery and showed that increased preoperative BNP levels was an independent predictor of cardiac events in a period of 30 days (OR = 5.0; p < 0.001).^108^

A meta-analysis involving individual data from six different studies evaluated natriuretic peptides as predictors of events in patients undergoing vascular surgeries. The study confirmed that increased preoperative natriuretic peptide level is an independent predictor of events (cardiac death or nonfatal MI) in up to 30 days after surgery. It was also observed that it improves the predictive value of the RCRI.^112^

With regard to patients undergoing nonvascular surgeries, there are no studies that exclusively evaluate this population. The vast majority of the studies involve both vascular and nonvascular surgeries.

In a meta-analysis published in 2009, including 15 prospective observational studies and 4,856 patients submitted to vascular or nonvascular surgeries, the authors found that increased preoperative BNP or NT-proBNP levels was associated with a high (nearly 20-fold higher) risk of major cardiovascular events, cardiac mortality, and mortality due to all causes (almost 10-fold) in the perioperative period (< 43 days after surgery).^113^ However, whether prognostic information was improved in these studies, considering the existing risk indices, were not determined.^114^

More recently, a prospective multicenter observational study analyzed 979 patients aged more than 55 years with at least one cardiovascular risk factor (hypertension, diabetes, dyslipidemia, smoking, family history) in the preoperative period of noncardiac surgery. The study aimed to assess increases in hs-Tn as a risk predictor compared to the Lee’s RCRI, with 93% of the patients with RCRI < 2. In this study, the authors also evaluated the role of natriuretic peptides as risk predictors in noncardiac surgery. They observed that both hs-Tn levels and NTproBNP levels were higher in individuals with cardiovascular events and seemed to provide additional information to the RCRI.^105^

Finally, a meta-analysis conducted in 2014 included 18 prospective observational studies. The authors assessed individual data from 2,179 patients undergoing noncardiac (vascular and nonvascular) surgery. They demonstrated that postoperative levels of BNP and NTproBNP provide additional risk prediction of death or nonfatal infarction in a period of 30 and 180 days to a risk prediction model that includes preoperative BNP. They also showed that high values of BNP/NTproBNP both in the postoperative (OR = 3.7; p < 0.001) and preoperative (OR = 1.9; p < 0.001) periods were independent predictors of death or nonfatal MI in a period of 30 days.^115^

Therefore, we consider that BNP/NTproBNP measurement can provide additional prognostic information on the risk stratification of patients undergoing vascular and nonvascular arterial surgeries.

## 4. Diseases and Conditions with Specific Features in the Perioperative Period

### A) Coronary Artery Disease

Objectively discriminating the surgical risk for each specific coronary artery disease (CAD) condition is critical for preventing and reducing morbidity of perioperative events. About four decades ago, perioperative risk analysis of CAD patients consisted of measuring the temporal relationship between a given cardiac ischemic event and the proposed surgery. At present, in addition to the mentioned interval,^116^ we consider all the relevant factors for the prognosis of patients with CAD, independently of the perioperative context. These factors include presence of angina, HF, electrocardiographic signs, extent, and threshold of ischemia, and coronary anatomy, if relevant. The routine and indiscriminate performance of additional tests, such as functional tests and invasive coronary angiography, has no proven benefit, even in the population already diagnosed with CAD. A careful anamnesis associated with propaedeutics of the circulatory system and basic additional tests, such as rest ECG and chest X-ray, is often sufficient to determine the surgical risk of CAD patients. The request for functional tests must comply with the indications mentioned in item 3.B.IV.

### B) Systemic Arterial Hypertension

Systemic arterial hypertension is a very common clinical condition, not only in the general population but also in patients who undergo surgical procedures. This condition, especially if uncontrolled, is one of the most common reasons for cancelling surgery.^117^ This is because systemic hypertension is associated with increased perioperative mortality.^118^ On the other hand, a study of carotid endarterectomy suggested that a patient with hypertension under control may not have an increased risk of perioperative morbidity and mortality, which suggests the importance of adequate control.^119^

Major hemodynamic changes can occur during a surgical procedure, being more pronounced in patients with hypertension. These hemodynamic changes, which are associated with pain and anxiety, are aggravated by withdrawal of antihypertensive drugs on the day before the procedure.^120^ Increasing knowledge on the pathophysiology of hypertension and antihypertensive therapy, as well as the development of new anesthetics and muscle relaxants with minimal hemodynamic effects, in addition to postoperative pain control protocols, have contributed to minimize the occurrence of complications during the perioperative period of hypertensive patients.

One of the mechanisms involved is the sympathetic activation observed during anesthetic induction and in the postoperative period. Increases in sympathetic activity may cause significant increases in blood pressure, particularly in patients with uncontrolled arterial hypertension. Supporting the importance of sympathetic hyperactivity, evidence suggests that clonidine, when used in the perioperative period of hypertensive patients, significantly reduces blood pressure and heart rate variation, as well as reduces the need for anesthetic (isoflurane) and narcotic administration in these patients.^121^ Despite this, no evidence is available on the choice of antihypertensive agents in the perioperative period.^120^

In general, stage 2 hypertension with SBP > 180 mmHg and DBP > 110 mmHg must be controlled before surgery. However, in mild or moderate hypertension, in which metabolic or cardiovascular changes do not occur, no evidence has been established on the benefits of postponing surgery.^57,120^ The perioperative strategy should generally be to maintain blood pressure within 20% of the preoperative values (provided this value is not too uncontrolled), which implies flexibility in the control, not necessarily to normal levels. This may reduce the occurrence of hypertensive emergencies in the perioperative period.

Patients with some degree of autonomic dysfunction (such as that in hypertensive patients) are more susceptible to intraoperative hypotension than normotensive patients. This phenomenon appears to be more frequent in patients who use angiotensin-converting enzyme (ACE) inhibitors in the preoperative period. In most cases, this may be related to reduced intravascular volume; thus, avoiding perioperative hypovolemia is fundamental. However, abrupt withdrawal of these drugs should not be performed, because both uncontrolled blood pressure and decompensated HF may increase the risk of cardiovascular complications.

Patients with suspected secondary hypertension should be investigated prior to surgery, except in urgent/emergency cases. There is no conclusive evidence on the increase in perioperative risk in patients with secondary hypertension; however, patients with undiagnosed pheochromocytoma have a mortality rate of approximately 80% during surgery.^122^

Cardiac and blood pressure monitoring in hypertensive patients is essential during the surgical procedure to detect variations of blood pressure and signs of ischemia as early as possible. In addition to being a risk factor for CAD, hypertension is associated with ventricular hypertrophy, systolic dysfunction, renal failure, and cerebrovascular events during the perioperative period. This aspect should be considered in the perioperative volume management of hypertensive patients with altered ventricular geometry and arterial elasticity, particularly the elderly.^123^ In the intraoperative period, the ideal antihypertensive agent should be easily titrated, rapid acting, have minimal side effects, and inexpensive. Several antihypertensive classes are available, including β-blockers (esmolol, labetalol), calcium channel blockers (nicardipine), and nitrates (sodium nitroprusside and nitroglycerin).

### C) Heart Failure

HF affects about 1-2% of the general population in developed countries and more than 10% of the population aged more than 70 years,^65,124^ CVDs are the main cause of death in Brazil, representing approximately 29% of the deaths in the country. Ischemic heart disease and HF are responsible for approximately 39% of these CVD deaths.^125^

HF is a well-known risk factor for perioperative cardiac events. Data from a large registry of noncardiac surgeries, which included more than 150,000 procedures, revealed that the presence of HF was associated with a 63% increase in the risk of perioperative mortality and a 51% increase in the risk of rehospitalization in a period of 30 days, when compared to the group with CAD without HF.^126^

Reduced ejection fraction is considered a strong predictor of events in patients undergoing vascular surgery. However, most of studies analyzed ejection fraction as a categorical variable (higher or lower than 40%). A recent study involving 174 patients with HF revealed that only severely reduced ejection fraction (< 30%) is an independent predictor of mortality. Moderate (30-40%) or mild (40-50%) ejection fraction reduction and preserved ejection fraction HF (> 50%) were not independent predictor of death in a period of 30 days.^127^ Despite the predictive value of ejection fraction, performing routine echocardiography for all patients undergoing noncardiac surgery is not indicated. A Canadian cohort study involving more than 250,000 patients (15% with preoperative echocardiography) revealed that preoperative echocardiography is not associated with improvement in survival or reduction of hospitalization time after major noncardiac surgery.^61^

Increased level of natriuretic peptides in the preoperative period is related to worse prognosis in the perioperative period, because it is related to worsening of ventricular function and higher rate of cardiovascular events.^128,129^ Measurement of these biomarkers may aid in the risk stratification of patients with HF. However, clinical evaluation and functional condition are even more relevant in the perioperative assessment of patients with HF.

Clinical management in the perioperative period requires special care regarding the blood volume of the patient. Both hypovolemia, which may intensify hypotension, and hypervolemia, which may lead to pulmonary and systemic congestion, should be avoided.

Patients with preserved fraction HF due to increased left ventricular stiffness are also susceptible to pulmonary edema, secondary to volume overload. Therefore, the use of diuretics and vasodilators may be necessary to avoid hypervolemia and afterload increases.

### D) Valvular Heart Disease

Patients with valvular heart disease have a higher risk of presenting cardiovascular complications in the perioperative period of noncardiac surgeries.63 The risk varies depending on the valvular heart disease and its anatomic severity, as well as the type of noncardiac surgery to be performed.^45^ The main cardiovascular complications in the perioperative period of noncardiac surgeries in patients with valvular heart disease are as follows: pulmonary congestion/acute pulmonary edema, cardiogenic shock, MI, tachyarrhythmias, embolic events, bleeding, and infective endocarditis.^130,131^ In patients with valvular heart disease, particularly if anatomically relevant, clinical evaluation performed by a cardiologist should be considered in the preoperative period of noncardiac surgeries.

When valvular heart disease is suspected after detailed medical history and physical examination, transthoracic echocardiography should be performed with the following objectives: quantify the anatomic severity of valvular disease, assess ventricular function and remodeling of cardiac chambers, and estimate right chamber pressure. If doubt persists, other diagnostic methods may be performed, such as transesophageal echocardiography, magnetic resonance imaging, computed tomography, and cardiac catheterization.

Stenotic valvular heart disease have a higher perioperative risk than regurgitant valvular heart disease . Therefore, additional care should be given to patients with aortic (AoS) or mitral (MS) stenosis undergoing noncardiac surgery.

Symptomatic patients with anatomically relevant valvular heart disease already present high morbidity and mortality in the history of valvular heart disease and are indicated for interventional valve treatment.^132,133^ This patient group presents a high risk of perioperative cardiac complications if submitted to noncardiac surgery. Therefore, they should initially treat valvular heart disease and subsequently undergo noncardiac surgery. However, if the noncardiac surgery is an emergency, it should be performed without prior correction of valvular heart disease, even if it is anatomically relevant.

The use of statins in the perioperative period of patients with valvular heart disease has not been evaluated in prospective studies. Therefore, statins should not be prescribed without another indication. Similarly, the use of nitroglycerin or even the use of intraoperative cardiac output monitoring has not been evaluated in these patients.

On the other hand, although there is no formal indication for the use of β-blockers in valvular heart disease patients in the perioperative period of noncardiac surgeries, these drugs can be used in patients with MS.

#### I. Aortic Stenosis

AoS is the most common valvular heart disease in elderly patients, affecting 2-4% of adults aged more than 65 years. The prevalence of AoS is expected to double in the next 20 years, with the progressive aging of the population.^134,135^

Several studies have shown that patients with moderate to severe AoS may have a high risk of cardiac complications during noncardiac surgery.^19,136-139^ However, in many of these studies, the definition and degree of AoS were ambiguous or based on few details. Since most studies included symptomatic or ventricular dysfunction patients, it is unclear whether aortic valve replacement surgery should precede the noncardiac procedure. The study by Calleja et al., which included 30 patients with anatomically relevant AoS and 60 controls (mild to moderate AoS), was the only one to exclude symptomatic patients. They showed similar MI or death rates during noncardiac surgery, mostly with low or intermediate risk.^64^

For these reasons, Samarendra et al. recommended a new flowchart to assess the perioperative risk of noncardiac surgeries in patients with AoS. Patients with a high risk of cardiac complications are those with an average gradient > 45-50 mmHg and/or valvular area < 0.8 cm^2^; left ventricular systolic dysfunction; symptomatic AoS; significant mitral regurgitation or other associated valvular diseases; increase in ≥ 18 mmHg in the average gradient during exercise; and associated CAD.^67^

Furthermore, in a recent study with 218 patients, Mizuno et al.^140^ demonstrated that patients with AoS who underwent a major noncardiac surgery present a faster progression of aortic valve disease compared to controls with AoS who did not undergo surgical intervention.

For the above reasons, it is recommended to first correct the anatomically relevant AoS, even if asymptomatic, in patients who will undergo intermediate- or high-risk noncardiac surgeries.

On the other hand, some patients with relevant AoS scheduled for noncardiac surgery have clinical indication for correction of valvular heart disease , but present a high risk for cardiac surgery or are ineligible for the conventional cardiac procedure. For these cases, a transcatheter aortic valve implantation (TAVI) is an option preceding noncardiac surgery.^141-143^

When patients with relevant AoS are submitted to urgent/emergency noncardiac surgery, preoperative clinical compensation is recommended with the use of diuretics, as well as postoperative period in an ICU, with hemodynamic and electrocardiographic monitoring and serial measurement of myocardial necrosis markers.

#### II. Mitral Stenosis

Patients with MS and formal indication for surgical or percutaneous correction of valvular heart disease should be submitted to the valve procedure before elective noncardiac surgery.^144^ If noncardiac surgery is an emergency, it can be performed with invasive hemodynamics monitoring, optimization of blood volume, and prevention of tachycardia and hypotension. Increased heart rate, particularly if there is development of atrial fibrillation (AF), can lead to congestion and pulmonary edema. Therefore, β-blockers and/or diuretics may be used during the perioperative period.

#### III. Aortic Insufficiency and Mitral Insufficiency

Regurgitant valvular heart disease are associated with increased cardiac risk during noncardiac surgery, but are better tolerated than stenotic valve lesions.^145,146^ However, aortic insufficiency (AoI) and mild or moderate mitral insufficiency (MI) do not increase the risk of cardiovascular complications during noncardiac surgery.

On the other hand, patients with symptomatic AoI or MI associated with ventricular dysfunction have a high risk of cardiovascular complications, and valvular heart disease should be performed before elective noncardiac surgery. Urgent or emergency noncardiac procedure should be performed after optimization of pharmacological treatment and optimal hemodynamic stability through the preferential use of vasodilators and diuretics, in addition to the postoperative period in an ICU.

#### IV. Valve Prosthesis

Patients with valve prostheses with normal function, without left ventricular dysfunction, may undergo noncardiac surgery without additional risk. For mechanical prosthesis, oral anticoagulation and bridge with heparin should be performed as described in section 7.D.^132^

### E) Cardiac Arrhythmias

Cardiac arrhythmias are common in patients with or without structural cardiopathy. The impact on morbidity and mortality in the perioperative period is mainly related to the underlying diseases, because arrhythmias that occur in patients without structural cardiopathy generally do not increase the risk of cardiac complications.^45^ This distinction must be made by the cardiologist before the elective procedures.

There is limited data in the literature on the real impact of arrhythmias in this period, impairing the selection of a specific approach. Therefore, the recommendations are usually extrapolated from routine evaluation and outpatient or emergency decisions.^147^

The factors that can trigger supraventricular and ventricular arrhythmias and should be investigated include electrolyte imbalances (hypokalemia, hypomagnesemia, hypocalcemia), hypoxemia, proarrhythmic drugs (antidepressants, stimulants, positive inotropes, anesthetics), and metabolic disorders (hyperthyroidism or hypothyroidism). The priority is the correction of reversible factors in the preoperative or intraoperative period in cases of urgent or emergency surgery.

#### I. Paroxysmal Supraventricular Tachycardia

Supraventricular paroxysmal tachycardias are more prevalent in structurally normal youngsters and rarely present hemodynamic intolerance. They can occur by atrial tachycardia, ortho-ventricular atrioventricular tachycardia (in patients with accessory pathway), and nodal reentrant tachycardia. Asymptomatic patients who present ECGs with ventricular pre-excitation also have a low risk of perioperative complication. Attention should be only given to the occurrence of supraventricular tachycardia and pre-excited AF, and treatment follows the ACLS standard care.^148^

In this period, occurrence of pain, nausea, gagging, hypothermia, sympathetic blockade in anesthesia, laparoscopic insufflation, laryngoscopy, hyperventilation, anesthetics, and cholinergic drugs may precipitate arrhythmia due to autonomic imbalance. Such stimuli may trigger supraventricular tachycardias but do not increase surgical morbidity and mortality.

Controlling triggering factors mentioned above can minimize the occurrence of arrhythmias. In patients taking antiarrhythmic drugs, these drugs should be continued because preoperative interruption may promote arrhythmia.

#### II. Ventricular Extrasystoles and Tachycardias

Detection of extrasystolic arrhythmias in the preoperative period is common in high-risk patients. On the other hand, about 20% of the population can present these arrhythmias in the 24-hour Holter exam performed routinely.

Evaluation of extrasystolic arrhythmias includes collecting personal and family history. The occurrence of symptoms of hemodynamic intolerance (syncope or pre-syncope, precordial pain) may indicate complex or sustained arrhythmias. The presence of family history of sudden cardiac death may indicate the need for specific evaluation. In asymptomatic young patients with no personal or family history of heart disease, isolated monomorphic ventricular extrasystoles may generally be benign and with no implications in the perioperative period. In low-risk surgeries, evaluation of the ECG and cardiac area by chest X-ray may be sufficient and suspension of noncardiac surgery is unnecessary.^149^

Suspicion of adjacent structural disease can be detected in ECG. ECG can identify ventricular overload, inactive area, conduction system diseases, and other rarer arrhythmic syndromes. Thoracic radiography is an important screening procedure for pulmonary diseases and detection of increased cardiothoracic index. Echocardiogram is a sensitive method that should be used for additional morphological analysis. An exercise ECG testing may be useful to demonstrate the presence of ischemia as a triggering factor for ventricular arrhythmias or to demonstrate benignity when dealing with idiopathic arrhythmias, which are suppressed by sinus tachycardia at the peak of the exercise.^149^

Monomorphic ventricular tachycardia (VT) is often due to myocardial scarring. Polymorphic tachycardia may indicate ischemia, which reinforces the need for further investigation according to the specific guidelines.^149^

The first preoperative treatment of ventricular arrhythmias is correction of reversible causes. There is no evidence that unsusceptible extrasystoles or VTs worsen the perioperative prognosis, nor is there is a proven benefit of suppression with antiarrhythmics.^150^

#### III. Atrial Fibrillation and Flutter

AF is the most common sustained tachyarrhythmia and its prevalence increases with age. Patients who present a previous diagnosis of AF with adequate clinical control, considering symptoms and basal heart rate, do not need special considerations, except recommendations on anticoagulation (see item 7.D).^151^

In the preoperative period of elective surgeries, β-blockers or non-dihydropyridine calcium channel blockers (verapamil and diltiazem) are essential to control heart rate when patients have persistent AF/Flutter with high ventricular response. Administration of these drugs should preferably be performed slowly to avoid hypotension, which is known to be deleterious postoperatively. Calcium channel blockers may cause depression of myocardial function, particularly in patients with structural cardiopathy.^152^ Digoxin may be attenuated by surgical hyperadrenergic condition.^65^ There are specific situations, such as pre-excited AF, in which ablation can be considered before surgery.^153^ It should be noted that patients with AF with ventricular response above 120 bpm have a severe cardiovascular condition. In these cases, surgery should be postponed until the heart rate is controlled.

Control of rhythm, i.e., reversal of AF, could be an option before the procedure, depending on the symptoms and specific evaluation by cardiologists based on the current guidelines. However, interruption of anticoagulation for the procedure could only be performed after 4 weeks, and perioperative adrenergic stress facilitates recurrence.^152,154^

Regardless of the control strategy selected (rhythm or heart rate), patients should be assessed for risk of cardioembolism and risk of bleeding using the CHA2DS2Vasc and HAS-BLED scores, respectively.^152^

#### Prevention of Postoperative Atrial Fibrillation in Patients with Sinus Rhythm

The occurrence of postoperative AF (POAF) is associated with increased time in ICU, increased morbidity (including stroke, with incidence of 1.3-1.7%) and mortality, and consequently increased hospital costs. The AF preventive measures are adequate hydroelectrolytic control in the pre- and postoperative periods (normovolemia, magnesium and potassium monitoring, and replacement), in addition to maintenance of drugs previously used if hemodynamically tolerated.^155^

Some drugs have been investigated to reduce the incidence of POAF and its deleterious consequences. Preventive antiarrhythmic therapy with amiodarone or venous magnesium should be discussed individually. Retrospective studies showed that the use of amiodarone for anesthetic induction in patients submitted to esophagectomy may reduce the rate of POAF, but did not reduce the mortality and hospitalization rates. Moreover, its use in patients submitted to pulmonary resection is associated with reduced POAF and decreased time in ICU.^156-158^ Riber et al.^159^ conducted a randomized, double-blind, placebo-controlled study. They demonstrated that administration of 300 mg of intravenous amiodarone in the early postoperative period, followed by 1200 mg of oral drug per day for 5 days, in hemodynamically stable patients reduced the POAF rate (9% versus 32% in the control group). On the other hand, Khalil et al.^160^ compared the use of amiodarone in the immediate postoperative period for 48 hours (attack of 5 mg/kg, followed by 15 mg/kg) with venous magnesium sulfate (attack of 80 mg/kg, followed by 8 mg/kg/h) and with a control group from a retrospective analysis in patients submitted to pulmonary resection. Their results showed POAF rates of 10, 12.5, and 20.5%.

Other medications were studied to reduce the incidence of POAF. A meta-analysis with statins showed a potential role in the prevention of AF, although, of the 16 trials included, only 4 were conducted in noncardiac surgeries.^161^ Thus, its benefit to this population remains inconclusive.

Colchicine, an anti-inflammatory drug, is being studied for prevention in high-risk patients submitted to thoracic surgery.^155^

#### IV. Hereditary Arrhythmias

Genetically determined arrhythmias are a heterogeneous group of diseases and may occur due to defects in ion channels (channelopathies). Protein defects lead to atrial or ventricular arrhythmias, which may be manifested by tachycardic palpitations, arrhythmic characteristic syncope, or sudden death. The most prevalent channelopathies are Brugada Syndrome (1:5,000) and Long QT Syndrome (1:5,000). Other rarer diseases are catecholaminergic polymorphic ventricular tachycardia and short QT syndrome.

The estimated cardiovascular risk in this population is very variable and there is no complementary test that adequately stratifies it. The surgical and perioperative risk of these patients is poorly known, but some recommendations are well established by specialists.^162^ In a retrospective analysis of 1,700 cases of early sudden death, 50 occurred postoperatively in young patients with no history of cardiopathy and a part may represent primary arrhythmias.^163^

Symptomatic patients (syncope or palpitation) have a higher risk and sometimes need drugs (quinidine, β-blockers) or pacemaker defibrillators.^164^ Except for urgent surgeries, these patients should be evaluated by cardiologists before surgical release.

Channelopathies present specific anesthetic indications and immediate therapeutic interventions, which make perioperative management of these patients a challenge for the anesthesiologist.^162^ In addition to cardiac monitoring and adequate electrolyte balance, all the drugs used in the perioperative period should be checked on specific sites, such as www.brugadadrugs.org, for Brugada Syndrome, and www.crediblemed.org, mainly for Long QT Syndrome, and also for all other channelopathies.^164^

### F) Conduction Disorders and Provisional Pacemaker Indications

Atrioventricular and intraventricular conduction disorders are uncommon in the perioperative period. When they occur, identification of the cause and drug therapy are usually sufficient for treatment. Even in individuals with bifascicular block or left bundle branch block and first-degree AV block, progression of the block or severe bradycardia in the perioperative period is rare.^165,166^

First-degree AV block, second-degree type Mobitz I, and uni- or bifascicular blocks, particularly in asymptomatic individuals, during the preoperative evaluation represent benign conditions that do not pose a greater risk. On the other hand, individuals presenting syncope, dyspnea or dizziness and type II second degree AV block, advanced AV block, and complete AV block constitute a higher risk group. A more rigorous evaluation is necessary in the preoperative period, and implantation of a pacemaker should be considered. If the surgery is urgent or an emergency, when it is not possible to comply with the ideal time between implantation of the definitive pacemaker and noncardiac surgery, the provisional pacemaker should be implanted in the preoperative period. Indications for implantation of the device under these conditions have already been considered in the Brazilian Guidelines for Implantable Electronic Cardiac Devices.^167^

Indications for a temporary transvenous pacemaker include syncope at rest or hemodynamic impairment due to bradyarrhythmia or occurrence of ventricular tachycardia in response to bradycardia. These recommendations may be based on clinical experience rather than on scientific studies.^168^ In rare occasions, these devices may be electively indicated to assist in procedures that may promote bradycardia or for general anesthesia in patients with second- or third-degree AV block, intermittent AV block, bifascicular block with first-degree AVB, and first-degree AV block with left bundle branch block.^168^

### G) Cardiac Implantable Electronic Devices

Technological progress of artificial cardiac pacing has greatly developed in the last years with the emergence of a wide variety of implantable devices able to provide new interactions with the cardiac rhythm. In addition, an increasing number of patients receive treatment with these new technologies each year. In the USA, approximately 500,000 individuals have these prostheses, and approximately 115,000 new devices are implanted annually. In Brazil, around 25,320 devices are implanted per year (average of the last 5 years) according to the Brazilian Pacemaker Registry.

A concern in the perioperative period of patients with these implantable devices is the possibility of electromagnetic interference when using an electric scalpel and other equipment during surgical procedures.169 Prostheses currently implanted may be functionally simple or have great complexity. We will address conventional pacing (uni or bicameral) (CP), cardiac resynchronizers (CR), implantable cardioverter defibrillator (ICD), and combined prostheses. These prostheses are generally referred to as cardiac implantable electronic devices (CIED).

#### I. Cardiac Implantable Electronic Devices Implanted Less than 60 Days

Most current pacemakers have active fixation electrodes, which enable them to be actively fixed to the endocardium and cardiac veins. Displacement of these electrodes is rare, but it is a possible complication at this stage. The area where the generator is implanted is in the process of surgical recovery. Inflammatory responses, bruises, edema, rejections, and even infections, which could still be subclinical at that stage, can occur with various intensities. The CIED and electrodes are susceptible to infections by organisms originating from other areas and even from surgical manipulations. If possible, it is recommended to wait until the end of the second post-implant month to perform elective noncardiac surgery to minimize the risk of complications.

#### II. Near End-of-life Cardiac Implantable Electronic Devices Battery Depletion

End-of-life CIEDs due to advanced battery wear should be replaced with newer and more modern units before elective noncardiac surgeries. These devices may present adverse behavior when subjected to extreme operating conditions (repeated interrogations and schedules), which may occur during surgery. In addition, these CIEDs can enter the end-of-life mode, changing the behavior and even disabling several important functions to save battery, which could be important in the perioperative period.

#### III. Safe Cardiac Stimulation

For elective noncardiac surgeries, patients should be evaluated by their CIED physicians, which will perform a complete verification of the stimulation system. The physician will determine the need for special programming, report the care that should be taken by the surgeon and anesthesiologist, and describe the possible behaviors of the CIED during surgery or even indicate the need for a stimulus during the procedure to make necessary perioperative schedules, which is usually required in patients at higher risk and with more complex CIED, such as defibrillators.^170^

The biggest concern usually involves those patients scheduled for major surgeries with the use of electric scalpels. In such cases, a safety evaluation must be performed always in a pacemaker evaluation unit and by a qualified physician. The physician should program the pacemaker in asynchronous mode only in cases where the patient depends on pacing and has no arrhythmia history (avoiding competition between pacemaker pace and self-pacing). The physician should also advise the surgical team to use bipolar or ultrasonic scalpel when possible, as these types of devices interfere less with the CIED.

The report should contain at least the recommendations described below for surgeries without the presence of the physician who programmed the pacemaker:

- Continuous cardiac monitoring with an ECG and a pulse oximeter (heart rhythm monitoring is possible even when using an electric scalpel).
- Use a bipolar electric scalpel. If it is not possible, use a unipolar scalpel and place the dispersive electrode (scalpel plate) away from the pacemaker (see below). Subsequently, prepare the skin in the region and eliminate oils by using an alcohol. If the dispersive electrode is reusable, apply a thin and homogeneous layer of electrolytic paste on its surface.
- The dispersive electrode should be placed away from the CIED, preferably close to the surgical region to minimize the electric field. Thus, in an abdominal or pelvic surgery, the dispersive electrode should be placed close to the coccyx; in a right-hand surgery, the dispersive electrode should be placed on the right forearm; and in a head surgery, the dispersive electrode should be placed on the neck (nape). The CIED and its electrodes should be always away from the electric field generated by the electric scalpel.
- Ground the scalpel by connecting it to an efficient ground wire.
- Limit the use of the electric scalpel as much as possible to short and irregular intervals and evaluate the ECG or pulse. During this procedure, the ECG monitor is usually unreadable. Monitoring can be performed using plethysmography, which does not interfere with the electric scalpel.
- If bradycardia or tachycardia occurs while using the electric scalpel (due to electromagnetic interference), place a magnet on the pacemaker every time the electric scalpel is used and then remove it immediately. The magnetic response of each pacemaker may be different and it may not exist in some cases (if it was programmed to be turned off). A good practice is to perform a few tests before surgery, but the patient must be continuously monitored to observe the magnetic response of the device. In addition, the magnetic behavior of each patient’s pacemaker should be reported by the specialist physician, as this depends on the program of the device.^171^ In defibrillators, placing a magnet on the device may turn off antitachycardia therapies, leaving the patient unprotected.
- The patient should be advised to return to the pacemaker assessment clinic after the postoperative recovery period so that the normal set-up of the generator is re-established and the pacemaker functions are re-evaluated.

In patients with resynchronizers, the presence of a greater number of electrodes in the heart undeniably increases the possibility of complications due to external interferences on the stimulation system. Most stimulation electrodes used in the venous system of the left ventricle are used in unipolar mode. Therefore, they are more susceptible to external interferences, particularly those produced by the electric scalpel. However, there is a current trend of using bipolar and even multi-polar electrodes. Notably, many unipolar implants that have already been implanted will remain active for many years. The presence of more electrodes and unipolar electrodes forces physicians to take rigorous measures and pay more attention to signs of interference in the multisite stimulation system.

#### IV. Patients with Implantable Cardioverter Defibrillator

The complexity and diversity of the behavior of ICD, the risk of serious arrhythmias during surgery, and the potential electromagnetic interferences from an electric scalpel with the release of inappropriate shocks lead us to recommend, whenever possible, the presence of the specialist in the operating room with the ICD equipment. The equipment enables to program the ICD during the procedure in accordance to the clinical and metabolic needs of the patient. The antitachycardia function should be turned off, and the patient must be properly monitored. Turning off the function makes the patient unprotected. The physician should be prepared to treat a high-risk arrhythmia by using an external defibrillator and antiarrhythmic drugs. Following the experts’ advice, this type of patient must stay in the ICU during the postoperative period to allow monitoring during the critical stage. At the end of the surgery, the ICD parameters should be re-established and even adjusted to the patient’s clinical condition. The antitachycardia function of the ICD must be switched back on.

#### V. Emergency Electrical Cardioversion or Defibrillation

During the perioperative period, patients with CIED may present complications that require application of electrical cardioversion or defibrillation. Although generators can theoretically withstand shocks, it is advisable to avoid them whenever possible.172 When necessary, some care should be taken to preserve the pacemaker or defibrillator, the electrodes, and the heart-electrode interface, as described below:

- If the patient has an implantable defibrillator, internal cardioversion is recommended because it uses a small amount of energy, biphasic pulse, and internal safety features of the device itself.
- For external shocks, prefer biphasic cardioverters with adhesive pads. Place them in an anteroposterior position (embracing the left ventricle) in accordance to the polarity given by the manufacturer. The classic arrangement of the pads (between the base and tip of the heart - parallel to the electrodes) should be avoided because of the risk of myocardial injury due to contact with the electrode tip.
- Adhere the pads in the chest as far as possible from the generator and the electrodes.
- Use as little energy as possible. Modern biphasic external cardioverters should be preferred, whenever possible, because they use less energy;
- Place a magnet over the pacemaker generator. Older pacemakers invariably switch off when a magnet is placed over them and become asynchronous. In current devices, the magnetic response is programmable and may exhibit different behavior. Therefore, placing a magnet over the generator does not guarantee protection during cardioversion.
- Placing a magnet over the ICD is not recommended because the antitachycardic function can be switched off if it remains over the ICD for more than 30 seconds.
- After the procedure, re-evaluate the sensitivity and command thresholds. Consider reassessing the device in 24 hours, and monitor the patient during this period.

#### VI. Lithotripsy

Shocks generated by lithotripsy have been related to transitory events of loss of sensitivity and command of the CIED, as well as reversion to the safe mode; however, these situations are extremely rare.^173^ When lithotripsy is required in patients with pacemaker and/or defibrillator, direct the focus away from the area of the apparatus and electrodes.

Turn off atrial stimulation when ECG-triggered lithotripsy is used to avoid the device from synchronization with the atrium. Programming the atrial channel with less energy and in the bipolar mode may be another option, keeping the bicameral stimulation more physiological. A test can be performed before the application to observe the behavior and the interaction of the devices. Do not immerse the part of the body that contains the pacemaker or ICD when performing immersion lithotripsy.

It is also recommended to monitor the patient throughout the procedure and, whenever possible, the physician should stay in the room with the CIED programmer to adjust the program of the device as needed. Since lithotripsy is usually outpatient, the patient may perform a regular assessment of the CIED and the specialist can be requested to provide specific guidelines for the procedure. Placing a magnet over the CIED during lithotripsy should not be a rule because, as previously discussed, the device may have different behaviors and may deactivate therapies.

#### VII. Magnetic Resonance Imaging

Magnetic resonance imaging should not be performed on patients with older CIEDs. There is a risk of dysfunction of the prosthesis, electrodes, and even displacement due to the magnetic field generated.^174^

The recent advancement of the CIED industry has led to the production of devices that support the field of resonance, including tests of the chest area with a field strength of up to 1.5 Tesla. In this case, both the CIED and electrodes need to be compatible with this technology. Some patients with new devices but with old electrodes (connected or abandoned) could not undergo magnetic resonance imaging.^175^

Despite the development in this field, even patients with CIED and compatible electrodes require the presence of a physician and a programmer during the test because a specific program is required, which should be deactivated at the end of the procedure. The most appropriate recommendations in these cases are previous evaluation of the patient in a pacemaker evaluation unit and guidance from the specialist on whether the patient should undergo testing. It is important to note that not only the images present artifacts due to the presence of the prosthesis but the patient may also experience local discomfort. This local discomfort is frequently described as burning and palpitations or dizziness related to inhibitions/deflagrations of the CIED.

#### VIII. Radiotherapy

Radiotherapy can be used if the radiation focus is not directed to the CIED. If the device is close to the radiation focus, the area should be covered with a lead screen. If the irradiated site is located exactly in the region of the implant or very close to it and many radiotherapy sessions are necessary, reimplantation of the CIED away from the irradiation site should be evaluated.

Direct radiotherapy on the CIED may cause transient or definitive dysfunction of the device and premature battery wear.^176,177^ Each radiotherapy application may put the CIED in safety mode, even if local protection measures are taken; thus, evaluation of the CIED is required after each radiotherapy session.^178^

Electrodes may also be affected with radiotherapy, especially at the site of contact with the endocardium, which may suffer fibrosis and loss of control. This phenomenon can occur from days to months after radiotherapy. Therefore, particular attention should be given to these patients, especially those that depend on stimulation. Under these conditions, a greater frequency of electronic evaluations (weekly/monthly) should be stipulated after the procedure. CIEDs that enable remote evaluation can facilitate this monitoring.^179^

#### IX. Dental Procedures

Dental procedures in patients with CIEDs are increasingly common in dental practice. In addition to the risk of infection in prostheses, interaction between the equipment used in the dental treatment and the CIED, particularly the electric scalpel, is also possible.^180^ In this situation, care taken should be the same as covered in item 4.G.III for general surgeries.

In patients with ICD, the device may interpret the thermocautery interference as an arrhythmia and release low- or high-energy therapy, placing both the patient and the dental surgeon at risk of receiving an inappropriate shock. Placing a magnet on the generator does not provide adequate protection and should be avoided.

In dental procedures, where the use of thermocautery is mandatory and the patient has ICD, it is essential to disable the antitachycardia therapy, keeping only the pacemaker function.

To prevent untreated risk of arrhythmias in this condition, the patient should be monitored and an external defibrillator should be available on site. The decision to perform the procedure in a hospital environment should always be considered due to the presence of necessary equipment for electrical emergencies, besides the support of the arrhythmologist. General, simple, and routine dentistry procedures carried out in dental practices do not require additional precaution, in addition to the usual therapeutic recommendations. Analgesia should be effective, and the use of anesthetics with vasoconstrictors at recommended doses (vasoconstrictor concentration and amount) should not be avoided. According to case studies and systematic reviews, these anesthetics do not interfere with cardiovascular parameters and do not predispose to coronary events, and when they induce arrhythmias, the risk is low.^181^

#### X. Small Outpatient Surgical Procedures under Local Anesthesia

Minor surgeries may be performed with the usual care for patients with CIED provided that the thermocautery is not used. Analgesia should be efficient in dental treatments, and local anesthetics with vasoconstrictors at the usual doses can be administered to patients with cardiopathies because of the low risk of complications.

The cardiologist should, whenever possible, provide guidance in advance to the person responsible for the surgical procedure. If this is not possible, the use of the thermocautery should be avoided. Placing a magnet over the CIED does not guarantee protection in all cases and is not recommended in all situations.

#### XI. Recommendations

The operative period was divided into preoperative evaluation; preoperative preparation; and intraoperative and postoperative care. The recommendations were grouped to facilitate follow-up of patients with CIED. The suggested sequence should be followed for each patient.

## 5. Interventions and Procedures with Specific Features in the Perioperative Period

### A) Transplants

#### I. Liver

Liver transplantation remains the procedure of choice for the treatment of terminal liver disease. However, changes occurring both intraoperatively and post-transplantation have increased the existing cardiovascular morbidity in these patients. This may be due to several risk factors that affect the population of the same age group (such as age, diabetes, male gender, smoking, previous history of CAD) or it may be related to liver disease and its etiology, such as alcohol-related cardiomyopathy, deposition of amyloid substances, and alterations due to cirrhosis-associated cardiomyopathy.^184^

Studies have shown that cardiac events can occur in up to 70% of post-transplant patients, depending on the criteria.^185^ Among the most common are arrhythmias, pulmonary edema, and systolic ventricular dysfunction, but sudden death and myocardial infarction may also occur.^186^

Therefore, careful investigation of heart disease in candidates for liver transplantation is required.^187^ However, due to liver disease, cardiological investigation in these patients is difficult because the hemodynamic changes and limitations resulting from the disease do not allow the same sensitivity and specificity in cardiology tests, when compared to other populations.^188^

Approximately 50% of patients with cirrhosis show an increase in the QT interval.^189^ β-adrenergic receptors respond only slightly to sympathetic stimuli, leading to dubious responses in studies of dobutamine echocardiography.^190^ The hyperdynamic state and its consequent chronic vasodilatation impair the vasodilator-induced response, such as dipyridamole in myocardial scintigraphy. Terminal liver disease is often accompanied by renal dysfunction, which impairs the use of contrast agents, such as in coronary angiography or coronary Angio-CT.

However, there are tests and approaches that have become routinely used in the preoperative evaluation of liver transplant candidates due to their cost-effectiveness. Moreover, some cardiovascular comorbidity characteristics of cirrhotic patients should be ruled out because they lead to high morbidity and mortality in the perioperative period. The most important characteristics to note are as follows:

#### I. A. Cardiomyopathy Associated with Cirrhosis

It is characterized by the triad: systolic dysfunction, mainly due to a deficit in the contractile response induced by stress, and ejection fraction at rest below 55%; diastolic dysfunction, typically with E/A < 1 and prolonged isovolumetric relaxation time; and electrophysiological changes, particularly increased QT interval, chronotropic deficit and bradycardia, changes in ventricular repolarization, increased left atrium and myocardial mass, and increased levels of BNP, NTpro-BNP, and TnI.^187,188,191^

Although these findings increase morbidity and mortality in liver transplant candidates, no benefit has been demonstrated in the specific treatment of these alterations.

#### I. B. Cardiomyopathy Associated with Alcohol

Cardiomyopathy associated with alcohol accounts 21-32% of patients with dilated cardiomyopathy in some medical centers. Excessive ingestion of alcohol leads to myocyte apoptosis, reduced calcium sensitivity, depressed myocyte contractile function, and myocardial fibrosis.^192^ Considering that alcoholic cirrhosis is among the major causes of liver disease, concomitant occurrence of cirrhosis and dilated cardiomyopathy is relatively common.

Interruption of alcohol intake in the early phase of this cardiomyopathy may lead to partial or total recovery of ventricular function, which reduces cardiovascular morbidity in these patients.^193^

#### I. C. Port-Pulmonary Hypertension

The hyperdynamic state of the patient with portal hypertension may cause vasoconstriction and remodeling of the pulmonary vessels, leading to pulmonary hypertension (PH). These changes affect 5-10% of the transplant candidates and, depending on the pulmonary artery average pressure, may be mild (> 25 and < 35 mmHg), moderate (> 35 and < 45 mmHg), and severe (> 45 mmHg).^194^

Although there are studies on endothelin receptor antagonists and phosphodiesterase inhibitors, there are no definitive guidelines for moderate to severe PH. Perioperative mortality in the latter is close to 100%, therefore representing a contraindication for isolated liver transplantation, and combined lung-liver transplantation may be indicated in specific medical centers.^195^

#### I. D. Hepatopulmonary Syndrome

Although sometimes confused with PH, hepatopulmonary syndrome presents several different characteristics. It is defined as hypoxia in the presence of hepatic disease that worsens with upright posture, with evidence of intrapulmonary vasodilation. Hypoxia is due to the accumulation of pulmonary vasodilators, particularly nitric oxide, leading to intrapulmonary arteriovenous shunt.^196^

Unlike for PH, the selected treatment for hepatopulmonary syndrome is liver transplantation, although non-definitive data correlates the degree of hypoxia with perioperative mortality.^197^

#### I. E. Coronary Artery Disease

As previously mentioned, the risk factors for CAD are either similar or more in cirrhotic patients than in the general population, especially diabetes. Although CAD increases the morbidity and mortality of these patients, the degree of impairment of stenosis does not seem to correlate with worse prognosis.

Computerized coronary tomography with quantification of the calcium score has been recently shown to be useful, and a calcium score above 400 has a high predictive value for early cardiovascular events in these patients.^198^ However, the use of this test cannot be routinely indicated for this population.

The choice of treatment for these patients should consider that lack of intervention may lead to an excessive risk during and after surgery. However, the best treatment is not established and should be customized for each patient.

An important controversy concerns the use of drug eluting stents that requires antiplatelet agents for a longer time in patients with thrombocytopenia, and the risk of bleeding is always present.^199^

Coronary artery bypass grafting (CABG) should be, whenever possible, postponed until after transplantation because of the high probability of hemorrhagic events or worsening of the hepatic condition with the surgery.^200^ Myocardial revascularization before the transplant should be only performed in patients for whom the risk of death due to CAD exceeds the risk of death due to liver disease.

Finally, it is worth mentioning that the cardiologist is part of the multidisciplinary team that accompanies these patients. For this reason, a possible contraindication of the procedure should always be discussed and customized with the multiprofessional team and the patient.

#### II. Kidney

Patients with terminal renal disease are one of the highest cardiovascular risk groups, with mortality rates from cardiovascular disease 5 to 100 times higher than those found in the general population.^201^ Cardiovascular disease is the leading cause of death after renal transplantation, particularly due to CAD.^202^ In the first 30 days after successful kidney transplantation, approximately half of the deaths are due to MI.^203^ Similarly, in the late follow-up, chronic ischemic heart disease accounts for more than a third of deaths in patients with a functioning graft.^204^

Therefore, the preoperative evaluation of renal transplant candidates aims not only to reduce cardiovascular risk in the short term, related to the surgical procedure, but also to reduce cardiovascular events in late follow-up.^205^ During the evaluation of renal transplant candidates, the identification and presence of CAD are of fundamental importance because they allow the medical team to establish more precisely the risk-benefit of transplantation, the possible need for coronary intervention in the preoperative period, the use of cardioprotective measures in the perioperative period, and the control of risk factors in the postoperative period.

This section aims to provide the cardiologist with the most appropriate methods to determine cardiovascular risk in a very special population of patients, almost always excluded from the risk stratification studies. The main aim is to identify those most likely to be diagnosed with CAD among kidney transplant candidates. Thus, the recommendations included in this section should be applied only to asymptomatic patients or patients with atypical symptoms. For those individuals with clinical evidence and/or diagnostic investigations suggestive of coronary disease, further investigation and treatment should follow the proposed rules for the general population.

Identification of relevant CAD is challenging in renal transplant candidates. Patients with terminal renal disease often have atypical symptoms, or are even asymptomatic, in the presence of advanced CAD.^206^ The use of non-invasive methods, such as exercise ECG testing, MPS, and stress echocardiography, all routinely performed in the general population has lower sensitivity and specificity than in individuals with normal renal function, providing numerous false negative results.^207-209^ On the other hand, coronary angiography should not be performed indiscriminately because it is an invasive method, with risk of complications and high cost. In addition, the prevalence of significant CAD in patients routinely assessed invasively is less than 50%.^207,209,210^

Observational studies show that patients with terminal chronic kidney disease and CAD undergoing PCI or CABG have a risk of cardiovascular events similar to those with terminal chronic kidney disease without significant CAD. Those with obstructive coronary disease who did not undergo myocardial revascularization had significantly higher rates of cardiovascular events.^207,208^

Therefore, it is necessary to define a strategy that allows identification of patients more likely to have relevant CAD and who should therefore be referred for angiographic study. With this, we would reduce the number of patients inadequately classified as being of low cardiovascular risk due to failure in the preoperative risk stratification and consequently their exposure to a higher risk of cardiovascular events.

#### Risk Stratification of the Presence of Coronary Artery Disease

The clinical parameters most strongly associated with ischemic heart disease after kidney transplantation are age > 50 years, DM, and previous evidence of cardiovascular disease (clinical history and/or tests).^211^ The prevalence of relevant CAD (stenosis ≥ 70%) increases with the number of risk factors. These three risk factors have been the basis for the formulation of algorithms to investigate coronary disease in patients with chronic kidney disease. Other factors considered as predictors of cardiovascular events in this population are systemic arterial hypertension, left ventricular hypertrophy, smoking, dyslipidemia, and dialysis for more than one year.^212^

Based on the results of existing studies, we proposed a risk stratification model for asymptomatic chronic renal patients from a cardiovascular perspective, evaluated for renal transplantation, according to the presence or absence of the three previously mentioned risk factors.^213-216^ If there is any latency between initial stratification and transplantation, we suggest a period of three years for a new stratification if the patient is stable and without new cardiovascular events or symptoms.

### B) Bariatric Surgery

With the obesity epidemic and the increasing prevalence of type 2 diabetes, bariatric or metabolic surgical interventions have become a very interesting option. The results of long-term (though not randomized) studies seem to indicate the benefit of these interventions in reducing mortality. However, there are still many uncertainties, in particular, which patient profile may benefit and what type of surgical intervention to perform for each case. Once the bariatric surgical procedure is considered, it is important to pay attention to the contraindications for this type of surgery: type 1 diabetes, drug or alcohol abuse, uncontrolled psychiatric disease, lack of understanding about risks, alternatives, and complications of the intervention, and lack of commitment to the need for nutritional supplementation and clinical follow-up.

In Brazil, four different types of bariatric surgery (besides the intragastric balloon, which is not considered surgical) are approved: gastric bypass, adjustable gastric band, duodenal switch, and vertical gastrectomy. However, there are no conclusive data to support the selection of procedure based, for example, on the higher or lower incidence of complications. On the other hand, several variables, such as age, gender, BMI, presence of comorbidities, and the patient’s desire, should be considered. Thus, for example, the presence of important hiatal hernia contraindicates vertical gastrectomy, the patient with a very high BMI cannot receive an adjustable gastric banding, vertical gastrectomy is less efficient in a patient who continually eats, or gastric bypass will probably work better if the patient is a long-term diabetic.

Regarding the perioperative evaluation for patients with indication for bariatric surgery, in addition to the general recommendations described for obese patients in another item of this guideline (item 9 D), there are some specific considerations that consider studies that observed the occurrence of complications. DeMaria et al. evaluated 2,075 patients undergoing bariatric surgery (all undergoing gastric bypass) using the obesity surgery mortality risk stratification score and found increased risk of death in the presence of certain factors: pulmonary thromboembolism (PTE) or risk for PTE, BMI > 50 kg/m^2^, male gender, systemic arterial hypertension, and > 45 years of age. The risk for PTE was defined as previous PTE, presence of a vena cava filter, right HF and/or PH, chronic venous stasis, and obstructive sleep apnea syndrome. Basing on these data, the authors developed a risk score based on the number of risk factors: A (0-1), B (2-3), and C (4-5), which correspond to an estimated perioperative mortality risk of 0.31, 1.90, and 7.56%, respectively.^217^ Patients undergoing bariatric surgery in the National Surgical Quality Improvement Program (NSQIP) of the USA were evaluated. Tools were developed and validated for mortality^218^ and morbidity^219^ risk, specifically for this type of intervention, and this can be accessed and used online:

http://www.surgicalriskcalculator.com/bariatric-surgery-risk-calculator

The largest prospective study performed to date is the longitudinal assessment of bariatric surgery (LABS), with results published in July 2009. A total of 4,776 bariatric surgical interventions were analyzed, and lower complication rates were observed, which is not in agreement with the findings of DeMaria et al.^217^ The authors found a general mortality rate of 0.3% in a period of 30 days and an outcome composed of death, deep venous thrombosis (DVT), PTE, reintervention, and hospitalization > 30 days in 4.3% of the patients. Some predictors of the outcome were similar to those found by DeMaria,^217^ such as BMI > 70 kg/m^2^, DVT or prior PTE (8.8% of events), and sleep apnea (5.0% of events). The authors also found a correlation between the outcome and diabetes (5.5% of events), and type of surgery and low functional capacity (inability to walk more than 61 meters without dyspnea with 15.9% of events). In this study, the type of surgery with the best outcome was laparoscopic gastric banding (1.0%) compared to laparoscopic Roux-en-Y gastric bypass (4.8%) and open surgery Roux-en-Y gastric bypass (7.8%).^220^

Another study with more than 91,000 patients observed that venous thromboembolism occurred in the first 30 days postoperatively in 0.29% of the cases of obese patients undergoing bariatric or metabolic surgery. However, more than 80% of the cases of thromboembolism occurred after hospital discharge. HF, paraplegia, dyspnea at rest, and resurgery were associated with a higher risk of thromboembolism. The authors suggested that routine pharmacological thromboprophylaxis should be considered for high-risk patients (> 0.4%).^221^

However, there is no consensus with regard to the most appropriate prophylactic measure. A recent meta-analysis found no benefit for any of the different strategies, with enoxaparion ranging from 40 mg per day to 60 mg twice a day.^222^

On the other hand, a study analyzed the strategy of dividing the groups of patients submitted to bariatric surgery (93% gastric bypass) according to the BMI, administering 40 mg twice a day to the group with BMI lower than or equal to 50 kg/m^2^ and 60 mg twice a day to the group with BMI > 50 kg/m^2^. The study reported that 74% of the patients reached anti-Xa therapeutic levels and only 1.79% needed blood transfusion.^223^

In addition to the recommendations for obese patients described in another item in this guideline (item 9D), the following recommendations are added for patients with indication for surgical intervention:

### C) Arterial Vascular Surgeries

Arterial vascular surgeries represent the group of interventions associated with a higher incidence of cardiovascular complications, with rates of almost 50% in some cases, which questions the validity of performing the procedure.^224^

On the other hand, it is important to know the indications based on evidences that showed favorable risk-benefit ratio and to identify all variables involved in risk estimation of this type of intervention. This is discussed in more detail in the updated version of the II Guideline for Perioperative Evaluation, with a focus on arterial vascular surgeries, which can be accessed using the link:^50,225^

http://publicacoes.cardiol.br/consenso/2013/II_Diretriz_de_Avalia%C3%A7%C3%A3o_Perioperat%C3%B3ria.asp.

The recommendations and general care in this guideline are also necessary for this specific population; however, there are additional specific issues that are addressed below.^226,227^

### D) Low-risk Procedures

#### I. Dental

The preparation of dental procedures in patients with heart disease is not based solely on the use of antibiotic prophylaxis, vasoconstrictors, and/or control of postoperative bleeding. The presence of infectious foci in the oral cavity may represent a factor of postoperative complication. The incidence of odontogenic bacteremia increases significantly in the presence of infectious foci, such as in periodontal disease and endodontic lesions.

Although the occurrence of bacteremias is commonly reported during dental procedures, they occur with similar frequency during oral hygiene and chewing.^228^ For this reason, assessment of oral health with elimination of infectious foci and intensive oral hygiene control of hospitalized patients is advisable whenever possible prior to surgical procedures in patients with or without heart disease to reduce perioperative complications.

In general, patients with controlled heart disease under optimized medication can undergo a dental procedure safely with the usual routine care.

Individuals with pacemakers and implantable automatic defibrillators do not present changes with high or low rotation motors, amalgamators, electric pulp tests, electric toothbrushes, endodontic ultrasound, periodontal ultrasound, and radiography. The use of an electric scalpel has specific guidelines discussed in this guideline (item 4.G.III). Further studies are needed to determine the possible effect of the laser on pacemakers.

#### I. A. Local Anesthetics: to Use or Not to Use Local Vasoconstrictors

The use of local anesthetics with vasoconstrictors in patients with heart disease has generated controversy. The administration of vasoconstrictors in combination with local anesthetics increases the quality and duration of pain control and promotes reduction of bleeding.^229^ Local anesthetic without vasoconstrictor has a short duration, rapid absorption (high toxic potential), and inadequate pain control. It can also generate hemodynamic changes and even cardiac arrhythmias, besides promoting mild vasodilation, increasing bleeding.

Lidocaine with epinephrine has been the most widely used local anesthetic worldwide. Although the interaction of epinephrine with β-blockers, tricyclic antidepressants, diuretics, and cocaine has been reported in the literature, the use of two to three 2.0% lidocaine tubes with 1:100,000 epinephrine (36-54 µg epinephrine) is well tolerated in most patients. This also applies to individuals with hypertension or other cardiovascular disease, situations where the use of this vasoconstrictor has more benefits than risks.^229^

#### I. B. Patients Using Antithrombotic Agents (Antiplatelet Agents and Oral Anticoagulants)

Most dental procedures have low risk of bleeding. Therefore, warfarin should not be discontinued in most patients submitted to dental procedures, including dental extractions.^230^ A meta-analysis^231^ and smaller studies^232-234^ have demonstrated safety in performing dental procedures in anticoagulated patients with international normalized ratio (INR) < 4.0 by using local measures to reduce bleeding. Major procedures, and consequently with greater risk of bleeding, such as extraction > 3 teeth, should have customized discussion based on the thrombotic risk of each patient to determine interruption of therapy and possible bridge therapy, as discussed in specific section in this guideline.

To date, evidence of bleeding risk in patients using new anticoagulants (NOACs) in dental procedures is limited.^235^ There are also no recommendations available for perioperative measures.

Regarding antiplatelet agents, several studies in the literature show the safety of performing dental procedures usually using aspirin or clopidogrel monotherapy.^236-239^ Although bleeding increases, this is easily controlled with local hemostatic measures.^240-242^ Therefore, patients in secondary prevention of cardiovascular events using aspirin or clopidogrel monotherapy should keep using the drugs in the perioperative period of the procedures.

Patients on DAPT with a recent PCI (6 weeks after bare metal stent (BMS) and 6 months after drug eluting stent - DES) or acute coronary syndrome for less than one year should maintain the use of the drugs, if dental procedures are required in the period of highest risk of intra-stent thrombosis. There is already evidence in the literature on the safety of this strategy when local hemostatic measures are increased. Studies with ticagrelor or prasugrel remain scarce,^243^ but the recommendation is to maintain them in these conditions of DAPT in periods of increased risk of intra-stent thrombosis.

When using antithrombotic therapy, dental procedures may be performed following some precautions.

#### Specific considerations that may be suggested to dentists

Some precautions and measures may be considered to reduce bleeding in patients taking antithrombotic drugs.

#### I. C. Use of Antibiotics with Anticoagulants

The use of antibiotics for endocarditis prophylaxis is indicated in patients with previous history of endocarditis or valve disease who will undergo procedures involving manipulation of gingival tissue, periodontal region, or perforation of the oral mucosa, as discussed in a specific section in this guideline (item 7.E.I). Antibiotics often used for this purpose may interfere with the metabolism of oral anticoagulants, particularly warfarin. Patients taking anticoagulants should be alerted to a possible increase in bleeding and control the INR, if necessary. There is no need to change the anticoagulant regimen when a single dose of prophylactic antibiotic is used.

#### II. Dermatological

Dermatological surgical procedures are low-risk procedures for both cardiovascular and bleeding events. Data from the literature suggest that approximately 50% of patients scheduled for dermatological procedures are using antiplatelet or anticoagulant therapy.^244,245^ In these cases, the surgical team and the anesthesiologist should be informed about the drugs used and the necessary care, including a more time-consuming and cautious hemostasis, because in most cases the risk associated with discontinuation of antithrombotic therapy outweighs the risk of bleeding inherent in the procedure.

Suspension of the use of aspirin for secondary prevention of cardiovascular events is not necessary before performing any dermatological surgical intervention.^246,247^ For patients using double antiplatelet therapy for stent implantation who are not in the period of greatest thrombotic risk, the recommendation is to suspend the second antiplatelet drug,^248,249^ considering the intervals already described in this guideline (see section on antiplatelet agents - item 7.A.V).

For individuals taking warfarin, the recommendation is to continue its use and adjust the INR to ≤ 3.5 to minimize the risk of bleeding.^248^ However, some studies have not demonstrated the correlation between INR levels and the risk of increased bleeding in patients taking warfarin.^250^ Although evidence is scarce, it is recommended that patients taking one of the new oral anticoagulants (NOACs) can perform most of the dermatological procedures during the medications.^248^ This is to ensure that the surgical intervention is scheduled, whenever possible, a few hours before the next dose to avoid the peak serum level of the drug.

#### III. Endoscopic

Considering the risk analysis of cardiovascular events, endoscopic procedures are low risk.^251^ Thus, suspension of the procedure is not required for cardiovascular intervention, except in severe cardiovascular conditions already mentioned in the section of perioperative evaluation algorithms of this guideline. In addition, most drugs that are included in the cardiovascular therapy do not need to be discontinued and can be ingested with minimal water. In fact, the most important issue is whether the patient makes use of antithrombotic drugs due to the potential risk of endoscopic bleeding and thromboembolic events caused by discontinuation of these drugs.

Endoscopic procedures have different bleeding potentials, which is very important to determine the strategy to be used. The risk varies with the type of procedure and is mainly related to the existence of therapeutic interventions. Chart 4 presents the risks of bleeding attributed to common endoscopic procedures in clinical practice.^252^ The risk of thromboembolic events with discontinuation of antithrombotic therapy varies with the therapy proposed and individual patient conditions.

**Chart 4:** Bleeding risk in endoscopic procedures*

High-risk procedures	Low-risk procedures
Polypectomy	Diagnostics (UDE, colonoscopy, flexible sigmoidoscopy), including mucosal biopsy
Biliary or pancreatic sphincterotomy	ERCP with stent placement or balloon dilatation without sphincterotomy
Balloon-assisted therapeutic enteroscopy	Balloon-assisted diagnostic enteroscopy and push enteroscopy
Endoscopic percutaneous gastrostomy or jejunostomy	Endoscopic capsule
Endoscopic ultrasonography with fine needle biopsy	Endoscopic ultrasonography without fine needle biopsy
Cystogastrostomy	Placing intestinal stent
Esophageal dilatation	Barrett's esophagus ablation
Mucosectomy and submucosal dissection	Coagulation with argon plasma
Ablation of tumors	

* Adapted from Acosta RD et al.^252^ UDE: upper digestive endoscopy; ERCP: endoscopic retrograde cholangiopancreatography.

#### Management of Antiplatelet Agents in Endoscopic Procedures

For endoscopic procedures classified as low risk of bleeding, antiplatelet therapy may be maintained, either in the form of monotherapy (independent of the agent) or DAPT.^252-255^ For procedures considered as high risk of bleeding, some points should be considered.

Patients taking DAPT for a recent PCI (6 weeks after BMS stent and 6 months after DES) or acute coronary syndrome in the past year present the highest risk if antiplatelet therapy is discontinued. Therefore, elective high-risk bleeding endoscopic procedures should be postponed, whenever possible, until the end of this period of increased risk. However, for procedures that require to be performed during this period, the most accepted strategy is to maintain aspirin and withdraw the second antiplatelet drug,^255,256^ although evidence for this strategy is limited.

Patients taking aspirin monotherapy for secondary prevention of cardiovascular events may maintain the treatment in the perioperative period of endoscopic procedures, even in those considered to be high risk for bleeding, because most evidence in the literature shows a low risk of significant bleeding in these situations.^257-266^ Some studies have demonstrated increased bleeding in procedures, such as submucosal dissection in patients with gastric neoplasia^267^ and mucosectomy in colonic tumors larger than 20 mm,^268^ these procedures should be analyzed individually according to the risk of thrombotic events with suspension of aspirin.^255^ There is some evidence showing the safety of clopidogrel monotherapy during percutaneous endoscopic gastrostomy, and its maintenance may be considered in this situation.^258^ Evidence for the use of prasugrel and ticagrelor in high-risk bleeding endoscopic procedures is scarce.

If antiplatelet therapy is discontinued, the intervals between the suspension and the procedure should follow the recommendations of this guideline in the antiplatelet management section (item 7.A.V.C). Antiplatelet therapy may be resumed after the procedure as soon as hemostasis is achieved. An attack dose may be considered in patients who are at high risk for cardiovascular events.^256^

#### Management of Anticoagulants in Endoscopic Procedures

For endoscopic procedures classified as low risk of bleeding, anticoagulant therapy with warfarin may be maintained^252,253,255,269^ and should be discontinued in those considered to be at high risk of bleeding.^260,266^ To date, no evidence has been reported regarding the use of new anticoagulants (NOACs) in these situations, suggesting that they should be maintained in low-risk bleeding procedures and suspended in those with high risk for bleeding.^252^

Intervals for the suspension and resumption of NOACs and warfarin (including consideration of bridge therapy in those patients considered to be at a greatest risk for thromboembolic events) should follow the guidelines in the perioperative management section of this guideline (section 7.D).

#### IV. Ophthalmologic

Ophthalmologic surgical interventions are relatively frequent procedures in the elderly population. The presence of cardiovascular comorbidities that require the use of antithrombotic drugs and their associated treatment during the perioperative period is a subject of intense debate between ophthalmological surgeons and cardiologists. In Brazil, the fear of hemorrhagic complications, including bruising in the periorbital region, is responsible for the indiscriminate interruption of aspirin and warfarin in 82.7% of patients who undergo glaucoma surgeries.^270^

The limited evidence regarding the occurrence of complications shows that this fear is not reasonable. The rate of hemorrhagic complications described in observational studies is low and without major consequences, particularly in cataract surgeries using conventional anesthetic techniques.^271-275^

Some ophthalmologic surgical interventions, such as trabeculectomy^276,277^ and vitrectomy,^278,279^ which are used to treat glaucoma and retinal diseases, respectively, present a greater hemorrhagic risk. Nevertheless, the evidence does not demonstrate an increased risk of significant hemorrhagic complications in these surgeries with the use of aspirin.^277,280,281^ In such cases, the decision should be customized, but maintaining this agent is generally recommended in the perioperative period.^282^

Patients receiving a DAPT for a recent PCI (6 weeks after BMS stent and 6 months after DES) or acute coronary syndrome in the past year are those at highest risk of events due to discontinuation of antiplatelet therapy. Therefore, ophthalmologic procedures, whenever possible, should be postponed until this period of greatest risk ends.

For procedures that required to be performed in this period, the strategy depends on the hemorrhagic risk of the intervention. For interventions with low hemorrhagic risk (intravitreal injections, cataract, and peribulbar anesthesia), aspirin and P2Y12 receptor inhibitors should be maintained. For interventions with higher hemorrhagic risk, such as vitrectomy and trabulectomy, the most accepted recommendation is the maintenance of aspirin and suspension of the second antiplatelet, considering the intervals already described in a specific section of this guideline (item 7.A.V.C). However, the evidence for this strategy is limited.

Similar to patients taking ASA monotherapy, evidence in the literature favors the maintenance of clopidogrel monotherapy in the perioperative period of cataract surgeries.^274,275^ Evidence is more limited in glaucoma and retinal surgeries. The recommendation is to suspend clopidogrel in the perioperative period of these interventions, considering the 5-day period between the suspension and the procedure.

With regard to patients taking warfarin, the evidence in the literature favors their maintenance in surgeries with lower hemorrhagic risk, such as cataract surgeries, ensuring that the INR is in the therapeutic range.^271,272^ A meta-analysis of observational studies including patients submitted to cataract surgery and using warfarin found a bleeding incidence of around 10%. This incidence was mostly self-limiting, subconjunctival, and with no visual loss.^273^ On the contrary, in glaucoma and retinal disease surgeries, warfarin should be discontinued. Perioperative management should follow the strategy described in this guideline in the perioperative anticoagulation management section (item 7.D), considering the individual risk of thrombotic events of the patients.

To date, evidence of risk of bleeding during ophthalmologic surgeries in patients using new anticoagulants (NOACs) is limited. No recommendations have been established for their perioperative management.

Recommendations, particularly for patients with coronary stents and mechanical valvular prostheses, should be customized, considering the relationship between thrombotic and hemorrhagic risks. For patients recommended to maintain anticoagulant and/or antiplatelet agents, the surgeon should be informed of the need to ensure adequate hemostasis. A suggestion that can be considered and discussed with the anesthesiologist, who makes the final decision, is the use of a specific type of anesthesia that is less associated with hemorrhagic complications.^275^ For antiplatelets, if the decision is to interrupt the drug, it should be restarted postoperatively as soon as possible. In addition, the procedure must be performed in a hospital with competence for urgent hemodynamic intervention (PCI), if necessary.

## 6. Considerations for High-Risk Patients

### A) When the Cardiovascular Risk is Very High - to Perform Surgery or Not to Perform Surgery?

After the patient, the surgeon is the most interested person to define whether a surgery should be performed based on the balance between the risk of complications and the benefit of the intervention. The surgeon generally does not perform surgery if there is a high risk of surgical complications, which is sometimes against the expectations of the patient and his family.^283^

However, there are situations in which perioperative evaluation concludes that the risk of cardiaovascular complications, such as myocardial infarction and stroke is high. In this case, it is important that the cardiologist knows the prognosis of the underlying disease to determine whether the risk-benefit ratio is unfavorable and whether the intervention should not be performed. Such information regarding the prognosis of the underlying disease should be requested to the surgeon who requested the evaluation. **Class of recommendation I, Level of evidence C**.

Noncardiac surgery should be contraindicated when there is objective information that the risk of serious cardiovascular complications, such as cardiac death, nonfatal infarction, and stroke, does not exceed the risk of death from the underlying disease. **Class of recommendation IIa, Level of evidence C**.

### B) Hospital Choice

An important part of the perioperative evaluation by the cardiologist is the analysis of the health institute where the surgical procedure will be performed. Studies have demonstrated that a hospital with a cohesive multiprofessional team who focuses on prompt diagnosis and therapeutics of the complications has a positive influence on the perioperative results.^284-287^

In addition, there is evidence that hospitals with a higher number of procedures have lower perioperative mortality compared with those with fewer procedures, even after adjusting for other variables.^288^

In conclusion, in the evaluation of surgical risk, it is imperative to analyze the variables related to the health institute where the procedure will be performed. The analysis provides our patients with more comprehensive counseling. **Class of recommendation I, Level of evidence C**.

## 7. Measures to Reduce Surgical Risk from a Cardiovascular Perspective

### A) Perioperative Drug Therapy

#### I. β-blockers

The recommendations regarding the use of β-blockers in the perioperative period of noncardiac surgical interventions have been under intense debate in recent years because of the results of large clinical studies. These studies presented limitations that generate discussions in the academic and care communities.

Pioneering studies in the 1990s suggested that perioperative use of β-blockers could reduce cardiovascular mortality and morbidity in a broad spectrum of patients. Three randomized trials conducted between 2005 and 2006 did not confirm the protective effect of β-blockers in the vascular perioperative period of low- or intermediate-risk patients, highlighting the potential harm, given the association with a higher incidence of bradycardia and hypotension.^289-291^

The benefit of β-blockers was later questioned in meta-analyses.^292,293^ On the other hand, the largest retrospective study on the use of β-blockers in the perioperative period, which analyzed more than 780,000 patients submitted to noncardiac surgery, showed that the impact of β-blockers depends on the estimation of cardiac risk. In high-risk patients, β-blockers are associated with lower mortality, whereas in low-risk patients, no benefit was found and the β-blockers could be harmful.^294^ In 2008, the POISE study was conducted. In this study, 8,351 patients who were mostly having an intermediate risk of complications were randomized to receive metoprolol succinate or placebo starting 2-4 hours prior to noncardiac surgery, with doses up to 400 mg in the first 24 hours. The results showed a lower incidence of MI, reversed cardiac arrest, and cardiac mortality in the group with β-blockers. However, the authors observed two times higher incidence of stroke and greater overall mortality in this group. The high incidence of hypotension and bradycardia was strongly associated with higher mortality and stroke.^295^

Careful analysis of these data shows a great heterogeneity among studies, mainly regarding the dosage of β-blockers used and time of onset. There are studies that initiated β-blockers a few hours before the surgery, with no time to determine the doses conferring adequate heart rate control.^289-291^ In other studies, some patients continued to receive β-blockers despite the occurrence of bradycardia and/or hypotension and most importantly without time for hemodynamic adaptation.^295^

On the other hand, there are studies that started β-blockers earlier, at least one week before the surgery, to determine the adequate dose.^296^ These were the studies that showed benefit. In 2008, even before the publication of the POISE study, an interesting publication reviewed data from the two main meta-analyses previously cited^292,293^ based on the heart rate control obtained for each study. The authors divided the data into two groups according to degree of heart rate control, and observed that the trials in which patients achieve the most effective control of heart rate were associated with a reduced incidence of postoperative MI, suggesting that effective control of heart rate is important for achieving cardioprotection.^297^

Thus, once the specific indications have been evaluated, the use of β-blockers in the perioperative period must always comply with safety principles. The time of onset should be as early as possible (at least one week before the surgery) to ensure adequate time to evaluate the hemodynamic response of each patient, avoiding bradycardia and hypotension. Low doses should be prescribed, with progressive titration to a HR of 55 to 65 bpm, without hypotension (SBP > 100 mmHg). During the perioperative period, frequent monitoring of HR and BP must be done. If HR < 50 bpm or SBP < 100 mmHg is detected, the β-blockers should be suspended until hemodynamic and chronotropic balance is restored. On the other hand, from the point of view of effectiveness, the benefit of the β-blockers is associated with adequate heart rate control. Therefore, we should target for a HR of 55 to 65 bpm in the pre- and postoperative periods.

Finally, β-blockers should not be withdraw in the perioperative period of patients who receive them chronically for various indications. Acute β-blocker suspension is associated with a significant increase in postoperative mortality.^298^

#### II. Statins

In addition to reducing cholesterol levels, statins have a pleiotropic effect of reducing inflammation and stabilizing plaques of atherosclerosis. The use of statins to prevent cardiovascular events after vascular surgeries is well established and is based on prospective, randomized, placebo-controlled studies.

In 2004, in the first randomized study published, the authors demonstrated that the use of 20 mg of atorvastatin is associated with a large decrease in major cardiovascular events (death, MI, stroke, unstable angina) in the perioperative period and after 6 months of follow-up. This effect occurred regardless of baseline cholesterol levels.^299^

In 2009, the use of 80 mg of slow-release fluvastatin in 250 patients submitted to vascular surgeries was shown to reduce the occurrence of postoperative myocardial ischemia and the combined outcome of MI and cardiac death in a period of 30 days compared to the placebo group (247 patients).^300^

This result was confirmed in a recent meta-analysis involving 23,536 patients, in which the use of statins in the perioperative vascular period reduces overall mortality and MI and stroke rates.^301^ The specific benefits of statins for each type of vascular procedure can be found in a specific guideline for vascular surgeries.^50^ The administration of 20 mg of atorvastatin (or 40 mg of simvastatin) in patients submitted to vascular surgery should be preferably performed two weeks before the procedure and maintained for 30 days. Subsequently, the dose should be adjusted to the individual LDL goal of each patient.

On the other hand, evidence on the use of statins for the prevention of cardiovascular complications in nonvascular surgeries is obtained from retrospective studies. Lindenauer et al.^302^ evaluated 780,591 patients submitted to noncardiac surgeries (92% nonvascular surgeries) in a retrospective cohort study with 77,082 patients (9.9%) receiving statins. In this study, the patients who received statins had lower mortality during hospital stays. Another retrospective case-control study with only nonvascular surgeries, including 989 patients who died postoperatively within 30 days and 1879 controls, showed that the use of statins is also associated with a reduction in mortality (OR = 0.4; CI 0.24-0.68).^303^ In a retrospective cohort that included 752 patients submitted to nonvascular surgeries, the authors demonstrated a reduction in the combined outcome of nonfatal MI, AF, and mortality in a period of 30 days in patients using statins.^304^ Recently, in an analysis of the patients included in the VISION study,^305^ Berwanger et al.^306^ evaluated 2,842 patients receiving statins and 4,492 patients without statins. They compared the occurrence of mortality, isolated elevation of troponin levels (defined as troponin increased levels, without MI or other cause), and stroke in a period of 30 days by using propensity score matching. About 10% of the patients were submitted to vascular surgeries and the rest to nonvascular procedures. Patients receiving statins showed reduction in the risk of the combined outcome (RR 0.83; 95% CI 0.73-0.95; p = 0.007). The use of statins reduced overall mortality (RR 0.58; 95% CI 0.40-0.83; p = 0.003), cardiovascular mortality (RR 0.42, 95% CI 0.23-0.76; p = 0.004), and the occurrence of isolated increase of troponin levels (RR 0.86, 95% CI 0.73-0.98; p = 0.02). There was no reduction in noncardiovascular mortality and in the rate of MI or stroke. It should be noted that, despite propensity score matching, the patients in the statin group had CAD, diabetes, peripheral vascular disease, aspirin use, and ACE inhibitors/angiotensin receptor blockers more frequently than the patients in the group without statins. Although presenting more risk factors, the patients in the statin group had fewer cardiovascular events.^306^ Basing on these studies, we can conclude that the patients with higher cardiovascular risk and those using statins due to comorbidities (CAD, diabetes, peripheral vascular disease) may benefit with the administration of statins in perioperative nonvascular surgeries.

Statins are often withdraw postoperatively. The main reasons for statin withdrawal are as follows: postoperative ileus and inability to administer oral medications, hemodynamic instability, concern with the occurrence of side effects, and lack of awareness of the importance of maintaining statins.^307^

Perioperative statin suspension in patients who use this medication on a chronic basis is an independent predictor of cardiovascular events following vascular surgeries.^308,309^ The use of statins in the perioperative period is safe. Although patients using statins have a higher baseline CPK level, the occurrence of increases above 5 times their reference value or rhabdomyolysis is rare.^310^ Therefore, in patients who already use statins, it should be maintained in the perioperative period.

#### III. Alpha-agonists

Alpha2-agonists modulate the response of catecholamines to surgery and anesthesia, decreasing the release of noradrenaline and reducing blood pressure and heart rate. The first randomized studies that used clonidine to prevent cardiovascular complications following noncardiac surgeries demonstrated a reduction in myocardial ischemia, but without a reduction in clinical events or mortality.^311,312^ On the other hand, a meta-analysis demonstrated that α2-agonists reduce mortality and MI in patients submitted to vascular surgeries, but not in those submitted to nonvascular surgeries.^313^

The European Mivazerol Trial (EMIT) evaluated the use of mivazerol in 1,897 patients with CAD submitted to noncardiac surgeries. The authors found a decrease in general mortality and MI or cardiac death only in the subgroup of patients submitted to vascular surgeries.^314^

A randomized study with 190 patients demonstrated reductions in myocardial ischemia and mortality with the use of perioperative prophylactic clonidine in patients with CAD or risk factors for CAD,^315^ but these results were not confirmed.

Recently, the POISE-2 study included 10,010 patients submitted to noncardiac surgery in 23 countries. The patients were randomized to receive clonidine or placebo in the perioperative period of noncardiac surgeries. The use of clonidine did not reduce the incidence of death or MI in a period of 30 days (HR 1.08, 95% CI 0.93-1.26; p = 0.29). Furthermore, patients on clonidine more frequently presented clinically significant hypotension (HR 1.3, 95% CI 1.24-1.4, p < 0.001) and reversed cardiac arrest (HR 3.2, 95% CI 1.17-8.76, p = 0.02).^316^

Therefore, the introduction of clonidine in the preoperative period is not recommended to reduce the risk of cardiovascular events.

#### IV. Calcium Channel Blockers

Evidence for the use of calcium channel blockers with the aim to reduce cardiovascular risk in the perioperative period of noncardiac surgeries is scarce. In a meta-analysis of 11 studies involving 1,007 patients, there was no reduction in mortality or MI with verapamil, diltiazem, or dihydropyridine.^317^ Another study evaluated 1,000 patients submitted to aortic aneurysm surgeries, and the results demonstrated an increase in perioperative mortality with the use of calcium channel blockers.^318^

Therefore, the use of calcium channel blockers to prevent cardiovascular events in the perioperative period of noncardiac surgeries is not recommended.

#### V. Antiplatelet Agents

Operating patients who use antiplatelet therapy implies an increased risk of bleeding;^247^ however, the suspension is known to be associated with rebound effect^319^ and clinical atherothrombotic events.^247,320^ In general, the decision should be based on the discussion between the surgical, clinical, and anesthetic teams. The team should consider the risk of exacerbation of bleeding inherent to the surgical procedure and, on the other hand, the thrombotic burden that led to the prescription of the antiplatelet agent.

#### V. A. Acetylsalicylic Acid

POISE-2 study, which was published in 2014, is the largest randomized, placebo-controlled study evaluating the impact of ASA in the perioperative period.^321^ In this study, 10,010 patients with risk factors for perioperative complications receiving ASA or placebo were evaluated. Patients who never took ASA were included, as well as patients who were already on chronic use, randomized to placebo, or continued ASA at the study doses of 200 mg immediately prior to surgery and 100 mg daily for 30 days. The authors did not show a significant difference in the primary outcome (death or MI) or in the secondary outcome of the study (death, MI or stroke). No difference was observed based on the history of use of pre-randomization ASA. On the other hand, they observed a higher incidence of bleeding in the ASA group: 4.6% × 3.8%, p = 0.04, especially at the surgical site.

Some considerations should be made regarding the clinical profile of patients who mostly (almost 70% of the population) had no history of cardiovascular disease and used ASA for primary prevention. Another extremely relevant fact is the non-inclusion of patients with PCI with DES in the last year or BMS in the last 6 weeks. Most surgeries in the study were orthopedic, general, or gynecological, with 605 vascular procedures, for which the main results of the study are maintained.^321^ Thus, the most practical applicability of POISE-2 results is to recommend the non-use of ASA in the perioperative period of individuals in primary prevention. For patients who are already using ASA for primary prevention and are scheduled for noncardiac surgery, suspension of antiplatelet treatment 7 days before is recommended.

Oscarsson et al.^322^ conducted a much smaller study than POISE-2. It included 210 patients. The study design was interesting because it did not investigate the initiation of ASA, but the suspension or maintenance in the noncardiac perioperative period of patients chronically using ASA. Patients scheduled for vascular surgeries were not included (the authors thought that it was unethical to withdraw the antiplatelet treatment in the vascular perioperative period). They observed a lower incidence of cardiovascular events in the group that maintained ASA, without a higher incidence of bleeding. Anecdotally, the subjective notion of the surgeon on the bleeding tendency due to impaired hemostasis during the surgery did not allow discriminating patients receiving placebo or antiplatelets.^322^

In the STRATAGEM study, the patients using ASA only for secondary cardiovascular prevention were randomized to receive 75 mg of ASA or placebo in the perioperative period. The results showed no increased incidence of bleeding or significant difference in thrombotic complications.^323^ However, this study included only 20% of the planned patients, which hinders definitive conclusions for patients at higher risk.

In the vascular perioperative period, evidence suggests the beneficial use of ASA for protection of infrainguinal grafts, but without conclusion about systemic outcomes. On the other hand, Calderaro et al.^324^ analyzed patients in the elective vascular perioperative who were already on chronic use of ASA and observed that those individuals with lower platelet responsivity up to 100 mg daily (according to the aggregability test after stimulation with arachidonic acid) presented more than twice the systemic atherothrombotic events, when compared to the more responsive individuals, without an increase in bleeding rate.^324^

For patients receiving ASA for secondary prevention, it is recommended to maintain it at a maximum dose of 100 mg daily. Meta-analysis data suggest that this ratio is favorable for most perioperative patients.^247^ Neurosurgeries due to high morbidity and mortality associated with bleeding, even small ones, represent an absolute indication for ASA suspension 7 days before.^247^

Patients scheduled for transurethral resection of the prostate using the conventional technique should also suspend ASA owing to the high risk of bleeding.^247^ Urologists recently acknowledged that ASA can be maintained using the hemostatic technique called laser green-light in patients scheduled for transurethral resection.^325,326^ This example demonstrates the benefit of new techniques for more complex patients and the constant need for a multidisciplinary approach to the perioperative decision process. At present, there is no recommendation for routine ASA withdrawal for transrectal prostate biopsy, an extremely common urological procedure.^325^

There is no recommendation to start ASA before noncardiac surgeries. If we evaluate patients with established vascular disease but who erroneously omit using antiplatelets, it is the opinion of this guideline by consensus of the specialists that this therapy should be implemented at the time of hospital discharge. However, no study has supported the administration of the drug before surgery.

#### V. B. Dual Antiplatelet Therapy

Approximately 20% of patients submitted to PCI will require noncardiac surgery in the subsequent 2 years.^327,328^ This implies perioperative management not only of ASA but also of the second antiplatelet agent (clopidogrel, prasugrel or ticagrelor), particularly in cases with less than one year between interventions. This is a common and quite complex situation because evidence on the safety of maintaining DAPT in the noncardiac perioperative period is scarce and indirect. The evidence is mainly extrapolated from the cardiac surgery data, which reveals a great increase in the rate of bleeding.^329,330^ On the other hand, the potential for treatment suspension is also quite high, especially after PCI, with DAPT suspension being one of the main predictors of stent thrombosis.^331^ An interesting study was conducted in France evaluating 1,134 patients with PCI who required subsequent noncardiac surgery. The study identified DAPT suspension for more than 5 days before the surgery as one of the independent predictors of perioperative cardiovascular complications.^332^

The best way to deal with DAPT in the perioperative period is to maintain the optimal duration of this therapy and not to perform elective surgeries during this period (see the topic of prophylactic myocardial revascularization in this guideline - item 7.B): 6 weeks after PCI with BMS; 6 months after DES or one year after PCI in the context of acute coronary syndromes. Some elective procedures, such as oncological treatment, cannot be postponed without consequences. In this situation, it is recommended to maintain ASA alone and suspend clopidogrel. Clopidogrel should be suspended 5 days before the surgery and restarted as soon as possible, ideally before completing 10 days without DAPT. The postoperative deadline for restarting the drug depends on adequate hemostasis control and should be individually established between the surgical and clinical teams. Basing on the evidence of relative safety of withdrawal of the second antiplatelet in up to 10 days, we recommend not to exceed a total of 10 days without DAPT outside the perioperative context.^333^

Evidence is even scarcer for the newer antiplatelet drugs. The TRITON-TIMI 38 study included patients receiving prasugrel or clopidogrel associated with ASA and requiring cardiac surgery. Prasugrel showed higher rates of bleeding than clopidogrel, even with suspension of clopidogrel or prasugrel for up to 7 days.^334^ This observation supports the recommendation to discontinue prasugrel 7 days before noncardiac surgeries.

Although pharmacokinetic data support the suspension of ticagrelor for a shorter period^,^^335^ the current recommendation is still 5 days. Sub-analysis of patients who required myocardial revascularization in the PLATO study (patients randomized to ASA + ticagrelor vs. ASA + clopidogrel) demonstrated less bleeding with ticagrelor than with clopidogrel. This finding is in agreement with the idea of a faster platelet activity recovery after suspension of ticagrelor in comparison with clopidogrel.^336,337^

The new drug eluting stents are less thrombogenic; thus, the ideal interval for noncardiac surgery is shortened to 6 months in cases of elective PCI (see specific session of prophylactic myocardial revascularization in this guideline - item 7.B). Notably, maintenance of DAPT can be considered for some procedures performed in compressible sites or by endovascular technique depending on multidisciplinary consensus.

Patients at very high risk of stent thrombosis, such as diabetic or with PCI involving grafts, or in the context of acute coronary syndromes or complicated PCI, may be considered for “bridge” therapy with parenteral antiplatelet consisting of glycoprotein IIb/IIIa inhibitors.^338^ There is no recommendation for “bridge” therapy with LMWH because recent clinical evidence has demonstrated the harm of such measure, in addition to the pharmacological need of inhibition of platelet activity rather than coagulation.^339^

### B) Myocardial Revascularization

The first studies that analyzed the role of myocardial revascularization before noncardiac surgery suggested that it could be indicated to reduce perioperative cardiovascular risk.^340,341^ This strategy aimed at reducing the risk of ischemic events related to severe and fixed coronary stenosis(342). Nevertheless, the events related to the instability of atherosclerotic plaques are not reduced. Atherosclerotic plaque rupture is a pathophysiological mechanism known to be involved in the genesis of ischemic events in the perioperative context.^68^

Recent evidence in the literature has failed to demonstrate the beneficial role of prophylactic myocardial revascularization (CABG or PCI) in patients with stable CAD in the preoperative period in noncardiac surgeries.^343,344^ In addition, the development of drug therapy and consequent perioperative pharmacoprotection have made the potential benefits of prophylactic myocardial revascularization increasingly restricted. Therefore, indications for preoperative myocardial revascularization in noncardiac surgeries are identical to those outside the perioperative context.^345^ The indications aimed not only to reduce perioperative ischemic events but also to improve long-term prognosis.

In cases with unequivocal indication of myocardial revascularization in patients who are in the preoperative period of noncardiac surgeries, information, such as clinical stability of the patient, prognosis of the underlying disease that led to the indication of the surgical procedure, and the potential risk of bleeding of this procedure, should be considered in decision-making. In these cases, the interval between myocardial revascularization and noncardiac surgery is an important factor, particularly in cases of PCI.^346-349^

When the surgery must be performed during the endothelization period of the stent, the risk of stent thrombosis and the risk of hemorrhagic complications associated with the use of double antiplatelet therapy increase. In the perioperative period, the French registry of more than 1,000 patients submitted to noncardiac surgery after PCI with stent (DES in one third of them) reaffirmed that one of the main predictors of cardiac complications is the suspension of DAPT more than 5 days before the surgery, regardless of the type of stent.^332^ Therefore, elective operations must be performed whenever possible after the end of this high-risk period.

In contrast to what we observed in the context of isolated coronary disease, DES present an enormous fear in the perioperative period and a potentially higher risk than BMS because of the greater and more lasting thrombogenicity associated to them. Thus, when noncardiac surgery is required in a near future (formerly a year, with paclitaxel stents or first-generation sirolimus stents), the use of DES is contraindicated.^331^ Consequently, when the surgical procedure needs to be performed shortly, PCI with BMS or even balloon PCI without stent should be considered, provided they present favorable primary outcome.

With most modern DES, recent evidence suggests that the duration of DAPT can be shortened to 6 months^350-352^ and exceptionally 3 months.^353,354^ On the other hand, when PCI is performed to treat acute coronary syndromes, especially in cases of MI, duration of DAPT should be one year, regardless of the type of stent implanted.^354^

Contextualizing these most recent data on shortening the duration of DAPT in the perioperative period, Holcomb et al. demonstrated that the risk of complications following noncardiac surgeries is significantly reduced from the 6th month of PCI with DES.^355^ The authors analyzed more than 20,000 cases of noncardiac surgeries after coronary PCI, with approximately half of them with DES. Notably, they also introduced another important concept of interventional treatment of acute coronary disease in the perioperative period. When PCI was performed in the context of MI even after one year, the risk of thrombotic complications is still greater than in cases where PCI was performed electively.^356^

### C) Prophylaxis for Venous Thromboembolism

The adequate prophylaxis for venous thromboembolism in the perioperative evaluation involves detailed knowledge of the risk factors of each patient together with the risks inherent to the surgical procedure.

It is important to consider that most hospitalized patients have one or more risk factors for venous thromboembolism^357-366^ and that these factors have a cumulative character (Chart 5).^360^

**Chart 5:** Risk factors for venous thromboembolism

Risk factors	
Surgery	Trauma (major traumas or lower limbs)
Immobility, paresis of lower limbs	Neoplasia
Cancer therapy (hormonal, chemotherapy, angiogenesis inhibitor, or radiotherapy)	Previous venous thromboembolism
Venous compression (tumor, hematoma, arterial abnormality)	Advanced age
Pregnancy and postpartum	Estrogen contraceptives or hormone replacement therapy
Selective estrogen receptor modulators	Erythropoiesis-stimulating agents
Acute clinical disease	Heart or respiratory failure
Inflammatory bowel disease	Nephrotic syndrome
Myeloproliferative diseases	Paroxysmal nocturnal hemoglobinuria
Obesity	Smoking
Central venous catheterization	Acquired or hereditary thrombophilia

The incidence of confirmed thromboembolism in hospitalized patients without adequate thromboprophylaxis may vary widely, depending on the type of surgery performed, as outlined in table 2.^367^

**Table 2:** Venous thromboembolism risk stratification with the type of surgery

Surgical population	Estimated risk in the absence of thromboprophylaxis* (%)
Most outpatient surgeries	< 0.5
Spinal surgery for non-malignant diseases	1.5
Gynecologic surgery for non-neoplastic disease Most thoracic surgeries Spinal surgery for malignant disease	3,0
Bariatric surgery Gynecological surgery due to neoplasia Pneumectomy Craniotomy Traumatic brain injury Spinal cord injury Other major trauma Knee or hip prosthesis surgeries	6,0

* Mechanical or pharmacological

There is strong evidence in the literature that adequate thromboprophylaxis in surgical patients is cost-effective, with an optimal cost-benefit ratio.^368^ Despite the evidence available, with more than 20 guidelines recommending its use since 1986, it is not frequently applied, compromising patient safety.^369,370^

Mechanical thromboprophylaxis should be the primary method to prevent VTE in patients at high risk of bleeding. When pharmacological prophylaxis is indicated, the doses recommended by each manufacturer should be followed. In general, we consider the following doses: UFH, 5,000 IU, subcutaneously (SC), 12/12h or 8/8h; LMWH (dalteparin 5,000 IU, SC, 1x/day; tinzaparin 4,500 IU, SC, 1x/day; enoxaparin 40 mg, SC, 1x/day); and fondaparinux, 2.5 mg, SC, 1x/day (in individuals > 50 kg). Aspirin should not be used alone in any group of patients as thromboprophylaxis for VTE.

Evaluation of renal function is fundamentally important when considering the use and dose of LMWH, fondaparinux, or other thrombotic agents excreted by the kidneys, especially in elderly, diabetic, or at high risk of bleeding individuals. In such circumstances, the use of antithrombotic drugs with renal metabolism should be avoided. Smaller doses of the drug should be used, or serum levels of the drug and its anticoagulant effect should be monitored.

#### I. Recommendations for Prophylaxis in Non-Orthopedic Surgeries

We now use more objective scores to assess the risk of thromboembolism associated with each type of surgery to better guide prophylaxis. One of these scores that can stratify the risk for venous thromboembolism with greater accuracy is the Caprini risk assessment model.^371,372^ In this model, a score is assigned to each clinical or laboratory variable (Chart 6). Based on the number of these variables and the score obtained, the categories of risk are defined (very low, low, moderate, and high) according to the risk of VTE (Chart 7).^367^

**Chart 6:** Caprini risk assessment model:^371,372^ risk stratification of general, abdominal, pelvic, urological, gynecological, vascular, and plastic and reconstructive surgeries

1 point	2 points	3 points	5 points
Age 41-60 years Small surgery BMI > 25 kg/m^2^ Edema of MMII Varicose veins Pregnancy or postpartum Hx of recurrent and unexplained spontaneous abortion Contraceptive or HRT Sepsis < 1 month Severe lung disease, including pneumonia < 1 month Abnormal lung function MI HF (< 1 month) Hx of inflammatory bowel disease Patient restricted to the bed	Age 61-74 years Arthroscopic surgery Open surgery > 45 m Laparoscopic surgery > 45 m Neoplasia Patient restricted to the bed > 72 hours Central catheter Immobilization with plaster	Age > 75 years Previous Hx of VTE Familiar Hx of VTE Factor V of Leiden Prothrombin polymorphism 20210A Lupus anticoagulant Anticardiolipin antibody High homocystein eHeparin-induced thrombocytopenia Other congenital or acquired thrombophilia	EVA < 1 month Elective hip or knee arthroplasty Fracture of hip, pelvis, or lower limbs Acute spinal cord injury (< 1 month)

BMI: body mass index; Hx: history; HRT: hormone replacement therapy; MI: acute myocardial infarction; HF: heart failure; m: minutes; VTE: venous thromboembolism; EVA: encephalic vascular accident.

**Chart 7:** Venous thromboembolism risk stratification in the absence of mechanical or pharmacological prophylaxis according to Caprini risk score

Risk category	Caprini score	VTE risk (%)
Very low	0	< 0.5
Low	1-2	1.5
Moderate	3-4	3.0
High	≥ 5	6.0

In addition to the risk of venous thromboembolism, according to risk factors attributed to the condition of the patient or to the surgical procedure, it is important to analyze risk factors for bleeding that may modify the choice of the best thromboprophylaxis. Risk factors for severe bleeding complications are described in chart 8.^367^

**Table:** A. General Risk Factors Chart 8 - Risk factors for severe hemorrhagic complications A. General risk factors

A. General risk factors	Active bleeding	
	Previous major bleeding	
	Known untreated hemorrhagic disease	
	Severe renal or hepatic insufficiency	
	Thrombocytopenia	
	Acute encephalic vascular accident	
	Uncontrolled systemic arterial hypertension	
	Lumbar puncture, epidural or spinal anesthesia in the last 4 hours or within the next 12 hours	
	Concomitant use of anticoagulant, antiplatelet agent, or thrombolytic drugs	
**B. Specific risk factors of the procedures**	**B1. Abdominal surgery**	Male, preoperative Hb < 13 g/dL, neoplasia, complex surgery (defined by two or more procedures, difficulty dissecting or more than one anastomosis)
	**B2. Pancreatoduodenectomy**	Sepsis, pancreatic fistula, sentinel bleeding
	**B3. Hepatic resection**	Number of segments, concomitant extrahepatic organ resection, primary liver neoplasia, low preoperative hemoglobin level, and thrombocytopenia
	**B4. Thoracic surgery**	Pneumectomy or extensive resection
	**B5. Procedures in which hemorrhagic complications can have serious consequences**	Craniectomy Spinal cord surgery Spinal trauma Reconstructive procedures involving free grafting

Next, we present the recommendations for specific non-orthopedic surgeries. The recommendations are no longer guided by the Caprini risk score, but according to the risk characteristics of each surgery.

#### II. Recommendations for Prophylaxis for Orthopedic Surgeries

The risk of VTE associated with major orthopedic surgeries (hip and knee prosthesis surgeries and hip fracture surgery) is one of the highest of all surgical specialties. The combined risk of VTE in a postoperative period of 35 days in untreated patients is currently estimated at 4.3%.^373^ Table 3 describes the components of this risk.^373^ Next, we present the recommendations for prophylaxis for VTE in major orthopedic surgeries.

**Table 3:** Estimated frequency of symptomatic, nonfatal venous thromboembolism after major orthopedic surgeries

	Initial prophylaxis (0-14 days PO)	Prolonged prophylaxis (15-35 days PO)	Accumulated (0-35 days PO)
No prophylaxis	VTE 2.8% (PTE 1.0%, DVT 1.8%)	VTE 1.5% (PTE 0.5%, DVT 1.0%)	VTE 4.3% (PTE 1.5%, DVT 2.8%)
LMWH	VTE 1.15% (PTE 0.35%, DVT 0.80%)	VTE 0.65% (PTE 0.20%, DVT 0.45%)	VTE 1.80% (PTE 0.55%, DVT 1.25%)

LMWH: low-molecular-weight heparin; PO: postoperative; PTE: pulmonary thromboembolism; DVT: deep venous thrombosis, VTE: venous thromboembolism.

For patients submitted to major orthopedic surgeries, regardless of the possibility of using IPC or duration of treatment, LMWH is preferred compared with other antiplatelet agents suggested as alternatives. When using LMWH, it is suggested to start administration at least 12 hours before surgery or at least 12 hours after the surgical procedure.^373^

Whenever possible, mechanical prophylaxis should be associated with IPC during hospital stay (Class of Recommendation IIa, Level of evidence C). In addition, use of apixaban or dabigatran is preferred for patients rejecting IPC or multiple subcutaneous injections.^373^

The doses of the new anticoagulants used in the studies for prophylaxis of VTE in major orthopedic surgeries are outlined in chart 9.

**Chart 9:** Doses of new anticoagulants in hip and knee prosthesis surgeries (adjust doses in patients with decreased renal function)

Rivaroxaban	**Hip prosthesis:** 10 mg/d starting 6-10h PO for 35 days **Knee prosthesis:** 10 mg/d starting 6-10h PO for 12 days
**Dabigatran**	**Hip prosthesis:** 220 mg/d starting with 110 mg 1-4h PO for 35 days **Knee prosthesis:** 220 mg/d starting with 110 mg 1-4h PO for 10 days
**Apixaban**	**Hip prosthesis:** 2.5 mg 2x/d starting 12-24h PO for 35 days **Knee prosthesis:** 2.5 mg 2x/d starting 12-24h PO for 12 days

PO: postoperative period.

### D) Anticoagulation Management in the Perioperative Period

The major challenges of anticoagulation management in the perioperative period are the interruption of anticoagulation, which temporarily increases the risk of thromboembolism and its maintenance during invasive procedures, which may increase the risk of hemorrhagic complications. Both challenges increase the risk of death.^374-379^

When assessing perioperative thromboembolic risk, recognizing the different risk situations for thromboembolism is necessary. One of them is the patient receiving anticoagulation for the prevention of venous thromboembolism (VTE). Another risk situation is the patient receiving anticoagulation in the presence of mechanical cardiac prostheses and/or AF for the prevention of arterial thromboembolism. Table 4 presents a proposal for the risk stratification of these patients. High-risk patients are those with >10% annual risk of thromboembolism; moderate risk, 5-10% annual risk of thromboembolism; and low risk, < 5% annual risk of thromboembolism.^380^

**Table 4:** Risk stratification for thromboembolism380

Risk category	Indication for antiplatelet therapy		
	**Cardiac mechanical prosthesis**	**Atrial fibrillation**	**VTE**
High*	Any mechanical mitral prosthesis Old mechanical aortic prostheses Recent stroke or TIA (< 6 months)	CHADS2 score of 5 or 6 Recent stroke or TIA (< 3 months) Rheumatic valve disease	Recent VTE (< 3 months) Severe thrombophilia†
**Moderate**	Mechanical aortic prostheses and at least one risk factor: AF, TIA, or previous stroke, SAH, DM, CHF, age > 75 years	CHADS2 score of 3 or 4	VTE 3-12 months ago Mild thrombophilia‡ New VTE Active neoplasia
**Low**	Mechanical aortic prosthesis without AF or other risk factors for stroke	CHADS2 score of 0 to 2 (no previous stroke or TIA)	VTE > 12 months without other risk factors

CHADS2 score = ICC: 1 point, SA = 1 point, age > 75 years = 1 point, DM = 1 point, stroke/TIA = 2 points.

* High-risk patients may also include those with stroke or TIA > 3 months prior to the planned surgery and CHADS2 < 5, those who had thromboembolism during the temporary cessation of antiplatelet agents, or those undergoing certain types of surgery associated with a high risk of stroke or other type of thromboembolism (heart valve replacement surgery, carotid endarterectomy, major vascular surgeries).

† Severe thrombophilia: deficiency of protein C, S, antithrombin or presence of antiphospholipid antibodies.

‡ Mild thrombophilia: heterozygous mutation of Leiden's Factor V or prothrombin gene. VTE: venous thromboembolism; SAH: systemic arterial hypertension; DM: diabetes mellitus; TIA: transient ischemic attack; CHF: congestive heart failure.

In addition to assessment of thromboembolic risk, we should consider the risk of bleeding that certain surgical procedures present during the use of antithrombotic medications. The risk of bleeding associated with each type of surgical procedure is shown in chart 10.^375^ In general, we divided the procedures in those with high risk of severe bleeding in 2 to 4 days (2 to 4%) and those with low risk (0 to 2%). Severe bleeding is generally defined as a bleeding that results in death or is intracranial or requires reoperation to be stagnant or causes a decrease in hemoglobin ≥ 2 g/dL or requires transfusion of ≥ 2 units of red blood cells.^381^

**Chart 10:** Bleeding risk according to the surgical procedure

High risk (greater risk of bleeding in 2 days between 2 and 4%)	Abdominal aortic aneurysm surgery
	Any major surgery (duration> 45 minutes)
	Bilateral knee prosthesis surgery
	Endoscopically guided fine needle aspiration procedures
	Renal biopsy
	Laminectomy
	Urologic, head and neck, abdominal, neurosurgery, breast cancer
	Polypectomy, esophageal varices, biliary sphincterotomy, pneumatic dilatation
	Transurethral resection of the prostate
**Low risk (greater risk of bleeding in 2 days between 0 and 2%)**	Abdominal hernioplasty
	Abdominal hysterectomy
	Dissection of axillary nodule
	Bronchoscopy with or without biopsy
	Carpal tunnel surgery
	Ophthalmic surgery
	Removal of central venous catheter
	Cholecystectomy
	Skin, bladder, prostate, breast, thyroid, and lymph node biopsies
	Dilation and curettage
	Gastrointestinal endoscopy, with or without biopsy, enteroscopy, biliary or pancreatic stent without sphincterotomy
	Hemorrhoid surgery
	Hydrocele surgery
	Prosthesis surgery of knee or hip, hand, shoulder, foot, and arthroscopy
	Non-coronary angiography
	Extractions and other dental surgeries

In addition to assessment of the risk of bleeding based on the type of surgical procedure, there are clinical conditions inherent to each patient that may confer a greater risk of bleeding. There are scores that can quantify the risk of bleeding based on the clinical features of patients undergoing antiplatelet therapy, such as the HAS-BLED score, which is summarized in chart 11.^382^ A HAS-BLED score ≥ 3 is associated to higher risk of bleeding (HR 11.8, 95% CI 5.6-24.9).

**Chart 11:** Components of the HAS-BLED bleeding score

Letter	Clinical features*	Points
H	Hypertension (uncontrolled blood pressure)	1
A	Abnormal kidney and liver function (1 point each)	1 or 2
S	Stroke	1
B	Tendency or predisposition to Bleeding	1
L	Labile INR (for patients taking warfarin)	1
E	Age > 65 years (Elderly)	1
D	Drugs (concomitant use of aspirin or NSAIDs) or alcoholism (1 point each)	1 or 2

* Hypertension is defined as systolic BP > 160 mmHg. Abnormal kidney function is defined by the presence of chronic dialysis or renal transplantation or serum creatinine > 2.26 mg/dL. Abnormal liver function is defined as chronic liver disease (cirrhosis) or biochemical evidence of significant liver dysfunction (bilirubin 2 times above the upper normal value, associated with liver enzymes three times higher than the normal upper value). Tendency or predisposition to bleeding is defined as history of previous bleeding or predisposition to bleeding (anemia, hemorrhagic diathesis). Labile INR refers to high INR, unstable, or within the therapeutic level for a short time (< 60% of the time). Drugs/alcoholism refers to the concomitant use of drugs, such as antiplatelet agents and non-hormonal anti-inflammatory drugs. NSAIDs: non-hormonal anti-inflammatory drugs. Modified table from Lip et al.^383^

#### I. Warfarin^380,384^

Warfarin is a vitamin K antagonist; its anticoagulant effect takes days to disappear (half-life from 36 to 42 hours) and may require similar time to reach adequate levels after surgery. Thus, patients at high risk for thromboembolism may require “bridge therapy” with parenteral antiplatelet agents, such as UFH and subcutaneous LMWHs. These two agents present faster onset and a shorter half-life, which would allow the possibility of suspending warfarin as close to the surgical procedure as possible, minimizing thromboembolic risk as much as possible.

Since the metabolism of warfarin may be influenced by several factors, such as patient age, renal function, and drug interactions, the INR should be measured on the day before surgery to ensure that it is <1.5. If the INR is >1.5, reverse it with oral vitamin K administration (1 to 2 mg) and re-evaluate it the following day.

The decision to suspend or not to suspend warfarin before the surgical procedure will depend on the combined analysis of thromboembolic risk (Table 4), risk of bleeding (Chart 11), and the patient’s own risk.

#### I. B. Patients with Moderate Risk of Thromboembolism

There is little evidence on the best course of action in patients with moderate risk of thromboembolism regarding whether to use or not to use bridge therapy. Thus, the choice should be based on the individual characteristics of each patient and the proposed surgery. Whether the patient requires bridge therapy is decided by the attending physician.

#### I. D. Urgent or Emergency Procedures^385^

The therapeutic measures used for the reversal of oral anticoagulation with warfarin will depend on how quickly normalization of prothrombin time, measured by the INR, is reached. For surgeries that can wait 18-24 hours, suspension of warfarin associated with intravenous vitamin K1 at a dose of 2.5-5 mg usually normalizes the INR when it is within the therapeutic range.^380^

If rapid normalization of INR is needed, it is necessary to replace the deficient coagulation factors with fresh frozen plasma (FFP) and prothrombin complex concentrate. The Resolution - RDC No. 10 of January 23, 2004 from the Brazillian Health Regulatory Agency (ANVISA) states that “for the correction of hemorrhage due to coumarin antiplatelet agents or rapid reversal of the effects of coumarins”, the product of choice is the prothrombin complex. As this type of concentrate is not yet broadly available in Brazilian hospitals, the use of FFP is an acceptable alternative.^386^

For the FFP, the recommended dose is 15 mL/kg of body weight and volume overload should be avoided.^387^ No standard procedure has been established for the prothrombin concentrate. Table 5 shows the doses used in health services in the United Kingdom. However, regardless of what is used to replace vitamin K-dependent factors, using vitamin K1 (2.5-5.0 mg, oral or slow venous administration) is necessary to maintain normal prothrombin values during the preoperative period.^380^

**Table 5:** Dose of prothrombin complex concentrate to be administered for reversal of anticoagulation according to the INR

INR	Dose based on factor IX
2.0-3.9	25 U/kg
4.0-5.9	35 U/kg
≥ 6.0	50 U/kg

#### II. Dabigatran^375,384,388-391^

Dabigatran is an anticoagulant drug that acts as a direct inhibitor of thrombin, reversibly blocking the conversion of fibrinogen to fibrin (factor IIa). It is a drug that acts rapidly. Its concentration peaks after 30-120 minutes. Dabigatran has a half-life of 12-17 hours and is predominantly renally excreted (80%).

This drug is approved for preventing stroke in patients with non-valvular AF, in the treatment of VTE (DVT/PE), and for the prevention of recurrent VTE and VTE in major orthopedic surgeries. However, its use is not authorized for the prevention of arterial thromboembolism in patients with mechanical valve prostheses. Because of its rapid action and shorter half-life, there is no need for bridge therapy associated with this drug.

One of the concerns associated with the use of dabigatran is the lack of specific antidotes until recently. The available possibilities were limited to the use of the prothrombin complex and hemodialysis, which had limited success. The first antidote for thrombin inhibitors (dabigatran), the idarucizumab, was FDA approved in the USA in October 2015. Idarucizumab completely reversed the anticoagulant effect of dabigatran in phase I and phase III studies. Another promising agent under study is Arapazine (PER-977), which has been shown to reverse the anticoagulant action of dabigatran, as well as rivaroxaban, apixaban, and LMWH.^392^

#### III. Rivaroxaban^375,384,388,389,391^

Rivaroxaban is a drug that acts as a factor Xa inhibitor, blocking its enzymatic function of converting prothrombin to thrombin. It is also a fast-acting substance. Its concentration peaks after 2-4 hours and has a short half-life (5-9 hours in young people and 11-13 hours in the elderly). This drug undergoes liver metabolism and renal excretion (66%).

Rivaroxaban is approved for the prevention of stroke in patients with non-valvular AF, in the treatment of VTE (DVT/PE), in the prevention of recurrent VTE, and in the prevention of VTE in major orthopedic surgeries. However, its use is not authorized for the prevention of arterial thromboembolism in patients with mechanical valve prostheses. Since it is fast-acting and has a shorter half-life, there is no need for bridge therapy associated with this drug.

In the past, only the prothrombin complex was available to reverse the effect of rivaroxaban since there were no specific antidotes available. Currently, Andexanet alfa (PRT064445) is a specific antidote against factor Xa inhibitors. It shows a rapid reversal of the anticoagulant effect of apixaban and rivaroxaban in minutes, as observed in two recent parallel phase III studies, ANEXA-A and ANEEXA-R, respectively. Currently, ANNEXA-4, a phase IV study, is being performed. Another promising agent under study is Arapazine (PER-977), which shows an effect in reversing the anticoagulant action of dabigatran, as well as rivaroxaban, apixaban, and LMWH.^392^

#### IV. Apixaban^375,388,389,391^

Apixaban is also a factor Xa inhibitor that blocks the conversion of prothrombin to thrombin. It has a rapid onset of action. Its concentration peaks after 3 hours and has a short half-life (8-15 hours). This drug undergoes liver metabolism and renal (27%) and fecal excretion. Apixaban is approved for the prevention of stroke in patients with non-valvular AF, prevention of VTE in major orthopedic surgeries, and treatment of DVT and PE. Its use is not authorized for the prevention of arterial thromboembolism in patients with mechanical valve prostheses. Due to its rapid onset of action and shorter half-life, there is no need for bridge therapy associated with this drug.

Currently, Andexanet alfa (PRT064445) is the specific antidote against factor Xa inhibitors. It shows a rapid reversal of the anticoagulant effect of apixaban and rivaroxaban in minutes, as observed in two recent parallel phase III studies, ANNEXA-A and ANEEXA-R, respectively. A phase IV study, ANNEXA-4, is underway. Another promising agent under study is Arapazine (PER-977), which shows an effect in reversing the anticoagulant action of dabigatran, as well as rivaroxaban, apixaban, and LMWH.^392^

#### V. Edoxaban^393^

Edoxaban is also a factor Xa inhibitor. It has a rapid onset of action. Its concentration peaks in 1-2 hours. This drug has a short half-life (10-14 hours) and undergoes renal (50%) and biliary and intestinal (50%) excretion. Edoxaban is indicated for the prevention of arterial thromboembolic phenomena in patients with non-valvular AF and in the treatment of DVT or PE, but it has not yet been released in Brazil.

At present, there are no studies investigating specific antidotes for edoxaban. An option would be to use the prothrombin complex for the occurrence of bleeding that necessitates the reversal of its effect. Because it is the newest oral anticoagulant, studies evaluating its use in the perioperative period are very limited.

In principle, the most accepted approach is interruption of edoxaban 24 hours before surgeries with low risk of bleeding and interruption 48-72 hours before surgeries associated with high risk of bleeding (Chart 10).

### E) Prophylaxis of Infective Endocarditis

Despite advances in health care, infective endocarditis remains a disease of high morbidity and mortality.^394,395^ In the last decades, we have witnessed major debates on which strategies are truly effective in reducing its prevalence.

The main cause for the occurrence of endocarditis is endothelial lesion due to cardiac anatomic predisposition. Consequently, there is deposition of platelets and fibrin in the endocardium, generating non-bacterial thrombotic endocarditis. The presence of circulating microorganisms in the bloodstream may result in infective endocarditis. Other predisposing factors are the presence of vascular devices and/or infectious agent of high virulence. These can cause the disease even in individuals with a structurally normal heart.^395^ Bacteria are the most common etiological agents. Thus, several studies have evaluated the risk of spontaneous bacteremia related to routine activities and invasive procedures.

#### I. Dental Procedures

Early studies correlated dental extraction with the presence of transient bacteremia.^396,397^ Others indicated that endodontic and periodontal manipulation may lead to similar levels of bacteremia.^398-400^ Based on this, experimental animal models confirmed the reduction of bacteremia after dental manipulation with the use of prophylactic antibiotic therapy.^401,402^ Since then, this recommendation has been established for individuals with an anatomical predisposition to endocarditis.

More recently, the impact of prophylaxis on the prevention of endocarditis has been questioned. Clinical trials showed a low prevalence of infectious endocarditis (IE) presumably related to dental treatments, ranging from 2.7 to 13%.^403-405^ Moreover, it has been shown that daily activities, such as mastication, tooth brushing, and flossing are related to transient bacteremia.^400,406-409^ Other arguments against the use of prophylaxis are risk of anaphylaxis associated with the use of penicillin, efficacy proven only in experimental studies, and possibility of induction of bacterial resistance.^408,410^

Based on these arguments, the recommendation not to use prophylaxis for endocarditis has been instituted in the United Kingdom by the National Institute for Health and Clinical Excellence (NICE) since 2008.^411^ In France, prophylaxis has been recommended for high-risk individuals only since 2002.^412^ The same recommendation has been made by the American Heart Association (AHA) since 2007^413^ and the European Society of Cardiology (ESC) since 2009.^414^ The population considered to be at high risk is composed of individuals with a greater chance of developing complications and die due to illness (severe IE risk conditions). The individuals described in chart 12 are considered at risk of IE.^413,414^

**Chart 12:** Patients with infectious endocarditis risk

**Severe IE Risk Conditions**	Valve heart prosthesis
	Valvular heart disease corrected with prosthetic material
	History of infective endocarditis
	Uncorrected cyanogenic congenital heart disease
	Congenital cardiomyopathy corrected with prosthetic material (first 6 months)
	Corrected cyanogenic congenital cardiomyopathy with residual lesion
	Valvular heart disease in a transplanted cardiac patient
**Other risk conditions for IE**	Valvular heart disease (mild, moderate, or severe)*

* In case of prolapse of mitral valve, only if moderate or severe valve insufficiency is present. IE: infectious endocarditis.

Recent observational studies have shown no increase in the number of endocarditis following recommendations for prophylaxis in high-risk individuals in France and the United States.^415-417^ However, an observational study in the United Kingdom showed an increase in the incidence of infective endocarditis since the NICE recommendations in 2008.^418^ In this country, a study performed in 2012 revealed that most cardiologists and cardiac surgeons believed that prophylaxis should be performed in cases of valve prosthesis and previous endocarditis.^419^ In the USA, following the new AHA recommendations in 2007, one study showed an increase in the incidence of hospitalizations for streptococcal endocarditis.^420^ On the other hand, the limitations imposed on observational cohorts should be considered.

Considering that most patients with valvular heart disease in Brazil present characteristics different from those currently observed in the USA and European countries (young people with rheumatic sequelae and higher lethality due to endocarditis) and the lack of prophylaxis studies in Brazil, prophylaxis is recommended for patients with native valve injury, although they do not have a valve prosthesis. Another difference of our population compared to the American and European populations is the higher prevalence of individuals with low access to health care and therefore with lower dental hygiene and higher risk of bacteremia after invasive dental procedures.^400,421^

Although cited in the international literature, a significant adverse effect of antimicrobial therapy is a rare event. Therefore, use of prophylaxis for endocarditis is recommended prior to dental procedures involving the manipulation of gingival tissue, periodontal region, or oral mucosa perforation (Chart 13) for all individuals with anatomically relevant valve disease (Chart 12). The antibiotic should be given as a single dose 30-60 minutes before the procedure (Table 6).

**Chart 13:** Dental procedures and indication for prophylaxis of infective endocarditis

Prophylaxis indicated	For patients scheduled for procedures involving manipulation of gingival tissue, periodontal region, or perforation of the oral mucosa
**Prophylaxis not indicated**	Local anesthesia in uninfected tissue
	Dental radiography
	Placement, adjustments, or removal of orthodontic appliances
	Natural fall of baby tooth
	Bleeding from trauma to the oral mucosa or lips

**Table 6:** Prophylaxis schemes before dental procedures

Route of administration		Antibiotic	Adult dose	Child dose
Oral		Amoxicillin	2 g	50 mg/kg
	Allergy to penicillin	Clindamycin	600 mg	20 mg/kg
		Cephalexin	2 g	50 mg/kg
		Azithromycin	500 mg	15 mg/kg
		Clarithromycin	500 mg	15 mg/kg
Parenteral (IV or IM)		Ampicillin	2 g	50 mg/kg
		Cefazolin	1 g	50 mg/kg
		Ceftriaxone	1 g	50 mg/kg
	Allergy to penicillin	Clindamycin	600 mg	20 mg/kg
		Cefazolin	1 g	50 mg/kg
		Ceftriaxone	1 g	50 mg/kg

It should be noted that infective endocarditis is a more frequent result of bacteremia from daily activities than after dental procedures. There is no doubt that maintaining good oral health is the best strategy to prevent endocarditis. In individuals with periodontal and endodontic diseases, the incidence and magnitude of bacteremia in daily care and during procedures are higher compared to individuals with healthy teeth.^421^ Thus, we recommend emphasizing daily dental care and biannual dental evaluation.

#### II. Respiratory Tract Procedures

Patients submitted to an incision or biopsy of the respiratory tract mucosa, such as otorhinolaryngological surgeries, should receive an antibiotic treatment scheme similar to the one used before dental treatment with high risk of bacteremia. There is no recommendation for prophylaxis for bronchoscopy, laryngoscopy, and orotracheal intubation. For infection treatments, such as abscess drainage, antibiotic prophylaxis with antistreptococcal action should also be administered.^414^

#### III. Genitourinary and Gastrointestinal Tract Procedures

Despite limited evidence, it is believed that patients at high risk for infective endocarditis (Chart 12) would probably benefit from prophylaxis before genitourinary or gastrointestinal procedures. Patients with non-high-risk valvular heart disease may also benefit from prophylaxis before these procedures (Chart 12). The recommended antibiotic treatment scheme for this group is in table 7.

**Table 7:** Prophylaxis schemes before genitourinary and gastrointestinal procedures

Antibiotic	Adult dose	Child dose
Venous ampicillin* +	2 g	50 mg/kg
Venous gentamicin	1,5 mg/kg	1,5 mg/kg
Allergy to penicillin:		
Venous vancomycin +	1 g	20 mg/kg
Venous gentamicin	1,5 mg/kg	1,5 mg/kg

* Reinforcement with venous ampicillin 1 g 6h after the procedure.

#### IV. Dermatological and Skeletal Muscle Procedures

For treatment of infections, such as abscess drainage, antibiotic prophylaxis should be administered with antistaphylococcal and antistreptococcal action.^414^

#### V. Piercing and Tattooing

The number of reports of infective endocarditis related to piercing and tattooing has increased, mainly associated with tongue piercing, but the risk was not estimated.^422^ Therefore, patients should be warned about this risk.

### F) Surveillance for Cardiovascular Complications

Early detection of cardiovascular events is critical to reduce mortality after noncardiac surgeries. MI can occur in the absence of chest pain and is thus necessary to develop monitoring strategies for its diagnosis.

ST-segment monitoring, serial 12-lead ECG, and measurement of cardiac troponin levels are methods used to monitor complications. Studies evaluating the use of continuous ST-segment monitoring have shown that this method has a large sensitivity (between 55 and 100%) and specificity (between 37 and 85%) range for the detection of perioperative ischemia (intra- and postoperatively) because its effectiveness depends on the technique used and the baseline features of the population.^423-426^ The occurrence of postoperative ischemia detected with continuous monitoring in patients submitted to vascular surgeries has prognostic implication and is an independent predictor of long-term cardiovascular events.^427,428^ However, since measurement of perioperative troponin levels (a simpler test) became available, the use of automatic monitoring for diagnosis and prognosis of perioperative myocardial ischemia has been discontinued. It has not been studied further and is therefore not recommended.

In the absence of electrocardiographic changes or clinical condition suggestive of ischemia or echocardiographic changes compatible with MI, increases in conventional troponin levels following noncardiac surgeries is associated with a higher rate of cardiovascular events in the short and long term, as shown in several studies.^428-433^ In a meta-analysis of patients submitted to vascular surgeries in 2011, the authors demonstrated that increases in TnI levels postoperatively without MI features was a mortality predictor in a period of 30 days, with a mortality rate of 11.6%. Patients with normal troponin levels and patients with MI had mortality rates of 2.3% and 21.6%, respectively.^434^ In 2012, in the VISION study involving 15,133 patients, the authors demonstrated a significant association between the peak of fourth-generation troponin T (TnT) and mortality rate in a period of 30 days.^305^ Although there is no sufficient evidence regarding the best strategy to manage cases of increase in troponin levels, we recommend performing a non-invasive or invasive complementary investigation with cardiac risk stratification based on the specific evaluation of the cardiologist before hospital discharge.

The use of hs-Tn kits significantly improved the accuracy to rapidly confirm or exclude diagnosis of MI in patients with chest pain in the emergency room.^100,435^ However, its interpretation is still a challenge in the perioperative period. Since 2011, observational studies have evaluated the behavior of hs-TnT in the postoperative period. The studies found an increase of hs-TnT levels above the 99th percentile (14 ng/L) in 45-60% of patients after noncardiac surgeries.^103,104,106,107^ In some studies, this increase is related to mortality in the long term.^103^ Recently, a study correlated the increase of hs-TnT with noncardiac complications in a period of 30 days after abdominal surgeries.^436^

Only one study with 135 patients investigated the hs-TnI in the perioperative period. The authors observed a correlation between increases in hs-TnI levels and mortality.^437^ To date, the relevance of isolated increases in hs-Tn levels in the postoperative period remains uncertain. Several conditions may be related to a baseline (chronic) increase in hs-Tn, such as advanced age, HF, CAD, valve diseases, chronic renal failure, or other chronic noncardiac diseases. Therefore, hs-Tn should be dosed preoperatively to determine its baseline value (see item 3.E.I). Even so, no reference value has been established for the value of the variation (delta) that correlates with cardiovascular events or mortality.^438^ Whether an increase in perioperative hs-TnT is related only to general mortality or cardiovascular events is difficult to differentiate. Thus, it is harder in clinical practice to determine whether performing additional cardiovascular risk stratification measures will improve the prognosis of the patients.

**Figure 2:**
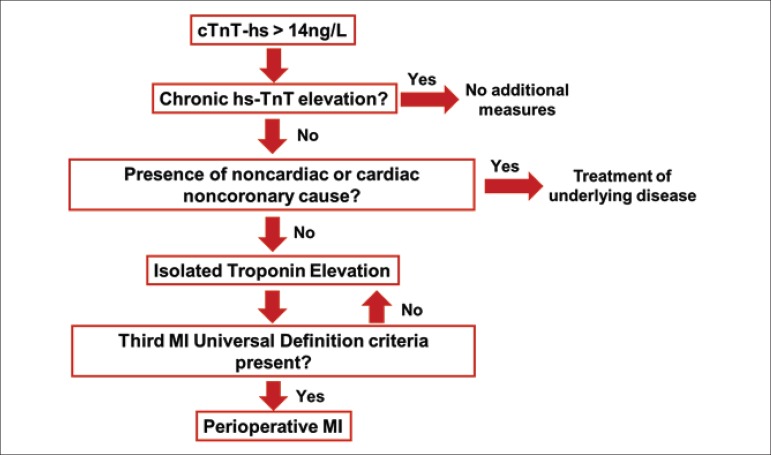
Flowchart for the evaluation of patients with hs-Tn in the postoperative period

On the other hand, whenever the patient shows increased troponin levels alone, alternative diagnoses that may lead to increases in troponin and are frequent in the perioperative period, such as pulmonary thromboembolism (PTE), acute pericarditis, decompensated HF, arrhythmias, myocarditis, sepsis, shock, or renal failure, should be avoided.^439^ We recommend the use of the flowchart shown in figure 2 for the evaluation of patients with hs-Tn levels above the 99th percentile after surgery.

Most cardiovascular events occur until the third postoperative day. The use of serial 12-lead ECG during this period is a simple and effective method for detecting events. In a study involving 3,564 patients more than 50 years old, detection of ischemia using postoperative ECG is an independent predictor of cardiovascular events. However, a negative ECG for ischemia does not reduce the risk of events.^440^ In another study comparing serial ECG with 3-lead Holter in 55 patients submitted to vascular surgeries, the ECG is as effective as the Holter for detecting myocardial ischemia.^441^ Troponin dosage associated with serial ECG until the third postoperative period is the best strategy for the diagnosis of MI.^442^ Notably, these ECG studies were performed before the availability of highly sensitive troponins.

## 8. Diagnosis and Treatment of Cardiovascular Complications in the Perioperative Period

### A) Acute Coronary Syndromes in the Perioperative

MI is the most feared cardiac complication in the perioperative period, occurring in 0.3-3% of low-risk patients with no history of CAD and reaching 33% in high-risk patients with a history of CAD.^69^ MI shows high mortality rates (40-50%),^443^ probably related to the existence of comorbidities, diagnostic difficulty, and limitations to use of antithrombotic and antiplatelet drugs. About 50% of perioperative MI is due to instability of atherosclerosis plaques, and the remainder is due to imbalances between supply and consumption of oxygen,^68^ which should be considered not only in acute treatment but also in the establishment of prevention strategies.

Although clinical consequences of perioperative MI are extremely serious, diagnosis is often not obvious and requires a high degree of clinical suspicion. Most perioperative ischemic events occur within the first three days after the surgical procedure. The classic clinical feature of precordial pain is absent in more than half of the patients,^68,69,444^ which is partially explained by the residual effect of analgesics or sedatives used in that period. In addition, when chest pain is present, it is often attributed to other more obvious etiologies, such as incisional pain or position of the patient. Other manifestations, such as dyspnea and nausea, have alternative explanations in this period (atelectasis, medication effect). Thus, perioperative MI is frequently undervalued by the medical team. Since it is difficult to interpret the clinical findings, analysis of complementary tests is fundamental for the diagnosis of perioperative myocardial ischemia. Among these, the ECG, the markers of myocardial necrosis, and the transthoracic echocardiogram should be considered.

Regarding the analysis of ECG, ischemic alterations should be distinguished from other causes of ECG alterations, such as electrolytic imbalances, hypothermia, drug effects, and incorrect positioning of the leads. Evolutionary pattern should also be considered in the analysis of ECG. It is important to compare the changes in the traces before and after the event.

Among the markers of myocardial necrosis, troponin is undoubtedly the most used due to its high sensitivity and specificity in the diagnosis of myocardial injury. However, this marker is increased in other situations of myocardial injury, in addition to the one caused by obstructive coronary disease. Other complications commonly present in the postoperative period of noncardiac surgeries are pulmonary embolism, HF, arrhythmias, and sepsis, which also increase the levels of markers of myocardial necrosis and should be considered in the differential diagnosis. In addition, patients with renal failure commonly present increases in troponin levels, particularly TnT, but show a steady evolutionary behavior without the typical increase and decrease pattern of MI. On the other hand, CKMB dosage is less useful for the diagnosis of perioperative MI because of its lower sensitivity and specificity compared to troponin. This marker may increase after skeletal muscle injury during surgery, and its relationship with CPK has low reliability in the identification of perioperative myocardial injury.^428^

Transthoracic echocardiography is an important tool for the diagnosis. Although a normal test does not exclude the diagnosis, presence of a new alteration in segmental contractility in patients with suspected myocardial ischemia corroborates the diagnosis. It can also provide indirect data for alternative diagnoses, such as pulmonary embolism and non-ischemic HF.

**Figure 3:**
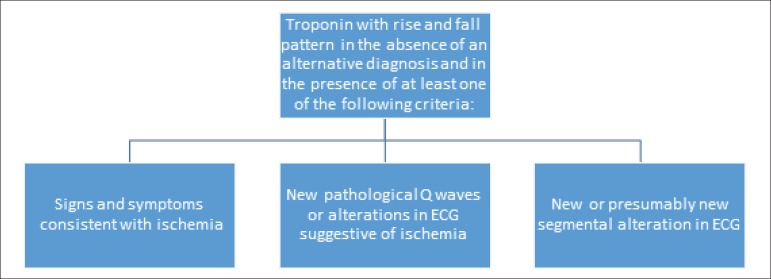
Strategy for the diagnosis of perioperative MI. ECG: Electrocardiogram.

It is important to note that analysis of isolated data cannot confirm or exclude the diagnosis of perioperative myocardial ischemia. Although recent publications define very clearly the criteria for the diagnosis of myocardial infarction, perioperative MI remains without well-defined criteria.^445^ The diagnostic strategy proposed by this guideline for the identification of patients with perioperative MI is presented in figure 3.

In 2014, the authors of this study proposed prognostic criteria for patients with isolated troponin increases postoperatively based on data from the VISION study.^446^ Patients were diagnosed if they presented increases in fourth-generation TnT above the 99th percentile (30 ng/L) without another alternative diagnosis that could explain this result. Although the authors did not use the universal definition of MI, they created the first prognostic score for these patients. They found that age above 75 years (1 point), presence of anterior wall ischemia on ECG (1 point), and alterations in ST-segment (2 points) are independent predictors of mortality in a period of 30 days (Table 8).^446^

**Table 8:** 30-day mortality risk score in patients with isolate increase in troponin levels

Score	Mortality (%)
0	6
1	9.4
2	22.1
3	29.4
4	62.5

Despite the frequency and prognostic importance, data in the literature are limited with regard to the treatment of perioperative myocardial ischemia. Most of the interventions represent extrapolations of well-established data for acute coronary syndromes not related to surgical procedures. However, all therapeutic strategies require measures that lead to an increased risk of postoperative bleeding. Thus, individualized measures and constant interaction with the surgical team are required.

The treatment of MI with no alterations in ST-segment (most cases of perioperative MI) initially requires correction of triggering factors and may perpetuate the ischemic process. Correction of anemia, hypovolemia, and pressure oscillations is the primary measure to be considered in this situation. To achieve consistency with the pathophysiology of the event, stabilization of the coronary plaque should be considered an important measure in the treatment. The extrapolated recommendations for the treatment of spontaneous acute coronary syndrome, antiplatelet treatment with ASA and clopidogrel, and anticoagulation with UFH or LMWH are used.^447^ However, one should always weigh the risk of bleeding and the benefit of anticoagulation. Despite the absence of randomized studies in the perioperative period, it is prudent to give preference to UFH in cases of increased risk of bleeding because its effect can be quickly reversed in cases of bleeding. Patients of higher ischemic risk, i.e., those more than 75 years or with anterior wall ischemia on the ECG or clinical or hemodynamic instability, should be referred for early invasive strategy and revascularization. The remaining patients should be submitted to stratification before discharge. Such practice is fundamental to control the alarming morbidity and mortality in the short and long term.^69,448^

MI with ST-segment alterations occurs in a minority of cases and presupposes total occlusion of the coronary artery, requiring immediate intervention. In contrast to MI not related to surgical interventions, thrombolytic therapy is strongly contraindicated in the perioperative period because of prohibitive risk of bleeding. Thus, coronary angiography with primary PCI is the treatment of choice for these patients. This strategy is safe and feasible in those patients considered to be without contraindications to heparin and antiplatelet therapy, which are required during and after the procedure, respectively.^69,449^

### B) Acute Atrial Fribrilation/Flutter

In the perioperative period, patients may present a variable risk of developing AF. The definition is based on the risk factors of the patient (male gender, advanced age, presence of cardiovascular comorbidities) and type of surgery (thoracic, mainly esophageal and lung surgeries). The reasons for higher occurrence of AF in thoracic surgeries are elevated levels of catecholamines, hypervolemia, right ventricular overload, pericarditis, and marked systemic inflammatory response.

The incidence of AF in the perioperative period of noncardiac surgeries (POAF) varies with the characteristics of the patients and the type of surgery. The incidence can vary from 3% in adults > 45 years up to 30% in thoracic surgeries. It usually presents between the second and fourth postoperative days.^450^ High ventricular response AF is the most common presentation and may compromise hemodynamics, which may result in hypotension, HF, and myocardial infarction.^451^ The triggering factors of atrial arrhythmia are increased sympathetic activity caused by surgical stress, pain, and anemia, in addition to hypotension and hypo or hypervolemia. Hypoxia also causes AF due to vasoconstriction of the pulmonary veins and increases in right atrial pressure and atrial myocardial ischemia.^452^

Atrial flutter (FLU) may have the same mechanism of AF but may also occur only due to autonomic imbalance, similar to other paroxysmal supraventricular tachycardias. Due to association with cardioembolic events and hemodynamic impairment as in AF, FLU can be diagnosed and managed in a similar manner.

Diagnosis is performed using 12-lead ECG or detection on a cardiac monitor for more than 30 seconds.^450^ The initial measure for AF/FLU is to identify the triggering factors and to correct them early. Most POAFs have spontaneous reversal in 24 hours. If arrhythmia persists, the initial aim is to control the heart rate, which may remain between 80 and 110 bpm or up to 120 and 130 bpm according to clinical decision (hemodynamic stability and transient situations of increased adrenergic stress). The most commonly used medications for heart rate control are metoprolol, diltiazem, and digoxin (or deslanosid C, if only the venous route is available).^453^

Digital use requires slow titration, adequate electrolytic control (calcium, potassium, and magnesium), and monitoring renal function or, in specific cases, digoxinemia. Its efficacy may be compromised by the degree of sympathetic activity in the perioperative period. Diltiazem should not be used in patients with hypotension or with ventricular dysfunction because of its negative inotropic effect. In these patients, the use of β-blockers is preferred.^452,454^ Some studies indicated the use of venous magnesium or chloride sulfate, which can reverse arrhythmia because of its effect on T- and L-type calcium channels by reducing atrial automatism and heart rate control (inhibition of AV conduction), with a lower hypotensive or inotropic negative effect.^455^

During the surgical period, patients present hypercoagulability associated with the risk of bleeding. Thus, most consensuses recommend anticoagulation for the prevention of arterial embolism only after 48 hours of persistent arrhythmia.^452,454^ Clinical scores used to determine the risk of ischemic event and bleeding were not evaluated in the perioperative period. However, the American directive of 2014 recommends the routine use of CHA2DS2-VASC scores for embolic risk and HAS-BLED for bleeding risk.^454^ Considering surgical recovery, anemia, hemodynamic stability, and surgical wound, special attention should be given to the risk of bleeding.

### C) Acute Heart Failure

The influence of chronic HF on perioperative risk is well known, with an increase in death of 63% and a readmission rate of 51% in a period of 30 days, compared to patients with CAD but without HF.^126^ However, publications on acute perioperative HF in noncardiac surgeries are limited. On the other hand, when HF has recently been diagnosed and it is possible to extrapolate that patients are at least moderately symptomatic or with signs of congestion, there is a clear recommendation for an elective surgery to be postponed until symptoms subside and the reverse remodeling process begins (improvement of ventricular dysfunction and reduction of diastolic volume), following administration and optimization of drugs, such as ACE inhibitors or angiotensin II receptor blockers (ARBs) aldosterone antagonists, and β-blockers.^456^

We can analyze the presence of acute HF through evaluation of natriuretic peptides. Levels of B-type natriuretic peptide (BNP) or amino terminal portion of ProBNP (NT-proBNP) in the circulation increase when there is ventricular dysfunction. They are particularly increased if ventricular wall tension or fiber stretching exists and are therefore significantly increased in acute HF. Mildly or moderately increased levels have already shown an important relation with morbidity and mortality. In a study of 297 patients more than 50 years old submitted to emergency procedures, Farzi et al.^457^ observed a sevenfold increase in the risk of cardiovascular events (nonfatal MI, acute HF, or cardiovascular death) during hospitalization in patients with NT-proBNP above 1,740 mg/mL and patients with NT-proBNP > 1,600 pg/mL showed a fourfold increase in the rate of combined events. The importance of this study relies on the fact that high NT-proBNP levels are compatible with the expected values in patients with acute HF (usually > 1,800 pg/mL defines the patient with acute dyspnea of cardiac cause). Another study evaluated patients with hip fracture submitted to emergency surgery and analyzed the relationship between NT-proBNP and the risk of death. High (> 2,370 pg/mL) and intermediate (806-2,370 pg/mL) NT-proBNP levels are associated with a significantly higher mortality compared to patients with low levels (< 806 pg/mL) - (15 vs. 11 vs. 2%, p = 0.04). In the long term, mortality is also higher in these two groups (69% vs. 49% vs. 27%, p < 0.001).^458^ Patients with such increased levels of natriuretic peptides in the preoperative period are probably no longer adequately compensated for HF at the time of surgery and this may be one of the causes of postoperative acute HF.

A multicenter study compared 5,094 patients with worsening HF to 5,094 patients without HF, paired by baseline characteristics, submitted to noncardiac surgery. Worsening HF in the perioperative period was associated with a twofold increase in mortality in a period of 30 days (p < 0.001), 1.5-fold increase in postoperative morbidity (p < 0.001), increased risk of developing renal failure, need for mechanical ventilation for more than 48 hours, pneumonia, cardiac arrest, unplanned intubation, sepsis, and urinary tract infection (all p < 0.05). BNP or NT-proBNP was not evaluated in this series, and the incidence of myocardial infarction was similar in both groups (p = 0.7).^459^

Therefore, patients who are not compensated for HF should not be submitted to elective surgeries because they have a very high risk of developing HF. Studies evaluating the real incidence, cause, diagnosis, and treatment of acute postoperative HF are required. Diagnosis of acute postoperative HF is clinical, and the dosage of natriuretic peptides can be performed in cases of diagnostic uncertainty. The echocardiogram should be performed to evaluate the presence of basic structural heart disease. Treatment should be performed in the same manner as that of acute HF outside the perioperative period. The possible causes of acute postoperative HF are acute CAD, persistently positive perioperative water balance volume overload, involuntary suspension of drugs used to treat chronic HF, renal failure, infection, PTE, and arrhythmias, among others.

Among these causes, MI is more common in the first 72 hours of the postoperative period.^69,444^ It may manifest as acute HF or acute pulmonary edema and not as chest pain.^446^ MI should always be actively investigated with ECG and serial troponin collection. The echocardiogram may also help in the diagnosis by showing new changes in segmental contractility.

### D) Venous Thromboembolism

#### I. Diagnosis of Venous Thromboembolism

DVT and PTE are two manifestations of the same disease, the venous thromboembolism (VTE). There are clinical probability scores that can be used for the diagnosis of VTE. One of the most used is the Wells score (Table 9 for DVT and table 10 for PTE).^460,461^

**Table 9:** Wells score for probability of deep venous thrombosis

Criteria	Points
Neoplasm	+1
Recent limb paralysis or immobilization	+1
In bed for >3 days or surgery <4 weeks	+1
Palpation pain of deep venous system	+1
Edema of the whole leg	+1
Difference >3 cm in calf diameter	+1
Asymmetrical compromised leg edema	+1
Dilation of superficial veins (affected limb)	+1
Another alternative diagnosis more likely than DVT	-2
**Probability of DVT**	**Points**
Low	0
Moderate	1 to 2
High	≥ 3

DVT: deep venous thrombosis.

**Table 10:** Wells score for probability of pulmonary thromboembolism

Criteria	Points
Previous VTE	1.5
Recent VTE	1.5
Malignancy	1.0
Hemoptysis	1.0
Heart rate >100 bpm	1.5
Signs of DVT	3.0
Most likely diagnosis	3.0
**Probability of PTE**	**Points**
Low	0 to 1
Moderate	2 to 6
High	≥7

VTE: venous thromboembolism; PTE: pulmonary thromboembolism.

#### I. A. Deep Venous Thrombosis

**Figure 4:**
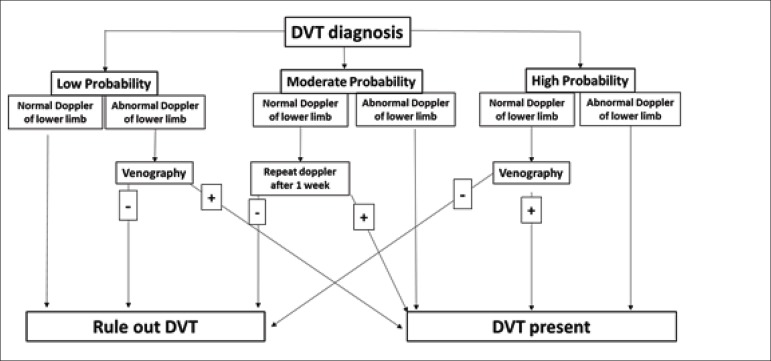
Flowchart for the diagnosis of deep venous thrombosis. DVT: deep venous thrombosis.

DVT of the lower limbs is subdivided into two categories, namely, distal venous thrombosis (calf veins) and proximal vein (popliteal, femoral, or iliac veins). Proximal venous thrombosis is the most commonly associated with PTE. The diagnosis is performed with history and clinical examination (edema, pain, and erythema involving the site) and confirmed by imaging tests. The proposed flowchart for the diagnosis of DVT is shown in figure 4.

Venous Doppler is the test of choice, with a positive predictive value of 94% and has the advantage of being conducted at the bedside.462,463 However, venous Doppler has limitations in detecting isolated thrombi in the iliac veins and in the portion of the femoral vein in the adductor canal.

D-dimer dosage should not be used alone for the diagnosis of VTE. D-Dimer is a product of fibrin degradation and is increased (> 500 ng/mL equivalent units of fibrinogen) in virtually all VTE patients. However, this test has high sensitivity and low specificity and may be increased in the elderly, patients with neoplasms, renal failure, pregnancy, and patients recently submitted to surgeries.^464^

Iodinated contrast venography can be used when the venous Doppler cannot be performed or gave an uncertain result. Venography can cause discomfort to the patient, besides the greater difficulty in obtaining an adequate study. It has an accuracy similar to the venous Doppler.^465^

Magnetic resonance venography (MRV) has the same accuracy as contrast venography (100% sensitivity and 96% specificity). Its major limitation is the high cost, but it is an option when the patient has allergy to iodinated contrast.^466^

Angio-CT of the chest with PTE protocol allows visualization of the pulmonary arteries and subdiaphragmatic deep veins, including the lower limbs, in the same test without the need for additional doses of iodinated contrast.^467^ In some studies, Angio-CT venography is comparable to venous Doppler for the diagnosis of femoral-popliteal venous thrombosis.^468^ However, to date, the use of Angio-CT remains a potential test for simplifying the diagnosis of DVT. Future studies are still required to establish its accuracy.

#### I. B. Pulmonary Thromboembolism

**Figure 5:**
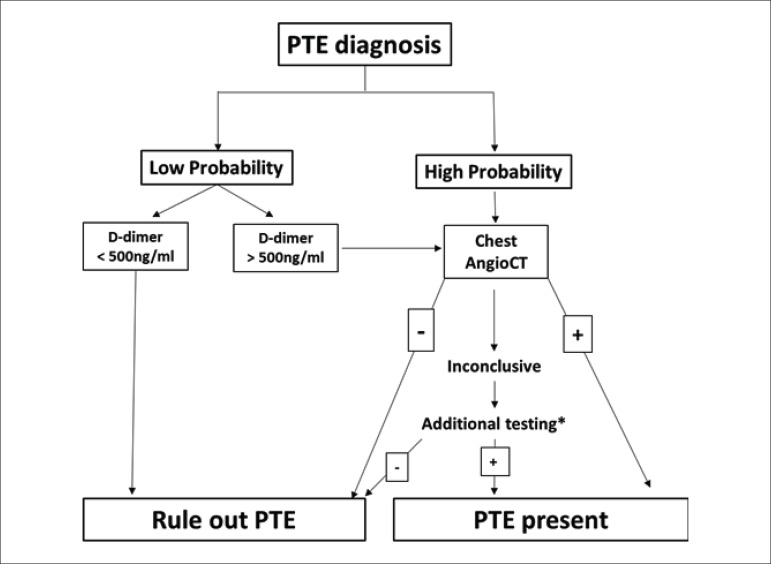
Flowchart for the diagnosis of PTE PTE: pulmonary thromboembolism; Chest AngioCT: computed tomography angiography of the chest. * Ventilation/perfusion scintigraphy; contrasted pulmonary angiotomography; serial Doppler of lower limbs; pulmonary angiography by nuclear magnetic resonance.

Acute PTE is a common and often fatal disease. Clinical evaluation and diagnostic tests are required before the start of anticoagulation (Figure 5).

The diagnosis is performed with history and physical examination, ranging from the absence of symptoms to shock or sudden death. The most common symptoms identified in the PIOPED II (Prospective Investigation of Pulmonary Embolism Diagnosis II) study^469^ are dyspnea (73%), pleuritic pain, and cough (37%). Dyspnea is often sudden. Approximately 10% of patients present with symptoms of pulmonary infarction, usually due to small and peripheral embolisms. However, in a systematic review of 28 studies with a total of 5,233 patients with DVT, one-third had asymptomatic PTE.^470^

The incidence of shock is 8%. Massive PTE can be accompanied by right ventricular failure, with increased jugular venous pressure, presence of a third sound on the right side, cyanosis, and obstructive shock. However, patients with severe PH and underlying cardiopulmonary diseases may present shock with small PTE.

#### Complementary Tests

**Arterial blood gas:** this test is usually altered. However, it is neither sensitive nor specific for the diagnosis of PTE. Hypoxemia is present in 74% of cases.

**BNP and troponin:** they may be increased but are not sensitive or specific tests for the diagnosis of PTE. They have prognostic implications, being indicative of the severity of PTE.

**D-dimer:** D-dimer, as well as for DVT, is a sensitive but not very specific test.

**ECG:** ECG alterations in patients with PTE are common, although not specific. Tachycardia and ST-segment and T-wave alterations are the most frequent findings (70% of cases of PTE). Classical alterations considered as suggestive of PTE (S1 Q3 T3, right ventricular overload, and incomplete right branch block) are not frequent (< 10%). Electrocardiographic changes that are associated with a worse prognosis are atrial arrhythmias (e.g., AF), bradycardia (< 50 bpm) or tachycardia (> 100 bpm), new right branch block, Q waves in lower leads (DII, DIII, and aVF), ST-segment changes in anterior wall, and T-wave and standard S1 Q3 T3 inversion.

**Chest X-ray:** Chest X-ray is a common test, but it has low sensitivity and specificity. However, it can detect atelectasis or parenchymal abnormalities (18-69%), pleural effusion (47%), and cardiomegaly (> 50%). Peripheral wedge-shaped opacity in peripheral lung regions and abrupt cut-off of pulmonary arterioles with distal hypoperfusion are rare but are highly suspected of PTE. Chest X-ray may be normal in 12-22% of patients with PTE.

**Angio-CT with protocol for PTE:** for most patients with PTE, it is the selected diagnostic test due to its high sensitivity (> 90%) and specificity (> 95%) for PTE, especially when associated with D-dimer dosage,471 in patients with moderate to high probability. Demonstration of filling failure in any branch of the pulmonary artery using contrast is a diagnosis of PTE.

**Ventilation/perfusion (V/Q) scintigraphy:** this test is reserved for patients with suspected PTE when Angio-CT is contraindicated (renal insufficiency, creatinine clearance < 60 mL/min/m^2^, contrast allergy, or morbid obesity) or when Angio-CT is inconclusive or negative, but in disagreement with the high clinical suspicion. V/Q scintigraphy is a sensitive test for the diagnosis of PTE, but it is not specific due to its high incidence of false-positive results. Accuracy is higher when chest X-ray is normal. V/Q scintigraphy is selected for the diagnosis of PTE during pregnancy. A high probability V/Q scintigraphy is sufficient for the diagnosis of PTE, whereas a normal scintigraphy is sufficient to exclude PTE. Low or intermediate probabilities are not sufficient for diagnosis.

**Pulmonary digital angiography with iodinated contrast:** this test was the historical gold standard for the diagnosis of PTE. With the development of Angio-CT, it is reserved for patients with suspected PTE when Angio-CT or V/Q scintigraphy is not conclusive. In a retrospective analysis of 20 cases of the PIOPED II study,472 digital angiography was shown to be less sensitive than Angio-CT for the diagnosis of small emboli. It has morbidity of 5% and mortality of < 2%. Exposure to radiation is greater than in Angio-CT. Demonstration of filling failure and abrupt cut-off of pulmonary arterial vessel are diagnoses of embolization.

**Pulmonary angiography by nuclear magnetic resonance:** this test is not very sensitive (77-84%). It is reserved for cases in which other methods cannot be performed.

**Echocardiogram:** this test does not diagnose PTE. However, presumptive diagnosis can be performed using the echocardiogram in patients with high clinical suspicion and hemodynamic instability. Approximately 30-40% of patients with PTE have echocardiographic changes, indicative of right ventricular overload, especially in those with massive PTE, such as right ventricular dilatation, right ventricular dysfunction, and tricuspid insufficiency.

#### II. Treatment of Venous Thromboembolism

There is limited evidence for the best treatment for venous thromboembolism in the perioperative period. This is because surgeries may have different bleeding risks, clinical situations may be extremely heterogeneous, and conventional therapy may not be the most appropriate after a specific surgical procedure. Thus, we will present the usual recommended treatment, regardless of the perioperative context, but it is important to customize the decisions in conjunction with the surgeon.

The main pillar of venous thromboembolism treatment (deep vein thrombosis and PTE) is anticoagulant therapy, which should be a long-term therapy, with a duration of at least 3 months.^380^ However, there are clinical situations that require the use of anticoagulant therapy for longer periods, which has become known as extended anticoagulant therapy and implies its use for an indefinite period.^380^

#### II. A. Selection of Anticoagulant

Several recent studies have examined the efficacy of new anticoagulants in acute and long-term treatment of VTE and compared to warfarin. These studies showed that the reduction in the risk of recurrence of VTE is similar with both therapies, including cancer patients.^473-477^ The reduction in the risk of recurrence of VTE with the different new anticoagulants (dabigatran, rivaroxaban, apixaban, and edoxaban) is not directly compared among them, but it appeared to be equally effective based on indirect comparisons.^477^ In fact, in the recommendations, the order of the new anticoagulants cited in the text refers to the chronology of the publication of the phase III studies on VTE and is not the order of preference.

The new anticoagulants have lower bleeding rates and provide greater convenience for patients and health professionals in relation to fixed dose. They have less drug and food interactions and do not require serial blood tests to ensure a specific therapeutic range. Given these advantages, the new anticoagulants are now preferred compared to warfarin for initial and long-term treatments of VTE in patients without cancer.

In cancer patients, a recent randomized study compared the use of LMWH (tinzaparin) and warfarin for the treatment of 900 cancer patients with DVT during the first 6 months. The study demonstrated that LMWH is more effective than warfarin, without alterations in death rates and major bleeding.^478^ Other studies have also shown that the reduction of the risk of recurrence of VTE in cancer patients is higher with the use of LMWH compared to warfarin.^479,480^

Therefore, warfarin is preferentially used in patients with VTE without cancer and LMWH in patients with VTE and cancer. No study has directly compared the new anticoagulants and LMWH in cancer patients. However, based on indirect comparisons, LMWH appears to be more effective than the new anticoagulants in patients with VTE and cancer.^479^

It is important to emphasize that parenteral anticoagulation was given before the use of dabigatran and edoxaban in previous studies. It was not used prior to rivaroxaban and apixaban. It was used before and for a period with warfarin until the desired INR was reached.

#### II. B. Duration of the Anticoagulant Therapy

The studies that determined the appropriate duration of treatment for VTE basically compared four treatment duration options: 4 or 6 weeks; 3 months; more than 3 months, although limited to 6 to 12 months; and extended or indefinite duration therapy. These four treatment duration protocols were tested in different available studies in four profiles of VTE patients with different estimates of recurrence risk after suspending anticoagulant therapy:

(1) VTE caused by surgery (major transient risk factor with 3% recurrence in 5 years);^481^ (2) VTE caused by a transient non-surgical risk factor (estrogen therapy, pregnancy, lower limb lesions, flights > 8 hours, with a risk of recurrence of 15% in 5 years); (3) idiopathic VTE with no transient risk factors or cancer (30% recurrence in 5 years);^482,483^ (4) VTE associated with cancer (15% annual recurrence).^484,485^

Another important factor that guides the duration of anticoagulant therapy in VTE is the risk of bleeding that can be categorized as low (absence of risk factors for bleeding, with a 0.8% annual risk for major bleeding), moderate (one risk factor for bleeding, with a 1.6% annual risk for major bleeding), or high (two or more risk factors for bleeding, with a ≥6.5% annual risk for major bleeding). The risk factors for bleeding during anticoagulant therapy are described in chart 14.^486^

**Chart 14:** Risk factors for bleeding during anticoagulant therapy

Age > 65 years
Age > 75 years
Previous bleeding
Cancer
Metastatic cancer
Renal failure
Hepatic failure
Thrombocytopenia
Previous stroke
Diabetes mellitus
Anemia
Antiplatelet therapy
Poor anticoagulant control
Comorbidity and reduction of functional capacity
Recent surgery
Alcoholism
Non-steroidal anti-inflammatory drugs

It is important to note that for all patients using extended or indefinite anticoagulant therapy, treatment should be reassessed at least annually.

#### II. C. When and How to Prescribe Anticoagulants in Patients With Distal Dvt of The Lower Limbs

It is still unclear whether the benefits of anticoagulation outweigh the risks of anticoagulant treatment for distal isolated DVT because of the low risk of progression and recurrence of VTE.^380^ About 15% of distal isolated DVT will develop with thrombus progression to the popliteal vein and risk of PTE.^487^

The following risk factors favor thrombus extension in distal isolated DVT and support the use of anticoagulant therapy over imaging follow-up: positive D-dimer, extensive thrombosis involving multiple veins, proximal vein thrombosis, absence of reversible trigger factor for DVT, active cancer, previous history of VTE, and hospitalized patient.^488-492^

#### II. D. Role of Catheter-directed Thrombolysis in Deep Venous Thrombosis of the Lower Limb

Evidence is scarce with regard to the use of catheter-directed thrombolysis for the treatment of proximal DVT of the lower limb, causing substantial uncertainty that the benefits outweigh the risks associated with the procedure.^380^

#### II. E. Role of the Inferior Vena Cava Filter

Evidence shows the inconsistent benefit of using the inferior vena cava filter to prevent recurrence of VTE in anticoagulated patients. The most recent randomized study, PREPIC,^493^ demonstrated that inferior vena cava filter implantation during 3 months does not reduce the recurrence of PTE, including fatal PTE, in anticoagulated patients with PTE and DVT, with additional risk factors for recurrence of VTE

#### II. F. Role of Compression Stockings

A recent multicenter, placebo-controlled, study has shown that in contrast to two previous smaller studies, the routine use of compression stockings does not reduce the risk of post-thrombotic syndrome nor does it add any other important benefit.^494^

#### II. G. Subsegmental Pulmonary Thromboembolism Treatment

With technological development of pulmonary angiotomographies, diagnostic identification of subsegmental PTEs increases and the best therapeutic management in these cases is uncertain. Changes are often small and may correspond to false positives, and true subsegmental PTE is generally associated with small DVTs. The risk of progression or recurrence of VTE in the absence of anticoagulation is small in relation to larger PTEs.^486^

Imaging of DVT should be performed on the lower limbs, as well as on the upper limbs and central venous catheters. If DVT is detected, anticoagulant therapy should be introduced, but if no DVT is detected, the need for anticoagulation in these patients is uncertain.^486^

In these cases, it is important to evaluate the risk factors for recurrence or progression of VTE, which include the following: patients hospitalized or with reduced mobility for other reasons; patients with active cancer, especially those with metastatic disease or those treated with chemotherapy; or patients with non-reversible risk factors, such as recent surgery. Similarly, important clinical symptoms that cannot be attributed to another cause or a low functional reserve favor the use of anticoagulant therapy, whereas the presence of a high risk of bleeding favors the preference for clinical follow-up.

#### II. H. Home Treatment of Pulmonary Thromboembolism

Recent meta-analyses evaluated the possibility and safety of home treatment for pulmonary embolism.^495-497^ It is recommended that patients candidate for home treatment meet all the following criteria:^380^ clinically stable with good cardiopulmonary reserve; no contraindications, such as recent bleeding, severe renal or hepatic failure, or severe thrombocytopenia (< 70,000/mm3); willingness to follow the treatment; and feel safe to be treated at home.

#### II. I. Systemic Thrombolysis for Pulmonary Thromboembolism

Systemic thrombolytic therapy is associated with a faster decrease in pulmonary artery pressure, increased arterial oxygenation, and resolution of filling faults on CT, accelerating the resolution of PTE. However, such therapy is associated with increased bleeding risks. Patients who will benefit the most are those who have the highest risk of death associated with PTE and the lowest risk of bleeding.^380^

Recently, three randomized trials evaluated the use of systemic thrombolytic therapy in 1,200 patients with acute PTE and improved the evidence regarding this topic.^498-500^

**It is important to note that patients recently submitted to a surgery will always have an at least moderate risk of bleeding** (Chart 14) and the possibility of using systemic thrombolytic therapy for the treatment of acute PTE needs to carefully assess the risks and benefits and should be discussed with the surgeon.

## 9. Evaluation of Comorbidities

### A) Diabetes Mellitus

#### I. Preoperative

DM affects 6.2% of the Brazilian adult population,^501^ with a progressive increase in prevalence according to age, affecting more than 19% of individuals more than 65 years. These patients have a high incidence of CDs. Glycemic control is one of the most important aspects to be considered in the perioperative evaluation of patients with DM. There is substantial observational evidence that links hyperglycemia to unfavorable surgical outcomes, such as infection, longer hospital stay, disability after discharge, and mortality.

In Brazil, approximately 90% and 73% of type 1 and type 2 DM patients, respectively, are outside the recommended targets for glycemic control (glycated hemoglobin lower than 7.0%). Therefore, it is expected that most individuals in preoperative evaluation need specific guidelines regarding glycemic control.

Preoperative evaluation becomes an additional opportunity to adjust medication doses, educate an individual, and improve metabolic control. A staggered scheme (insulin to correct capillary glycemia) should be avoided as an exclusive therapy for prolonged periods because it is ineffective for most patients. In addition, this scheme favors glycemic variability, attempting to correct the “problem” (hyperglycemia) after it has already occurred and may even be deleterious, predisposing to diabetic ketoacidosis in patients with type 1 DM.

#### Specific Glossary

- **Prandial insulin:** dose of fast (regular) or ultrafast (lispro, aspart, glulisine) insulin used to control postprandial blood glucose, used before meals.
- **Basal insulin:** dose of intermediate (NPH) or slow (detemir, glargine, and degludec) insulin used to control glucose during fasting and interprandial periods. Used in several schemes: fasting, sleeping, and pre-meals, divided into 1 to 2 doses per day (detemir and glargine) and 1 to 4 doses per day (NPH).
- **Correction or supplemental insulin:** dose of fast (regular) or ultrafast (lispro, aspart, glulisine) insulin used to treat hyperglycemia that occurs before or between meals or when the patient is fasting (Table 11).
  **Table 11:** Staggered scheme suggested during fasting

Capillary glycemia (mg/dL)	Scheme suggested
160 to 180 mg/dL	01 IU
181 to 200 mg/dL	02 IU
201 to 250 mg/dL	03 IU
251 to 300 mg/dL	04 IU
Above 300 mg/dL	Intravenous insulin pump or postpone elective surgery until better control
Below 100 mg/dL	Install glucose intake at 5 to 10 g/h*
Below 70 mg/dL	60 mL bolus of intravenous 25.0% hypertonic glucose, install glucose intake at 10 g/h, repeat capillary blood glucose test every 15 minutes until the blood glucose is higher than 80 mg/dL.

* Example: 100 m/h SG at 5.0%.
- **Staggered scheme:** known as “insulin on demand”, “insulin according to dextro or HGT”. Fast (regular) or ultrafast (lispro, aspart, glulisine) dose scheme according to capillary glycemia to treat hyperglycemia.
- **Basal scheme:** use of intermediate or slow insulin alone.
- **Basal-bolus or basal-prandial scheme:** use of combined basal and prandial insulins.

A free Brazilian application was developed at the Hospital das Clínicas (HCFMUSP) to assist physicians and nurses in performing intensive glycemic control in hospitalized patients. InsulinApp is a tool developed for smartphones and tablets that calculates hospital doses of insulin necessary for a patient in a few minutes.508 It is available free of charge from both Google Play (Android) and Apple (iOS) under the name InsulinAPP.

#### Special Considerations for Patients with Type 1 Diabetes Mellitus

Pre-assessment and in-hospital monitoring with specialist is recommended, if available.

Monitor capillary glycemia: pre-meal and at 10 pm while maintaining usual diet; every 4 hours during the fast; and every hour or two hours if using continuous intravenous insulinization.

Never substitute basal-bolus insulin in the preoperative period by staggered scheme alone - risk of diabetic ketoacidosis.

In medium to major surgeries or with a surgical time of more than 1 hour, ideally use continuous intravenous insulin pump as soon as fasting starts or on the morning of surgery, maintaining the therapy during the intraoperative and postoperative periods.

If venous insulinization is not possible to perform, the following can be used:

- Maintain the insulin the evening before surgery.
- In the morning of the day of surgery - reduce basal insulin as described in chart 16.

**Chart 16:** Insulin management in the preoperative period

Insulina	Orientações
NPH	Maintain the dose of the previous day, including the evening dose In the morning of the surgery: If surgery is performed in the early morning: give 2/3 of the dose If surgery is performed in the morning: 1/2 of the dose If surgery is performed in the afternoon: 1/3 of the dose
Detemir, glargine, degludec	Maintain the dose of the previous day Reduce to half on the day of surgery
Fast or ultrafast	Suspend fixed prandial doses Maintain staggered scheme during fasting
- Remove prandial insulin, maintaining basal insulin, capillary glycemia every 3 or 4 hours, and start staggered scheme (prefer ultrafast insulin).
- Install glucose intake the morning of the surgery (before breakfast time). - maintain intake from 5 to 10 g/h. The number of grams per hour depends on the glycemic control.

#### Emergency Surgery in Patients with Diabetes Mellitus

Evaluate blood glucose before surgery.

Correct hypoglycemia and maintain glucose supply at 5 to 10 g/h of glucose. Preferably, control hyperglycemia with intravenous insulin and maintain blood glucose levels between 100 and 180 mg/dL.

Attention to potassium correction.

#### II. Postoperative

In 2001, an important study demonstrated a clinical benefit of strict glycemic control in the postoperative period for the first time in surgical patients: lower rates of in-hospital mortality, polyneuropathy, infections, and acute renal failure, and shorter time of mechanical ventilation and of stay in ICUs.^509^ Regarding patients with diabetes, the clinical benefit associated with strict glycemic control was also observed, but there was no impact on the reduction of mortality. Based on this study, the recommendation was strict glycemic control in the postoperative period for patients undergoing noncardiac surgery.

Another large randomized multicenter trial (NICE-SUGAR)^510^ involving more than 6,000 patients, with approximately one-third of surgical patients and two-thirds of clinical patients, compared strict glycemic control (81-108 mg/dL) with conventional glycemic control (144-180 mg/dL). Surprisingly, the group of patients randomized to strict control presented higher mortality rates in a period of 90 days (27.5%) compared to the conventional group (24.9%). No differences were found in other minor outcomes between the groups. The group with the strict glycemic control presented higher hypoglycemia levels (<40 mg/dL) compared to the control group.

### B) Thyroid Diseases

Hormonal disorders may be responsible for considerable perioperative morbidity and mortality,513 in addition to technical difficulties in managing the airways of patients with goiter.

Tetraiodothyronine (T4) represents 80-90% of thyroid hormone production, and 40% is peripherally converted to triiodothyronine (T3), which is five times more potent. About 50% of T4 is converted to 3,5-triiodothyronine (reverse T3), which has no biological activity. Only 0.2% of T3 and 0.3% of T4 circulate in the free and biologically active form. The rest binds to plasma proteins (albumin, pre-albumin, thyroglobulin). T3 and T3r are converted in the liver, kidneys, and central nervous system into inactive compounds. Severe systemic diseases, trauma, and drugs can block the peripheral conversion of T4 to T3, leading to euthyroid syndrome of the critical patient, which represents a physiological mechanism to save energy in critical situations.

During thyroid surgery, specific complications may occur in the perioperative period. Patients with large goiters may present complications in intubation and extubation (up to 35% have some of airway obstruction), recurrent laryngeal lesion, tracheomalacia, and glottal edema. Hypocalcemia may occur up to 36 hours after thyroidectomy in 20% of cases. Only 3% are permanently hypocalcemic, and calcium must be replaced intravenously at this stage.

#### I. Hypothyroidism

In epidemiological studies, the overall incidence of hypothyroidism varies from 0.1 to 2%. The prevalence of subclinical hypothyroidism is higher, ranging from 4 to 10% in the adult population and tending to be higher in women more than 65 years. Most of the population, even asymptomatic, has thyroid alterations. Some clinical conditions present a potential risk for the development of perioperative complications and a rapid decline in thyroid function, such as age > 65 years; hypothalamic or pituitary disease; coexisting autoimmune disease; irradiation of the neck, thyroid surgery, or radioiodine therapy; significant hyperlipidemia; hyponatremia; high levels of muscle enzyme; macrocytic anemia; and pleural or pericardial effusion.

If these risk conditions are present, screening for thyroid disease may be useful in the preoperative period. The recommended test is TSH because 95% of the causes of hypothyroidism are of primary thyroid etiology.

No randomized study has demonstrated the benefit of patients with hypothyroidism being euthyroid in the preoperative period compared to hypothyroid and postoperative morbidity and mortality. Current evidence shows that if there is a prior diagnosis and time, the patient should be euthyroid in the preoperative period. However, if the patient has subclinical or mild hypothyroidism and the operation is urgent, the surgical procedure should not be postponed. In elective surgeries, treatment can begin, but we should not wait until TSH normalized.

Patients with clinical or moderate hypothyroidism and scheduled for urgent surgery should undergo the surgical procedure and initiate treatment in the immediate postoperative period. Patients with moderate hypothyroidism and elective surgeries should wait for euthyroidism to undergo the surgery. These patients do not necessarily need to achieve normalized TSH levels because 10 to 20% have a slow TSH decay. The most important criterion is that progressive increase and normalization of free T4 levels occur, which should exist within seven days, or treatment should continue to further increase the levels.

Patients with severe hypothyroidism or myxedema coma should only be operated if the surgery is an emergency. If the surgery is elective, previous treatment of hypothyroidism and acquisition of normal thyroid function should be considered. Treatment should be given in the form of T4 and T3. The doses used are as follows: T4 with an attack dose of 200-300 mcg intravenously, followed by 50 mcg per day; T3 dose of 5-20 mcg intravenously, followed by 2.5-10 mcg every 8 hours, depending on age and cardiovascular comorbidities.

In the postoperative period of any patient with hypothyroidism, if the patient does not resume eating in 5-7 days, 80% of the total dose of T4 should be given intravenously or intramuscularly once a day. The dose is 20% lower due to bioavailability.

#### II. Hyperthyroidism

Thyrotoxicosis affects 2% of women and 0.2% of men. The prevalence of clinical and subclinical hyperthyroidism in the USA is 0.2 and 1%, respectively. The most common causes are Graves-Basedow’s disease, toxic nodular goiter, thyroiditis, and iatrogenic. Adrenergic effects pose a high risk for perioperative complications, such as cardiac arrhythmias (8-15% AF). These are related to the increase in the number and/or sensitivity of β-adrenergic receptors. In addition, studies showed that more pronounced hyperthyroidism indicates greater chance of AF. The mortality in hyperthyroidism is related to the occurrence of cardiovascular events^.^^513,517-521^

For the diagnosis, there should be laboratory confirmation of clinical suspicion. TSH level should be low, and free T4 level should be normal (subclinical hyperthyroidism) or high. Several situations may increase the total T4 levels by increasing the T4-binding protein. However, they do not affect free T4, which has biological activity: pregnancy, cirrhosis, acromegaly, Cushing’s syndrome, use of lithium, contraceptives, propranolol, amiodarone, and iodinated contrast agents. In these situations, there is no real hyperthyroidism, only a compensatory increase in free T4, a consequence of the increase in TBG, a T4-binding protein.

#### II. A. Clinical Manifestations in Hyperthyroid Patients with Perioperative Consequences

- **Cardiovascular:** increased cardiac inotropism and chronotropism with decreased systemic vascular resistance (SVR), left ventricular hypertrophy, increased incidence of angina, HF, arrhythmias, and embolic events.
- **Hematologic:** anemia, thrombocytopenia, neutropenia, increase of factor III, decrease of vitamin K-dependent factors, bleeding.
- **Gastrointestinal:** inadequate absorption of drugs.
- **Metabolic/renal:** hypercalcemia, hypoalbuminemia, ketoacidosis, increased drug clearance.
- Pulmonary: myopathy with ventilatory dysfunction.
- Endocrine: increased production and use of cortisol, glucose intolerance, weight loss, and protein catabolism.

#### II. D. Treatment of Thyrotoxic Storm

Treatment of thyrotoxic storm includes hydration, cooling, inotropes (if necessary), administration of PTU attack dose (1,000 mg oral) and maintenance (200 mg every 6 hours), ventilatory support, oral metabolic control, hydrocortisone attack dose of 300 mg intravenously and maintenance of 100 mg every 8 hours, iodine as oral Lugol or intravenous iodine at a dose of 1 g every 8 hours, and, if necessary, plasmapheresis, dialysis, or cholestyramine to remove hormones from the circulation.

### C) Adrenal Insufficiency

Increased level of cortisol during acute stress is an important protective response. However, the metabolic stress caused by surgery can trigger acute adrenal insufficiency (AAI) in individuals with clinical and subclinical disorders. AAI affects the hypothalamic-pituitary-adrenal axis, and the results can be catastrophic, leading to multiple complications and even patient death.

Physical stress increases adrenocorticotropic hormone (ACTH) and cortisol secretion. The increase in cortisol, noradrenaline, and adrenaline levels characterize the stress-induced hormonal changes, minimal in small surgical stress and progressively higher in moderate and severe stress, lasting no more than 24 hours in uncomplicated interventions. The intraoperative period and mainly the anesthetic recovery and extubation periods are the major determinants for axis activation, with increases in plasma cortisol levels, which return to basal values in 24 to 48 hours.^522^ With the increasing demand for endogenous corticosteroids, individuals with impaired function and compromised adrenal reserve may have AAI. Thus, early identification of these individuals for adequate perioperative planning is essential to avoid complications.

#### I. Clinical Conditions of Primary Adrenal Insufficiency

Hypotension and hemodynamic shock (which may be resistant to vasopressors), with multiple organ dysfunction; hypoglycemia; tachycardia; hydroelectrolytic disorders: hyponatremia, hyperkalemia (in primary adrenal insufficiency - AI), hypercalcemia, acidosis; cardiac hypocontractility; anemia, eosinophilia, and neutropenia; nausea, vomiting, weakness, orthostatic hypotension, dehydration, abdominal or flank pain (acute adrenal hemorrhage), fatigue, weight loss; vitiligo, alteration of skin pigmentation, hypogonadism, hypothyroidism.

Diagnosis of AF should not be trusted if there is unexplained hypotension or refractory shock to volume and drugs in the intraoperative or postoperative period, discrepancy between disease severity and patient condition, high fever without apparent cause (negative cultures) or unresponsive to antibiotic therapy, unexplained mental changes, apathy, or depression without specific psychiatric disorder. In such cases, AAI should be initiated, and subsequent diagnostic confirmation should be obtained.

#### II. Identification of Patients at Risk for Adrenal Insufficiency

Patients with diagnosis of AI,^523^ patients at risk for AI^524^ and patients with relative hypoadrenalism (limited adrenocortical reserve): pituitary tumors (macroadenomas); radiation therapy of the pituitary region; previous pituitary surgical intervention; postoperative period of Cushing’s disease surgery, bilateral adrenalectomy or unilateral adrenalectomy in case of another adrenal attack; chronic corticosteroid users (> 5 mg of prednisone or equivalent for more than 21 days or dose > 7.5 mg for more than 14 days); patients with type 1 DM or autoimmune diseases (Hashimoto’s thyroiditis, ovarian or primary testicular failure, hypoparathyroidism, vitiligo, autoimmune polyglandular syndrome); individuals with suggestive clinical conditions (darkening of the skin, weakness, fatigue, nausea, vomiting, depression, hypotension, electrolytic imbalances, hypoglycemia, fever).

Evaluation of the hypothalamic-pituitary-adrenal axis for confirmation of AI should be made by measuring serum cortisol level at 8 AM. Perioperative corticosteroid replacement may be indicated depending on the result (Chart 17). If < 5 mcg/dL, replacement should be performed; 5-10 mcg/dL, perform simple cortrosyn test and measure serum ACTH levels to complement evaluation because it can be a false positive, that is, have an acute response and have no reserve. In this case, empirical therapy with steroids should be started. In cortisol level > 10 mcg /dL, replacement is not necessary.

**Chart 17:** Candidates for perioperative corticosteroid replacement

Use of > 20 mg/day prednisone or equivalent for any length of time
Cushing's syndrome clinic
Use of prednisone > 5 mg for more than 21 days in the last 6-12 months
Use of prednisone ≤ 5 mg given in the afternoon, regardless of the circadian rhythm
Inhaled budesonide
Maximum inhaled corticosteroid dose in children
Potent topical corticosteroid, use on face and genitalia, extensive areas
Treatment with occlusion and skin barrier changes, e.g., psoriasis
Cushingoid appearance, as fragile skin, bruises, hump, hypertension, telangiectasias, full moon face

#### IV. D. Treatment of AI Based on the Extent of the Surgery

- **Small (local anesthesia or hernia):** maintain usual dose of corticoid in the morning without a new attack dose; maintain regular dose for 24 hours perioperatively.
- **Medium (total hip prosthesis):** maintain regular dose of corticoid + 50 mg of hydrocortisone in bolus at induction; maintain regular dose for 24 hours perioperatively.
- **Large (colectomy, esophagectomy, peripheral revascularization, pancreatectomy):** maintain regular dose of corticoid + 100 mg of hydrocortisone in bolus at induction; maintain 50 mg of hydrocortisone 8/8h in the 24-hour perioperative period.

### D) Obesity

Obesity has reached pandemic proportions. In Brazil, more than half of the population is overweight. According to the Risk and Protection Factors for Chronic Diseases by Telephone Survey (VIGITEL), 52.2% Brazilians are overweight.^528^ Approximately 30% of surgical patients are obese.

Obesity is related to several morbidities that influence perioperative evaluation and management, such as atherosclerotic disease, HF, systemic arterial hypertension, PH, DVT, and low functional capacity. Excess weight is also associated with problems in the respiratory system, such as reduced functional residual capacity, atelectasis, and pulmonary shunts. The association results in a risk of rapid desaturation due to the combination of high basal metabolic rate and oxygen demand. Furthermore, sleep disorders, such as obstructive apnea and alveolar hypoventilation, are special concerns in the perioperative period of the obese.

Weight is not only related to the greater risk of complications but also to the distribution of fat mass. Centripetal fat distribution (trunk and abdomen) is associated with metabolic syndrome, sleep disturbances, and unfavorable anatomy for intubation. Classifying the degree and type of obesity and screening for sleep-disordered breathing are essential steps to identify specific functional limitations and guide perioperative decisions.

The World Health Organization classifies obesity in grades: obesity grade 1: BMI 30-34.9 kg/m^2^; obesity grade 2: BMI 35-39.9 kg/m^2^; obesity grade 3: BMI ≥ 40 kg/m^2^. Classifications used in bariatric surgeries still categorize obesity in grades 4 and 5 when BMI exceeds 50 and 60 kg/m^2^, respectively.

The STOP-Bang questionnaire (Chart 18)^529,530^ is a validated tool to track sleep disorders in the preoperative evaluation of obese individuals. Scores from 5 to 8 identify patients with a high probability of moderate to severe obstructive sleep apnea.

**Chart 18:** Screening questionnaire for sleep-disordered breathing (STOP-BANG)

Snoring	Do you snore loudly? (louder than speaking or loud enough to be heard through closed doors)?
Tired	Do you feel tired or drowsy during the day?
Observed	Has anyone ever noticed that you stop breathing while you sleep?
Blood Pressure	Do you treat high blood pressure?
BMI	BMI > 35 kg/m^2^
Age	Age > 50 years
Neck	Cervical circumference above 40 cm
Gender	Male gender

BMI: body mass index.

#### I. Peculiarities in the Evaluation of Surgical Risk in Obese Patients^217,531^

Clinical history limited by the difficulty to differentiate between dyspnea and cardiogenic and pulmonary origins of the low functional capacity of the obese. Physical examination and detailed analysis of the cardiopulmonary system is limited due to obesity. Few risk scores used in the perioperative evaluation include obesity and quantify the risk associated with this variable.

Higher prevalence of comorbidities that are risk factors for atherosclerosis and myocardial ischemia (hypertension, DM, and dyslipidemia); increased risk of thromboembolic events and infection of the surgical wound; greater difficulty in measuring blood pressure and acquiring venous access; longer mechanical ventilation time and longer hospitalization time; increased risk of renal failure; greater sensitivity to opioids and sedatives; increased risk of aspiration of gastric contents; higher probability of presenting hypoxemia due to hypoventilation, pulmonary restriction, postoperative atelectasis, increased occurrence of central and obstructive sleep apnea and hypercapnia; higher mortality in intensive care in severely obese patients. Specific regimens for venous thromboprophylaxis in obese patients are shown in table 12.

**Table 12:** Dosage scheme for prophylaxis of deep venous thrombosis

	50-100 kg	100-150 kg	> 150 kg
Enoxaparin	40 mg 1 x day	40 mg 2 x day	60 mg 2 x day
Dalteparin	5,000 IU 1 x day	5,000 IU 2 x day	7,500 IU 2 x day

### E) Hematologic Diseases

#### I. Anemias

As doenças hematológicas podem aumentar a morbidade e aHematologic diseases may increase the morbidity and mortality of individuals submitted to surgical procedures. Anemia is the most common hematological problem found in the preoperative period. It is generally defined according to WHO criteria:^540,541^ hemoglobin concentration < 13 g/dL for men and < 12 g/dL for women. It is often a sign of underlying disease that can affect the surgical outcome. Studies involving many patients indicated that preoperative anemia is an independent risk factor for morbidity, mortality, and transfusion requirement, with an association between extent of anemia and outcome.^542-546^ Anemia leads to overload of the cardiovascular system, increasing cardiac output. Individuals with CD have less tolerance to anemia, and its presence can intensify the conditions of myocardial ischemia and underlying HF. Therefore, identification of anemia in the preoperative period assists in the identification of patients at risk of adverse outcome in the postoperative period. Whenever possible, anemia should be identified, investigated, and corrected before surgery, although there is no randomized evidence that its correction alters the perioperative risk. On the other hand, there is randomized evidence that the correction of anemia in the preoperative period decreases the need for blood transfusion and consequently the postoperative transfusion risk.^546^

The available guidelines for perioperative blood transfusion are limited, but the risks and benefits of this measure should always be questioned.^547^ Traditional practices, such as correction of preoperative anemia for normal or near-normal values of hemoglobin concentration (Hb ≥ 12 g/dL) to prepare patients for surgery, are not supported in the literature and are not recommended in clinical practice.^546^

Numerous studies and reviews have attempted to establish transfusion triggers for patients with anemia by evaluating two strategies: “restrictive” (usually Hb < 7.0 g/dL) and “liberal” (usually Hb ≥ 7.0 g/dL).^548-551^ Most meta-analyses included perioperative patients of various natures: critical and clinical patients, as well as adults and children. The meta-analysis of Carson et al.^548^ included 6,264 patients. These surgical and clinical patients involved adults and children. The authors concluded that the existing evidence supported the use of restrictive transfusion therapy in most patients, but that the effects of the restrictive strategy in high-risk groups, such as acute coronary syndrome, needed to be tested in future large studies. In the meta-analysis of Holst et al.,^549^ 9,813 patients from 31 randomized controlled trials were included. These trials consisted of twenty perioperative and acute blood loss studies, eight critical patient studies, two trauma studies, and one study with patients with leukemia undergoing bone marrow transplantation. According to the authors, the results were not affected by the inclusion of studies with high risk or unclear risk. The restrictive strategy was associated with the reduction in the number of transfused red cell concentrate units and the number of transfused patients; however, mortality, overall morbidity, and myocardial infarction remained unchanged. The restrictive strategy is safe in most clinical settings, and liberal transfusion does not show any benefit to the patients analyzed in this review.^549^

In the meta-analysis of Docherty et al.,^551^ they specifically investigated the effect of restrictive versus liberal strategy in patients with CD undergoing noncardiac surgery. A total of 3,033 patients were included: 1,514 with restrictive transfusion and 1,519 with liberal transfusion. The risk of acute coronary syndrome was higher in patients with a restrictive strategy compared to patients with a liberal strategy, but the effects on mortality and other outcomes were uncertain in a period of 30 days. The authors concluded that it may not be safe to use a transfusion trigger below 8 g/dL in these patients.^551^

Perioperative mortality in a period of 90 days was investigated in a recent review with meta-analysis, which included only perioperative adult patients and critically ill patients. Twenty-seven studies with 11,021 patients were included in the review: 17 in the perioperative period (9 in orthopedic surgery, 5 in cardiac, 1 in vascular, 1 in oncologic, and 1 in obstetric) and 10 in critically ill patients. Overall, there was no difference in mortality between the liberal and restrictive strategies. However, in the perioperative period, mortality was reduced in adult patients randomized to receive the liberal strategy compared to those who received the restrictive strategy with 7,552 patients. In critical patients, there was no difference between the groups. The heterogeneity between the studies was low. It was concluded that blood transfusion had a different statistically significant effect on the survival of patients in different clinical contexts.^550^

In an isolated study, Carson et al.^552^ included 2,016 patients aged ≥ 50 years. The patients had a history or risk factors for CD, with a hemoglobin concentration below 10 g/dL after hip surgery. They were randomized to liberal (transfusion trigger above 10 g/dL) or restrictive (symptoms of anemia or Hb < 8 g/dL) strategy to determine whether a higher transfusion trigger would improve the recovery of patients submitted to orthopedic hip fracture surgery. The authors concluded that the liberal and restrictive strategies did not reduce death rates, did not improve recovery in a period of 60 days of follow-up, and did not reduce hospital morbidity in older patients at high cardiovascular risk.^552^

Another isolated study was conducted at John Hopkins Hospital including 10,163 patients submitted to vascular or gastrointestinal cardiothoracic surgery. The authors aimed to determine the transfusion practices and the effect of the use of transfusion on the perioperative outcome. They concluded that the use of the liberal transfusion trigger (Hb ≥ 7.0 g/dL) after major surgeries was more common than the restrictive practice (trigger < 7.0 g/dL) and that patients with restrictive transfusion had no increased risk of complications compared to patients with liberal transfusion.^553^

Therefore, the optimal transfusion trigger remains undetermined. Hemoglobin level is probably not the answer because some patients require higher values and others tolerate values lower than 7 g/dL.^554^ The transfusion triggers used in isolated studies and in studies that were part of large reviews with meta-analysis are not homogeneous.

Therefore, the decision on blood transfusion should be based not only on hemoglobin levels, but also on the suspicion of organic ischemia, the risk or presence of bleeding, the status of the intravascular volume, and the susceptibility to complications due to inadequate oxygenation.^555^ Common sense, careful observation of the patient, and clinical context should guide the decision of the best strategy for each case. It should be noted that blood transfusion is not a risk-free procedure and that a dose-dependent relationship exists between transfusions and complications.^545^ Thus, even if selecting the liberal strategy, hemoglobin correction towards normal values is not necessary. One unit of erythrocyte concentrate increases the hemoglobin rate by approximately 1.0 g/dL and the hematocrit by approximately 3.0%. The optimal rate of administration of red blood cell concentrate should consider the clinical situation. Most patients can receive a packed red blood cell unit every one to two hours. Patients at risk of volume overload should receive 1.0 mL/kg/h. After transfusion of each unit, the patient should be reevaluated and the hemoglobin level must be determined.^547^

#### II. Thrombocytopenia

Several studies have demonstrated a strong correlation between thrombocytopenia and hemorrhagic risk, as well as the effectiveness of platelet transfusion in reducing this risk. However, controversy still exists about the appropriate value to indicate for transfusion of platelet concentrates.^562^ Patients scheduled for invasive surgical procedures may present benefits with a higher platelet count with 50,000 platelets/mm3.^562-564^ In neurosurgeries, there is no evidence-based data to determine a safe minimum platelet count, with several consensuses indicating 100,000 platelets/mm3.^565-567^

#### III. Hereditary Antiphospholipid Antibodies and Thrombophilias

Antiphospholipid antibodies are a family of autoantibodies directed against plasma phospholipid-binding proteins.^569^ Antiphospholipid syndrome is characterized by thrombosis (arterial and/or venous) and/or gestational morbidity in patients with persistent antiphospholipid antibodies.^570^

However, there are patients with a persistent presence of antiphospholipid antibodies without vaso-occlusive manifestations, only gestational morbidity and manifestations are not considered as criteria for the antiphospholipid syndrome (thrombocytopenia, livedo reticularis, cardiac valve disease).^569^ In addition, not every positive test for antiphospholipid antibodies is clinically significant and not every patient with positive antiphospholipid antibodies has the same thrombotic risk.^569^ To better estimate the thrombotic risk in patients with positive antiphospholipid antibody tests, some variables should be considered including persistently positive laboratory tests and presence of additional thrombotic risk factors.^569^

Although several studies have evaluated thrombotic risk in asymptomatic patients with persistent antiphospholipid antibodies, most of them included patients with systemic lupus erythematosus. The annual risk of the first thrombotic event in these laboratory-positive individuals, but without other associated autoimmune diseases and other thrombotic risk factors, is low (less than 1% per year). In the presence of another autoimmune disease, the risk increases to less than 4% per year. Based on the hypothesis of the association of “two injuries”, antiphospholipid antibodies induce a prothrombotic and proinflammatory phenotype in endothelial cells that is not capable of causing thrombosis alone. However, the presence of a triggering event or “second injury”, such as infection, surgeries, estrogen use, and prolonged immobilization, may trigger a vaso-occlusive event.^569^

Therefore, pharmacological antithrombotic prophylaxis is associated with mechanical measures in patients with positive antiphospholipid antibodies and in a period of greater vaso-occlusive risk (surgeries, immobilization, hospitalization).^569^ Patients with antiphospholipid syndrome under anticoagulant treatment have a higher thrombotic risk when submitted to surgical procedures.^367^

The term thrombophilia describes the tendency to develop venous thromboembolism due to a state of hypercoagulability caused by the presence of inherited or acquired abnormalities of coagulation or fibrinolysis.^571^ Hereditary thrombophilias do not present the same thrombotic risk. Severe thrombophilias are those resulting from deficiencies of natural anticoagulants (antithrombin, protein C, and protein S), abnormalities in homozygous, and presence of multiple defects. Presence of factor V Leiden in heterozygosity and the mutation G20210A in heterozygosis are considered mild thrombophilias. On the other hand, the presence of family history of venous thromboembolism events is a strong risk factor for venous thromboembolism, regardless of the presence of genetic alterations.^571^ Documentation of the presence of an inherited thrombophilic alteration implies the need for primary anticoagulant prophylaxis in situations where an increased risk of venous thromboembolism exists, such as surgical procedures.^571^

#### IV. Hemophilia A (Factor Viii Deficiency) and B (Factor IX Deficiency)^572^

Surgical procedures should be performed in conjunction with a team experienced in the treatment of hemophilia. Before performing the surgical procedure, ensure that sufficient concentrate of the deficient factor is available.

The procedures must be performed in a medical center with adequate laboratory support, with capacity to monitor the deficient factor. Preoperative laboratory evaluation should always include the search for inhibitors for the deficient factor;

The surgical procedure should be performed at the beginning of the week and at the beginning of the day to provide optimal laboratory and blood bank support. For the intraoperative period, the plasma level of the deficient factor should be corrected for hemostatically safe values through the use of specific factor concentrate.

In the postoperative period, maintain the plasma concentration of the deficient factor for adequate time and concentration according to the type and size of the surgery.

Efficacy of hemostasis should be assessed using the criteria defined by the International Society of Thrombosis and Hemostasis (ISTH).

#### V. Von Willebrand Disease^573,574^

In the postoperative period, the minimum plasma levels of FVIII:C and von Willebrand factor:ristocetin cofactor (vWF:RCo) will vary according to the type and surgical extent.

### F) Renal Failure

Patients with renal failure are more prone to postoperative complications, prolonged hospitalization time, higher costs during hospitalization, and higher mortality than those without renal dysfunction.^45,575-578^ Renal failure or preoperative dialysis has been consistently associated with postoperative complications and high mortality.

In preoperative evaluation, renal function can be assessed using the Cockroft-Gault formula, or glomerular filtration can be estimated using the MDRD equation. Estimated glomerular filtration of less than 60 mL/min /1.73 m^2^ is a risk factor for cardiac and noncardiac complications in the postoperative period and is associated with mortality up to two times higher compared to patients with normal renal function^577,578^ Lee et al.^45^ developed and validated a prognostic model for cardiovascular complications after noncardiac surgeries. The risk factors identified were (increasing risk) history of congestive HF, coronary ischemic disease, high risk surgery (abdominal aortic aneurysm, other vascular, thoracic, abdominal, and orthopedic surgeries), insulin-dependent DM, preoperative creatinine > 2.0 mg/dL, and cerebrovascular disease.

The development of acute renal injury (ARI) is a serious complication in the postoperative period and occurs, depending on the type of surgery, in 1-30% of the cases, with mortality around 50%.^579-581^ There is evidence that small changes in serum creatinine are associated with increased morbidity and mortality in clinical and surgical patients.^582-585^

In the latest international guidelines, ARI is considered when a patient presents an increase of 0.3 mg/dL in serum creatinine in 48 hours or a 50% increase in baseline value within 7 days associated or not with the reduction of urinary volume to values below 0.5 mL/kg/h over a 6-hour period.^586^

In a study with 75,952 noncardiac surgeries, the authors identified the following risk factors for ARI in the postoperative period: age ≥ 56 years, male, emergency surgery, intraperitoneal surgery, DM with oral medication or insulin, decompensated HF, hypertension, “mild” renal failure (preoperative creatinine between 1.2 and 1.9 mg/dL), and “moderate” renal failure (creatinine ≥ 2.0 mg/dL). Patients with 6 or more risk factors had an ARI incidence of 9% in the postoperative period and mortality eight times higher than the patients who did not present renal dysfunction.^587^

The prevention of ARI in the postoperative period depends on the following care: identifying risk factors for its development (mainly preoperative renal failure), avoiding the use of nephrotoxic drugs, maintaining adequate hydration, and avoiding hypotension. Even relatively short periods of intraoperative hypotension (MAP lower than 60 mmHg for more than 20 minutes or 55 mmHg for more than 10 minutes) are associated with an increased risk of ARI.^588^

Attempts to prevent ARI with drugs, such as diuretics and vasoactive amines, have not shown efficacy.^589,590^ Potentially nephrotoxic drugs should be avoided or used appropriately with correction for the level of renal function. Aminoglycoside antibiotics, amphotericin B, radiological contrast, and non-hormonal anti-inflammatories are examples of nephrotoxic drugs commonly used in the perioperative period. The use of anti-inflammatories should be avoided, particularly in patients at risk: advanced age, previous renal failure, HF, dehydration, concomitant use of ACE inhibitors, and diuretics or other nephrotoxic agent.^591,592^ ACE inhibitors and angiotensin II receptor blockers (ARBs) are potentially nephrotoxic drugs, and prescription should be evaluated in the perioperative period. In a recent work with orthopedic patients, use of ACE inhibitors or ARBs is associated with a higher risk of developing ARI in the postoperative period.^593^

In the preoperative evaluation of patients with chronic renal failure on dialysis or renal transplants, some aspects are relevant. Many of these patients have known risk factors for coronary ischemic disease, such as advanced age, systemic arterial hypertension, or DM. Patients on a renal replacement therapy program should undergo dialysis before surgery to prevent hypervolemia, correct electrolyte and acid-base imbalances, and reduce the risk of bleeding due to uremia. In renal transplant patients, immunosuppression should be carefully adjusted by the nephrologist in the perioperative period because of the risk of acute rejection and nephrotoxicity.

The risk of postoperative complications is well defined in patients with renal failure. In these cases, the evaluation of the nephrologist should be considered. It should always be noted that creatinine is a poorly sensitive marker of renal function. Therefore, creatinine < 1.2 mg/dL does not necessarily mean normal renal function, particularly in elderly patients or patients with reduced muscle mass. The preoperative evaluation is an opportunity to communicate with the patient and the clinical-surgical team to define measures to prevent deterioration of renal function and for subsequent follow-up aimed at delaying the progression of chronic renal failure.

### G) Pulmonary Hypertension

PH is a clinical condition that results from increased right ventricular afterload, leading to pressure increase in the pulmonary vascular territory and progressive right ventricular dysfunction.^594,595^ The diagnosis of PH involves right chamber catheterization and measurement of pulmonary artery pressure. Hypertensive condition is defined by a mean pulmonary arterial pressure (PAPm) greater than or equal to 25 mmHg.^596^

There are several mechanisms or clinical conditions that can lead to PH from direct involvement of the pulmonary vasculature to left heart disease, pulmonary parenchymal diseases, or even associated with chronic PTE.^597,598^

The pathophysiology of the decompensation of PH depends on the functional characteristics of the right ventricle. Due to the normal mechanical features of the pulmonary circulation (high complacency and low resistance), there is no tolerance for afterloading increases. The increase of the afterload generates distension of the free wall of the RV and consequent decoupling of the muscular fibers (Frank-Starling’s Law), impairing the efficiency of the RV systole.^594,599,600^

There are several causes of RV failure including natural evolution of PH, regardless of the underlying cause, as well as other decompensations, such as infectious cardiac arrhythmias^601^ and surgical stress.^602^

The surgical procedure is related to significant morbidity and mortality in patients with PH.^603,604^ Mortality is estimated between 4% and 24% in several cases, depending on the stage of the disease and the surgical procedure that the patient underwent.^605^ When only cardiac surgeries are considered, mortality is higher than 25%, because there is a high risk of ischemia and RV dysfunction, particularly at the time of cardiopulmonary bypass.^602^

During surgery, several factors may impair RV function due to reduction of coronary perfusion with consequent ischemia and elevation of pulmonary vascular resistance (PVR), leading to increased afterload (Chart 19).^602^

**Chart 19:** Factors contributing to deterioration of RV function*

Increased sympathetic tone (generates vasoconstriction) – e.g., pain
Hypoxia
Injury of pulmonary reperfusion
Excess volume administered
Positive pressure ventilation
Dysfunction (systolic or diastolic) of the left ventricle
Embolism (gas, thrombus, fat)
Acidosis
Acute respiratory distress syndrome (ARDS)

* Adapted from Galiè N et al.^602^

Ideally, perioperative evaluation of these patients should be performed by a multidisciplinary team. The need for the surgery in question should be carefully evaluated, considering the risks and benefits and avoiding emergency procedures that may increase morbidity and mortality.

Preoperative evaluation is complex because it should include assessment of baseline conditions related to the genesis of PH, as well as the associated hemodynamics. In this manner, evaluation is based on clinical data obtained with chest X-ray, pulmonary function test, ECG, echocardiogram, and measurement of biomarkers, such as BNP.

Performing right cardiac catheterization to effectively assess ventricular function through direct measurement of cardiac output, as well as levels of atrial and ventricular filling pressures, may be required, depending on the clinical condition of the patient and the procedure to be performed. This comprehensive evaluation aims to better control PH, with optimization of diuretic therapy, as well as the use of specific medications in patients with pulmonary arterial hypertension.^602^

Some specific conditions deserve particular attention. Patients with chronic PTE should maintain anticoagulant therapy throughout the perioperative period. The transition from oral anticoagulants to anticoagulants with a known half-life (e.g., enoxaparin or UFH in an infusion pump) is recommended. As for anticoagulated patients, due to pulmonary arterial hypertension, the perioperative suspension of this medication does not imply an additional risk and can be performed aiming at greater procedural safety.^602,603^ In these patients, the use of specific drugs to control PH, such as endothelin receptor antagonists, phosphodiesterase type 5 inhibitors, and prostanoids, should be maintained; if necessary, substitution with inhaled or intravenous formulations is possible.^602,604^

Regional anesthesia (blocks or epidural) is apparently better tolerated in patients with PH than general anesthesia.^606^ In the intraoperative period, care must be taken to manage the fluids administered (avoiding excess or lack of volume, which may deteriorate cardiac output), as well as analgesic control. Hypotension should be avoided to maintain adequate coronary perfusion of the RV. For this, monitoring with invasive blood pressure and pulmonary artery catheter is useful, as well as vasoactive drugs that maintain SVR without significantly interfering with PVR.^602,603^

Mechanical ventilation during the surgical procedure should be protective with low tidal volume (6 mL/kg with steady pressure < 30 cmH_2_O), avoiding hypoxia. It must give preference to the management of FiO_2_ over the increase of PEEP, not to compromise venous return and decrease RV preload, as well as a potential increase in PVR.^602^ It is possible to use inhaled nitric oxide considering its short half-life, low repercussion in systemic hemodynamics, and significant auxiliary role in the control of PVR.^596^

Considering all the complexity related to the right ventricular dysfunction, patients with PH scheduled for noncardiac operations should have the preoperative evaluation and surgical procedure preferably performed in a specialized center for the treatment of various forms of PH.^53^

### H) Asthma and Chronic Obstructive Pulmonary Disease

Perioperative pulmonary complications largely contribute to perioperative morbidity and mortality. Some series estimate that pulmonary complications may be present in up to 70% of cases in the postoperative period, depending on the type of surgery and clinical profile of the patient.^607,608^ In addition, pulmonary complications are among the ones that increase hospitalization costs and lead to longer hospitalization.^608^

The definition of postoperative pulmonary complication greatly varies. The most common are atelectasis, infections (including acute bronchitis and pneumonia), prolonged mechanical ventilation and respiratory failure, exacerbation of chronic lung disease, and bronchospasm.^609,610^

Risk factors related to patients that are associated with a higher incidence of pulmonary complications are diagnosis of COPD, asthma, active smoking, obstructive sleep apnea, PH, and upper airway infection.^607,611-613^ Regarding the factors associated with the procedure, the surgical site should be emphasized (the closer to the diaphragm, the greater the risk of complications),^610^ but also the duration of the procedure (greater risk with surgeries lasting more than 3 to 4 hours),^614^ type of anesthesia (general anesthesia with a greater risk than neuroaxial block)^615^ and type of neuromuscular blocker (pancuronium associated with a higher incidence of posterior neuromuscular block than agents with a less prolonged effect).^616^

There is a significant difference in the evaluation of patients scheduled for pulmonary resection and patients scheduled for other types of surgery. In the first group, pulmonary function tests, arterial blood gas analysis, chest imaging exams, and cardiopulmonary tests are fundamental.^611^ Risk predictors, such as ARISCAT, Arozullah, and Gupta, can be used to estimate perioperative pulmonary complications of surgeries without pulmonary resection.^617-619^

The simplest table is ARISCAT^617^ that predicts the general incidence of postoperative complications (any severity). In this table, independent risk factors receive a weighted score, producing risk ranges for postoperative complications: 0 to 25 points: low risk, 1.6% complication rate; 26 to 44 points: intermediate risk, 13.3% complication rate; and 45 to 123 points: high risk, 42.1% complication rate (Table 13).

**Table 13:** ARISCAT risk score for estimation of postoperative pulmonary complications^617^

Variável	Points
Age	
≤ 50 years	0
51–80 years	3
> 80 years	16
Preoperative SpO2	
96%	0
91–95%	8
≤ 90%	24
Type of surgery	
High abdominal	15
Intrathoracic	24
Duration of surgery	
≤ 2 hours	0
2–3 hours	16
> 3 hours	23
Other risk factors	
Respiratory infection in the last month	17
Preoperative anemia with Hb ≥ 10 g/dL	11
Emergency surgery	8

Regarding management to reduce pulmonary complications, the recommendations are similar to those outside the surgical context aimed at optimizing pulmonary function and minimizing the occurrence of respiratory complications. Optimization of pulmonary function includes the use of antibiotics when active infection is observed, as well as the use of corticosteroids and/or bronchodilators in patients already taking these drugs or who present residual bronchospasm. Smoking cessation should be recommended preferably more than two months before the surgical procedure.

Specialized physiotherapy care and follow-up are important in this context. Education of the patient regarding pulmonary expansion exercises is fundamental since the preoperative period. In a systematic review of the literature conducted in 2016, the approach with postoperative pulmonary expansion exercises was the only strategy with a Level of evidence A for the reduction of pulmonary complications.^620^

In summary, there is no recommendation for specific reduction of perioperative cardiac complications in patients with COPD/Asthma.

### I) Smoking

Smoking is the main avoidable cause of death worldwide. It contributes directly to at least 20% of all deaths, and about 200,000 deaths per year in Brazil. Hospitalizations are an opportunity to sensitize patients to quit smoking, as well as to facilitate monitoring of symptoms of nicotine withdrawal and close follow-up of tolerance and efficacy of treatments eventually established.

Reduction of mortality risks and various postoperative complications in smoking is also focused in the perioperative care management, given the significant effect of smoking on postoperative healing, infection rates, surgical bleeding, pain control, and respiratory, cardiocirculatory, and orthopedic complications, among others. History of smoking is associated with longer stays in ICU in the postoperative period and longer hospitalizations.^621-626^ Despite this, discussion about smoking during the preoperative preparation of the patient is mostly absent, which is partly due to the lack of knowledge of the doctors on the ideal timing for cessation of smoking. Recognizing the right time during a surgical risk assessment to address the issue of smoking and initiating treatment as early as possible can have significant reductions in clinical and surgical complications and lower costs to the health system.

#### I. Cessation of Smoking During Hospitalization

Cessation of smoking during hospitalization offers an opportunity to access withdrawal symptoms more readily, titer medication doses more safely, and monitor the effectiveness of the therapeutic program more reliably.

The reasons that drive patients to stop smoking during hospitalization, which are part of health treatment or merely resulting from the condition of staying in a tobacco-free environment, should be seen as an important step. Measures of support and follow-up indispensable for the patient to remain abstinent should be implemented.

If such efforts are not organized in a structured program that involves the identification of smokers at the time of hospitalization, the medical center responsible for therapeutic interventions (informational, cognitive-behavioral, and medication), follow-up during hospitalization, and post-discharge follow-up, such efforts become ineffective in the medium and long term.

#### II. Cessation of Smoking in the Preoperative Period

The negative effects of smoking on surgical outcomes are multifactorial. However, they are mainly due to the direct effects of nicotine carbon monoxide (CO) and increased oxidative and inflammatory stress. CO and nicotine increase heart rate, blood pressure, and tissue oxygen demand, as well as decrease oxygen transport capacity. Because of the vasoconstricting effect of nicotine, it increases the risk of tissue ischemia in surgery and in other areas, such as the coronary artery.^627^

The irritant and proinflammatory effect of numerous components of cigarette smoke on the airways also increase the susceptibility of smoking patients to respiratory infections, local healing complications in lung surgeries, and longer periods under mechanical ventilation.^628^

Smoking is also associated with the need for higher doses of anesthetics and neuromuscular blockers,^629^ increased incidence of thromboembolic events, and slower repair processes in orthopedic surgeries.^630^

Patients scheduled for surgeries are usually more motivated to quit smoking and are thus susceptible to a therapeutic approach to quit smoking. With the regulation of hospitals (and other enclosed spaces for public and private use) as tobacco-free environments, and with the increasing availability of effective therapeutic resources to help the patient to stop smoking, the preoperative period becomes a perfect time to stop smoking before elective surgical hospitalization.

The ideal time to stop smoking before surgery had been controversial. This was partly due to the great methodological heterogeneity of studies that evaluated the different periods to stop smoking, the difficulty of controlling confounding variables in the samples of patients, the great variation in the postoperative follow-up time, and the multiplicity of outcomes studied.

A review of prospective studies on the impact of smoking cessation in the preoperative period on the occurrence of postoperative complications (respiratory, infectious, general mortality and length of hospital stay) was conducted by Cropley and Theadom.^631^ They concluded that although there is great methodological limitation of the studies evaluated, there are several benefits of smoking cessation before surgical hospitalizations. They reported that longer abstinence period results in greater benefit. It should also be mentioned that there is no ideal period to recommend preoperative smoking abstinence, in terms of reduction of surgical complications and risk in the medium and long term. Cessation of smoking should not be postponed due to the unsustainable assumption that risk increases when cessation occurs less than two months before surgery.

In 2009, a retrospective cohort study evaluated data from 7,990 lung resection surgeries due to neoplasia. The study concluded that the risks of in-hospital mortality and respiratory complications after lung resection were higher in smokers and clearly reduced by smoking cessation in the preoperative period. The ideal interval between cessation of smoking and surgery could not be identified, which reinforced the recommendation for counseling (and treatment) for smoking cessation, regardless of the proximity of the surgery. This corroborates the results presented in the study published in 2001 by Nakagawa et al.^632^ They found a clear and increasing reduction in the risk of postoperative complications after four weeks of preoperative smoking cessation.

#### III. Therapeutic Strategies

As in general situations, the treatment of nicotine dependence in patients scheduled for surgery and in hospitalized patients is based on cognitive-behavioral interventions (brief approach, individual counseling, provision of informational materials, and group therapy), systematized or not, and in the pharmacological support.

Regarding the “intensity” of the non-pharmacological approach, a systematic review published in 2012 evaluated several studies in hospitalized patients.^633^ The study showed a dose-response relationship between the intervention and the cessation rate. Moreover, structured counseling programs initiated at the hospital and extended for at least one month after discharge are more effective compared to single-point approaches during hospitalization (RR 1.37; 95% CI 1.27-1.48; 25 studies).

Prospective studies evaluating the effectiveness of implementing a structured counseling, cognitive-behavioral approach, pharmacologic support, and post-discharge follow-up of hospitalized smokers showed success rates of about 35-44% in six months^634,635^ and approximately 33% after 12 months, with studies showing success rates above 50% after one year in hospitalized coronary patients.^636^

Given the need to specify, in cases of surgical and hospitalized patients, cessation of smoking and the control of nicotine withdrawal symptoms in a shorter period, nicotine replacement therapy (NRT), isolated or combined, is the most frequent approach selected. The usual transdermal nicotine prescription schemes (6 to 8 weeks of 21 mg/24h or 15 mg/16h, 2 weeks of 14 mg/24h or 10 mg/16h and 2 weeks of 7 mg/24h or 5 mg/16h, depending on the presentation selected) are recommended, in association with rapid forms of ad libitum replacement (in Brazil, chewing gum and tablets are available, both in presentations of 2 and 4 mg per unit) for craving episodes. The addition of NRT to an intensive counseling intervention (more than one session, post-discharge) increased smoking cessation rates compared to intensive counseling alone (RR 1.54, 95% CI 1.34-1, 79, six studies).^637^

The same systematic review did not show evidence of benefit of adding bupropion (RR 1.04, 95% CI 0.75-1.45, three studies) or varenicline (RR 1.29, 95% CI 0.95-1.76, two studies) to intensive counseling. Regarding bupropion, new studies, such as the randomized placebo-controlled trial conducted by Eisenberg et al.,^638^ failed to demonstrate the superiority of bupropion over placebo in hospitalized patients. In the same year, a study published by Smith et al.^639^ evaluated the addition of varenicline to counseling through a randomized, placebo-controlled, protocol. The result showed a significant superiority of pharmacological intervention compared to the control group (RR 1.45; 95% CI 1.03-2.03; p = 0.03).

Although there is potential benefit of other pharmacological approaches to smoking cessation in hospitalized patients, NRT remains the standard of care at the same doses and schemes normally recommended for other clinical situations. The use of customized doses (above 21 mg/day) of nicotine replacement to reach plasma levels of nicotine closer to the arterial concentrations of an active smoker and aimed at better control of withdrawal symptoms in heavy smokers has been tested and is safe up to doses higher than 42 mg per day,^640-643^ even in individuals who persisted smoking. However, the heterogeneity of the studies and the small number of volunteers included do not provide sufficient evidence of an increase in long-term smoking abstinence rates.

The possible association of NRT with non-nicotinic drug (such as bupropion) or the choice of varenicline monotherapy is theoretically an acceptable option, but they do not find great support in specific studies in these special situations.^640-643^

NRT is not superior over bupropion in patients with a history of recent (below six weeks) high-risk acute coronary syndrome and patients with complex ventricular arrhythmias. Published studies are controversial in identifying additional benefits (in addition to the control of withdrawal symptoms) from the drug treatment compared to the counseling program and behavioral approach alone.

**Figure 6:**
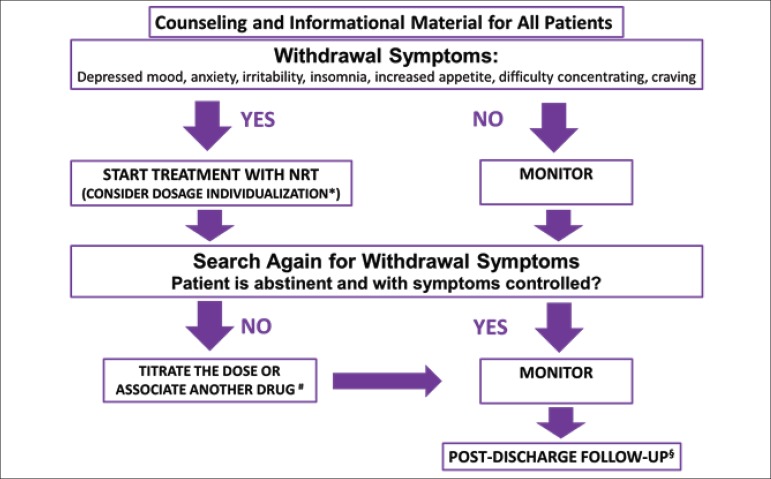
In-hospital patient algorithm. * < 20 CIGARETTES/DAY: 14 mg patches; 20-30 cigarettes/day: 21 mg patches; 31-40 cigarettes/day: 21 mg + 7 mg patches; > 40 cigarettes/day: 21 mg + 14 mg patches. For all cases, consider the association with nicotine gum or tablet of 4 mg ad libitum. † consider titration of transdermal nicotine dose (avoid doses above 42 mg/day) or substitute for varenicline. ‡ outpatient return in a maximum of one month, with follow-up for a time not lesser than one month. Reduce dose of NRT according to the guidelines applicable to general situations.

For hospitalized patients, we propose a treatment based on the flowchart in figure 6.

There is consistent evidence supporting the treatment to stop smoking in subpopulations of hospitalized patients and candidates for surgical procedures. This intervention is extremely effective and inexpensive.

In general, the therapeutic strategies are not much different from the routines suggested for general populations, but there is a preference for NRT.

Hospital admissions and consultations for the evaluation of surgical risks and perioperative care should consider the active approach to cessation of smoking, researching, advising, treating, and following-up these patients.

## Figures and Tables

**Table:** 

Declaration of potential conflict of interest of authors/collaborators of 3^rd^ Guideline for Perioperative Cardiovascular Evaluation of the Brazilian Society of CardiologyIf the last three years the author/developer of the Guidelines:							
Names Members	Participated in clinical studies and / or experimental trials supported by pharmaceutical or equipment related to the guideline in question	Has spoken at events or activities sponsored by industry related to the guideline in question	It was (is) advisory board member or director of a pharmaceutical or equipment	Committees participated in completion of research sponsored by industry	Personal or institutional aid received from industry	Produced scientific papers in journals sponsored by industry	It shares the industry
Alina Coutinho Rodrigues Feitosa	No	No	No	No	No	No	No
André Coelho Marques	No	No	No	No	No	No	No
Bruno Caramelli	No	Servier, Bayer	No	No	No	No	No
Carisi A. Polanczyk	No	No	No	No	No	No	No
Carlos Eduardo Rochitte	Toshiba Medical	No	No	Toshiba Medical	No	Siemens Medical	No
Carlos Jardim	No	No	No	No	No	No	No
Carolina L. Z. Vieira	No	No	No	No	No	No	No
Claudio Pinho	No	No	No	No	No	No	No
Daniela Calderaro	No	No	No	No	Bayer	Bayer	No
Danielle Menosi Gualandro	No	No	No	No	EMS, Servier, Sanofi, Roche, Bayer	Bayer, Servier	No
Débora Yuri Moura Nakamura	No	No	No	No	No	No	No
Denise Iezzi	No	No	No	No	No	No	No
Dirk Schreen	No	No	No	No	No	No	No
Eduardo Leal Adam	No	No	No	No	No	No	No
Elbio Antonio D'Amico	No	Bayer	No	No	Bayer	No	No
Emerson Quintino de Lima	No	No	No	No	No	No	No
Emmanuel de Almeida Burdmann	No	Baxter	No	Fresenius, Baxter, Abbvie	No	No	No
Enrique Indalecio Pachon Mateo	No	No	No	No	No	No	No
Fabiana Goulart Marcondes Braga	No	No	No	No	No	No	No
Fabio S. Machado	No	No	No	No	No	No	No
Flavio J. de Paula	No	No	No	No	No	No	No
Gabriel Assis Lopes do Carmo	No	No	No	No	No	No	No
Gilson Soares Feitosa-Filho	No	No	No	No	No	No	No
Gustavo Faibischew Prado	No	No	No	No	Boehringer Ingelheim, Bristol-Myers Squibb	No	No
Heno Ferreira Lopes	No	No	No	No	No	Novartis, Merck Serono	No
João Ricardo Cordeiro Fernandes	No	No	No	No	No	No	No
José Jayme Galvão de Lima	No	No	No	No	AstraZeneca	No	No
Luciana Sacilotto	No	No	No	No	No	No	No
Luciana Savoy Fornari	No	No	No	No	No	No	No
Luciano F. Drager	No	No	No	No	Philips Respironics	EMS	No
Luciano Janussi Vacanti	No	No	No	No	No	No	No
Luis Eduardo Paim Rohde	No	No	No	No	No	No	No
Luís Felipe Lopes Prada	No	No	No	No	No	No	No
Luis Henrique Wolff Gowdak	No	No	No	No	No	Servier	No
Marcelo Luiz Campos Vieira	No	No	No	No	No	No	No
Maristela Camargo Monachini	No	No	No	No	No	No	No
Milena Frota Macatrão-Costa	No	No	No	No	No	No	No
Milena Ribeiro Paixão	No	No	No	No	No	No	No
Mucio Tavares de Oliveira Jr.	No	No	No	No	Novartis, Merck Serono, Boehringer Ingelheim	Baldacci, Merck Serono, Boehringer Ingelheim, Torrent Pharma, EMS, Novartis	No
Pai Ching Yu	No	No	No	No	No	No	No
Patricia Ramos Cury	No	No	No	No	No	No	No
Paula R. Villaça	No	No	No	No	No	No	No
Pedro Silvio Farsky	No	No	No	No	Pfizer, Bayer, Servier	Pfizer	No
Rinaldo Focaccia Siciliano	No	No	No	No	No	No	No
Roberto Henrique Heinisch	No	No	No	No	No	No	No
Rogerio Souza	No	No	Advisory Board Actelion, Bayer, Pfizer, GSK	Actelion	No	Bayer, Pfizer	No
Sandra Fatima Menosi Gualandro	No	No	No	No	No	No	No
Tarso Augusto Duenhas Accorsi	No	No	No	No	No	No	No
Wilson Mathias Junior	No	No	No	No	No	No	No

**Table:** **Class of Recommendation:** reflecting the extent of the treatment effect

Class I	Benefit >>> Risk; Evidence and/or general agreement that a iven treatment or procedure is beneficial, usefull and efective
Class IIa	Benefit >> Risk; Conflicting evidence and/or a diversion of opinion about the benefit of the procedure, but the evidence supports that the treatment/procedure can help the patient;
Class IIb	Benefit ≥ Risk; Conflicting evidence and/or a diversion of opinion about the benefit of the procedure and, it is not well defined whether the treatment/procedure can help the patient;
Class III	Risk ≥ Benefit; Evidence or general agreement that the given treatment or procedure is not useful/effective, and in some cases may be harmful.

**Table:** Levels of Evidence

**A**	Evidence in several populations from randomized clinical trials and meta-analyses
**B**	Evidence in a limited group of populations from single randomized clinical trial or non-randomized clinical trials
**C**	Evidence in very limited group of populations from consensus and expert's opinions, case reports, and series

**Table:** Recommendations for Collection of Medical History

Recommendation	Class of recommendation	Level of evidence
Obtain clinical history directly from the patient undergoing the procedure	I	C
Collect information in a succinct, objective, and focused manner, considering only the data relevant to the risk stratification algorithm used	IIa	B
In situations where direct data collection from the patient is impossible, obtain the data from relatives, acquaintances, or with the health professional who is accompanying the patient	IIa	C
Do not collect information from the patient's clinical history in the perioperative risk assessment	III	C

**Table:** Recommendations for Performing a Physical Exam in a Patient Being Evaluated for Perioperative Risk of a Non-cardiac Surgery

Recommendation	Class of recommendation	Level of evidence
Perform a general and cardiovascular physical exam	I	C
Not perform physical exam in the perioperative risk assessment	III	C

**Table:** Recommendations for Requesting an Electrocardiogram^21-23,38,39^

Recommendation	Class of recommendation	Level of evidence
History and/or abnormalities in the physical exam suggestive of cardiovascular disease	I	C
Patients undergoing intracavitary surgeries, solid organ transplants, major orthopedic surgeries, and arterial vascular surgeries	I	C
High risk of events estimated by preoperative risk algorithms	I	B
Presence of DM	I	C
Obese patients	IIa	C
Patients with age above 40 years	IIa	C

**Table:** Recommendations for Requesting a Chest X-ray^21-23,38,39^

Recommendation	Class of recommendation	Level of evidence
Patients with history or diagnostic tests suggestive of cardiorespiratory diseases	I	C
Patients with age above 40 years	IIa	C
Intermediate and high-risk surgeries, mainly intrathoracic and intra-abdominal surgeries	IIa	C

**Table:** III. Recommendations for Requesting Laboratory Tests^21-23,38,39^ III.A. Complete Blood Count

Recommendation	Class of recommendation	Level of evidence
Clinical suspicion of anemia or presence of chronic diseases associated with anemia	I	C
History of hematological or hepatic diseases	I	C
Intermediate and high-risk surgeries, with prediction of bleeding and need for transfusion	I	C
All patients more than 40 years of age	IIa	C

**Table:** III.B. Hemostasis/Coagulation Tests

Recommendation	Class of recommendation	Level of evidence
Patients taking anticoagulant drugs, such as warfarin	I	C
Patients with hepatic impairment	I	C
Patients with clotting disorders (history of bleeding)	I	C
Intermediate and high-risk surgeries	I	C

**Table:** III.C. Dosage of Serum Creatinine

Recommendation	Class of recommendation	Level of evidence
Patients with nephropathy, DM, systemic arterial hypertension, hepatic failure, or heart failure (HF), and no serum creatinine test in the last 12 months	I	C
Intermediate and high-risk surgeries	I	C
All patients with age above 40 years	IIa	C

**Table:** Recommendations for Preoperative Echocardiography

Recommendation	Class of recommendation	Level of evidence
Patients with HF or suggestive symptoms undergoing intermediate or high-risk surgery, without evaluation in the last year, or who present clinical worsening	I	A
Patients with suspected moderate/important anatomical valve alteration undergoing intermediate or high-risk surgery, without evaluation in the last year, or who present clinical worsening	I	C
Patients who will undergo liver transplantation	I	B
Symptomatic patients with intracardiac prosthesis undergoing intermediate or high-risk surgery, without evaluation in the last year	IIa	C
Asymptomatic patients undergoing high-risk surgery	IIb	C
Routine in asymptomatic individuals without clinical suspicion of HF or moderate to severe valve disease undergoing intermediate or low-risk surgery	III	C

**Table:** Recommendations for the Preoperative Exercise Electrocardiogram Testing

Recommendation	Class of recommendation	Level of evidence
Patients with intermediate or high risk for complications (without severe perioperative cardiovascular conditions) and scheduled for arterial vascular surgery	IIa	C
Patients undergoing low-risk surgeries	III	C
Patients with low risk for complications undergoing low- or intermediate-risk surgery	III	C

**Table:** IV. Recommendations for Non-Invasive Tests to Detect Myocardial Ischemia

Recommendation	Class of recommendation	Level of evidence
Patients with intermediate or high risk for complications (without severe perioperative cardiovascular conditions) and scheduled for arterial vascular surgery	IIa	B
Patients with intermediate or high risk for complications AND scheduled for intermediate-risk operations AND low functional capacity	IIb	C
Patients undergoing low-risk surgeries	III	C
Patients with low risk for complications undergoing low-risk or intermediate-risk surgery	III	C

**Table:** Recommendations for Preoperative Coronary Angiography

Recommendation	Class of recommendation	Level of evidence
Patients with high-risk acute coronary syndromes	I	A
Patients with extensive ischemia in non-invasive tests to detect myocardial ischemia	I	B
Stable patients undergoing low-risk surgery	III	C

**Table:** Recommendations for hs-TnT Measurement in the Preoperative Period

Recommendation	Class of recommendation	Level of evidence
hs-TnT can be measured once before surgery in patients undergoing arterial vascular surgeries	IIa	B
hs-TnT can be measured once before surgery in patients with intermediate or high risk for complications, determined by perioperative assessment algorithms, who will undergo nonvascular surgeries	IIa	C

**Table:** Recommendations for Preoperative BNP/NTproBNP Measurement to Predict Risk of Perioperative Cardiovascular Events

Recommendation	Class of recommendation	Level of evidence
Patients undergoing arterial vascular surgeries	IIa	A
Patients more than 55 years old with at least one cardiovascular risk factor* undergoing nonvascular surgeries	IIa	C

* Diabetes, systemic arterial hypertension, dyslipidemia, smoking, and family history of early CAD.

**Table:** Recommendations for Patients with Systemic Arterial Hypertension

Recommendation	Class of recommendation	Level of evidence
If blood pressure is uncontrolled and there is enough time until the surgical procedure, therapy should be optimized to reduce blood pressure levels	I	C
Antihypertensive drugs (including ACE inhibitors) should be maintained preoperatively, including on the day of surgery	I	C
If the patient has high blood pressure and time is not enough to effectively control it, fast-acting β-adrenergic blockers (esmolol) should be used to avoid blood pressure increases during intubation; oral clonidine may be used in patients in whom β-blockers are contraindicated	I	C
Hypokalemia, if present, should be corrected before surgery	I	C
Resumption of antihypertensive therapy in the postoperative period, preferably the one used before surgery, should be performed as soon as possible	I	C
Optimization of blood volume should be performed throughout the perioperative period	I	C

**Table:** Recommendations for Patients with Heart Failure

Recommendation	Class of recommendation	Level of evidence
Elective surgeries in patients with decompensated HF (NYHA functional class III/IV) should be postponed until clinical compensation of the patient	I	C
Elective surgeries in patients with recent onset HF whose treatment has not yet been optimized should be postponed for at least 3 months to allow the use of drugs in adequate doses	I	C
All chronic-use drugs should be maintained in the perioperative period and reintroduced as early as possible postoperatively. If oral administration of drug is not possible, administration by nasoenteral or venous catheter should be considered	I	C
The use of a β-blocker should be maintained in the perioperative period. However, administration of high doses in patients who had not previously use the drug or increasing the usual dose is not recommended, unless there is sufficient time to adjust the dose before surgery	I	C

**Table:** V. Recommendations for Patients with Valvular Heart Disease

Recommendation	Class of recommendation	Level of evidence
Perform an echocardiogram in patients known or suspected to have moderate/relevant anatomical valve alteration undergoing intermediate or high risk surgery, without evaluation in the last year or who present clinical worsening	I	C
Patients with valvular heart disease with indication for interventional valve treatment should, as a priority, be submitted to cardiac treatment and subsequently to the proposed noncardiac surgery	I	B
Patients with severe asymptomatic AoS scheduled for intermediate- and high-risk elective noncardiac surgeries should undergo interventional valve treatment before noncardiac surgery	I	B
Patients with asymptomatic major regurgitant valvular heart disease may undergo elective noncardiac surgery	I	C

**Table:** V. Recommendations for Patients with Arrhythmias

Recommendation	Class of recommendation	Level of evidence
In patients with AF with ventricular response > 120 bpm, elective noncardiac surgery should be postponed until the HR is controlled	I	C
Maintain antiarrhythmic drugs used by the patient	I	C
Preoperative correction of triggering factors, such as electrolytic imbalances and hypoxemia	I	C
Venous magnesium supplementation may be considered when the serum level is below 2.0 mg/dL	IIa	B
Venous amiodarone in the early postoperative period may be considered in patients with an increased risk of developing POAF in pulmonary resection and esophagectomy surgeries	IIb	B

**Table:** Recommendations for Temporary Perioperative Pacemaker

Recommendation	Class of recommendation	Level of evidence
When general anesthesia is scheduled in urgent or emergency procedures in patients with indication for definitive pacemaker	I	C

**Table:** XI.A. Preoperative Class of Recommendation I

Recommendation	Class of recommendation	Level of evidence
Determine if the patient has a unicameral or bicameral pacemaker, resynchronizer, defibrillator, or multiple prostheses based on clinical history, physical examination, scar evaluation, electrocardiographic record, chest or abdomen X-ray, and previous evaluations in specialized clinics	I	C
Assess whether there is a risk of electromagnetic interference during the planned diagnostic and/or surgical procedure	I	B
Evaluate the presence of equipment in the surgical room with potential to generate electromagnetic fields that could interfere with the CIED	I	C
Patients with ICDs should be monitored with continuous ECG until the antitachycardia function is switched off	I	C
Determine the function of the device with an assessment by a specialist to adjust the program. In the absence of a specialist, at least assess whether there is an effective pacemaker spike (which generates command) in the ECG. Consult the manufacturer of the prosthesis for additional recommendations	IIa	C

**Table:** XI.B. Intraoperative

Recommendation	Class of recommendation	Level of evidence
Equipment for temporary artificial cardiac pacing and defibrillation must be present in the room and in conditions of immediate use	I	C
All patients should be monitored using continuous ECG and plethysmography (or auscultation, pulse palpation or ultrasound), regardless of the type of anesthesia	I	C
Electric scalpel, cardioversion, emergency defibrillation or radiotherapy: follow the recommendations described in item 4.G	I	C
Lithotripsy or magnetic resonance: follow the guidelines described in item 4.G	IIa	C

**Table:** XI.C. Postoperative^182,183^

Recommendation	Class of recommendation	Level of evidence
Heart rate and rhythm must be continuously monitored during the immediate postoperative period	I	C
Cardioversion/defibrillation equipment and cardiac stimulation support should be available	I	C
If the functions of the device have changed during the surgical procedure, normal condition should be restored as soon as possible through reprogramming	I	C
Antiarrhythmic drugs should be reintroduced as soon as possible	I	C

**Table:** I. F. Recommendations for Patients Undergoing Liver Transplant

Recommendation	Class of recommendation	Level of evidence
ECG and chest X-ray should be requested for all patients	I	C
Echocardiogram should be requested for all patients	I	B
For patients with an echocardiogram showing pulmonary artery pressure (PAP) > 45 mmHg, right heart catheterization should be requested with pulmonary artery pressure measurement	I	C
For patients with three or more risk factors for CAD*, a stress test with echocardiography or myocardial scintigraphy should be requested	IIa	B
Invasive coronary angiography should be performed in high-risk patients with positive stress tests, although hemorrhagic complications are more common and related conditions, such as elevated creatinine, may contribute to increased morbidity of cirrhotic patients	IIa	C
PCI with stent placement should consider that the patient may die due to liver disease while waiting for the minimum antiplatelet agent period and the real benefit of the intervention in minimizing perioperative risks	IIa	C
Phosphodiesterase inhibitors can be used to reduce PH in patients with PAP between 35 and 45 mmHg, although there is no conclusive evidence of the benefit of this approach	IIb	B
Perform hepatic transplantation in patients with severe PH in medical centers that do not offer aggressive therapies for PAP reduction or the possibility of concomitant lung transplantation	III	B

* Age > 50 years, hypertension, DM, smoking, and family history for early CAD.

**Table:** Recommendations for Patients Undergoing Renal Transplant

Recommendation	Class of recommendation	Level of evidence
Patients without major risk factors* are considered of low cardiovascular risk, without indication of additional testing	I	C
Patients with only one of the major risk factors* are considered as intermediate cardiovascular risk and should be submitted to noninvasive tests to detect myocardial ischemia. If positive, continue invasive investigation with invasive coronary angiography; if negative, perform renal transplantation	IIa	C
Patients presenting at least two of the major risk factors* are considered as high cardiovascular risk and should be referred directly to invasive coronary angiography before transplantation	IIa	C
Patients with obstructive CAD, involving the proximal segments of the major epicardial coronary arteries, may undergo PCI or CABG aimed at reducing cardiovascular risk	IIa	C

* Age > 50 years, DM, and previous evidence of cardiovascular disease

**Table:** Recommendations for Patients Undergoing Bariatric Surgery

Recommendation	Class of recommendation	Level of evidence
Exclude general contraindications for bariatric surgery: type 1 diabetes, drug or alcohol abuse, uncontrolled psychiatric illness, lack of understanding of the risks, alternatives and complications of the intervention, and lack of commitment to the need for nutritional supplementation and clinical follow-up	I	C
Perform evaluation of morbidity and mortality risk using the specific calculation tool for bariatric surgery:http://www.surgicalriskcalculator.com/bariatric-surgery-risk-calculator	I	B
Routinely use thromboprophylaxis with low-molecular-weight heparin (LMWH), prophylactic unfractionated heparin (UFH) 8/8h, fondaparinux, or the combination of pharmacological method and intermittent pneumatic compression (IPC)	I	C
For patients with BMI lower than or equal to 50 kg/m^2^, use higher doses of LMWH (enoxaparin 40 mg SC 12/12h) or UFH (7500 UI SC 8/8h) than those commonly used in prophylaxis of non-obese patients	IIa	B
For patients with BMI higher than 50 kg/m^2^, use higher doses of LMWH (enoxaparin 60 mg SC 12/12h)	IIa	B

**Table:** I. Indications of Carotid Endarterectomy or Angioplasty According to Symptoms and Degree of Stenosis

Recommendation	Class of recommendation	Level of evidence
Surgery or angioplasty (with stent) in symptomatic patients with stenosis > 70% when the rate of complications of the team/hospital is lower than 6%	I	A
Surgery or angioplasty (with stent) in asymptomatic patients with carotid stenosis > 70% and without high risk of surgical complications because the results are similar with the two techniques. It is important to note that the therapeutic option should be widely discussed with the vascular surgeon. In addition, for patients at high risk of complications, clinical treatment should be considered	IIa	B
Patients submitted to carotid angioplasty should be monitored with continuous ECG for at least 24 hours after the procedure because of the risk of bradycardia and hypotension	IIa	C
Surgery or angioplasty (with stent) in symptomatic patients with stenosis between 50 and 69% when the rate of complications of the team/hospital is lower than 6%	IIb	C
Carotid surgery or angioplasty in patients with stenosis < 50%	III	A

**Table:** II. Indications of Conventional or Endovascular Surgery of Aortic Aneurysm According to Surgical Risk

Recommendation	Class of recommendation	Level of evidence
In patients with high surgical risk and favorable anatomy, endovascular correction is preferable to open intervention because of lower perioperative mortality	IIa	B

**Table:** Recommendation in Patients Undergoing Dental Procedures

Recommendation	Class of recommendation	Level of evidence
In patients with heart disease, the use of small amounts of local anesthetics with vasoconstrictors (two to three 2.0% lidocaine tubes with 1:100,000 epinephrine) for dental procedures is safe and should be preferentially used	I	B

**Table:** Recommendations for Patients Taking Warfarin for Anticoagulation

Recommendation	Class of recommendation	Level of evidence
Patients taking warfarin should have an INR control at least 24 hours prior to the dental procedure	I	A
If INR < 3.0, suspension of the use of oral anticoagulant for simple surgical procedures (extraction of ≤ 3 teeth, gingival surgery, periodontal scraping) is not necessary. When INR ≥ 3.0 and the planned procedures are more extensive and/or postoperative bleeding occurs, the attending physician and dentist consider together the possible suspension of the drug in a timely manner for total or partial reversion of the anticoagulant effect	I	A

**Table:** Recommendations for Patients Taking Antiplatelet Agents

Recommendation	Class of recommendation	Level of evidence
Patients on secondary cardiovascular prevention on aspirin or clopidogrel monotherapy should not discontinue their use for dental procedures	I	B
Patients using DAPT for a recent PCI (6 weeks after BMS stent and 6 months after DES) or acute coronary syndrome in the last year should maintain their use in the perioperative period of dental procedures	I	B

**Table:** Preoperative Care

Recommendation	Class of recommendation	Level of evidence
Assess the patient's complete medical history	I	C
In patients taking warfarin, obtain the INR 24 hours before the dental procedure	I	A

**Table:** During the Procedures

Recommendation	Class of recommendation	Level of evidence
Minimize surgical trauma	I	C
Schedule a larger number of appointments when more than three teeth are extracted	I	C
Reduce areas of periodontal surgeries and scaling and root straightening (by sextant)	I	C
Plan surgeries for this type of patient at the beginning of the day and at the beginning of the week	I	C

**Table:** Bleeding Control in the Postoperative Period

Recommendation	Class of recommendation	Level of evidence
Removal of non-resorbable suture after 4-7 days	I	C
Compression with gauze for 15-30 minutes after the surgical procedure	I	C
Use of coagulant agents: gelatinous sponge, oxidized regenerated cellulose, synthetic collagen, tranexamic acid mouthwashes in 4.8% aqueous solution during and after 7 days of surgery, using 10 mL, 4 times a day for 2 minutes or ε-amino caproic acid mouthwash (when possible). In the first 24 hours, only mouthwash should be performed without chewing movements	I	C
Sutures suitable for wound closure	I	C

**Table:** Recommendations for Patients Undergoing Dermatologic Procedures

Recommendation	Class of recommendation	Level of evidence
ASA should be maintained in patients in secondary prevention of cardiovascular events undergoing any dermatological surgical intervention	I	B
Clopidogrel (monotherapy) may be maintained in patients in secondary prevention of cardiovascular events undergoing dermatological interventions	IIa	C
For patients who use DAPT for stent implantation and are not in the period of greatest thrombotic risk, maintain ASA and suspend the second antiplatelet drug	IIa	C
For patients who use warfarin and who will be submitted to dermatological procedures, maintain the medication with adjustment of INR values ≤ 3.5	IIa	C
For patients who use NOACs undergoing dermatological procedures, maintain the medication, ensuring that the surgical intervention is scheduled a few hours before the next dose	IIa	C

**Table:** Recommendations for Patients Undergoing Endoscopic Procedures

Recommendation	Class of recommendation	Level of evidence
For endoscopic procedures classified as low risk of bleeding, antiplatelet therapy (monotherapy or DAPT) or anticoagulant with warfarin should be maintained	I	B
Patients taking aspirin monotherapy for secondary prevention of cardiovascular events should maintain their use in the perioperative period of endoscopic procedures, including in most procedures considered to have high risk of bleeding	I	B
For endoscopic procedures classified as high risk of bleeding, anticoagulant therapy with warfarin or NOACs should be discontinued	I	B
Patients with DAPT after PCI should ideally not undergo high-risk bleeding endoscopic procedures within the ideal duration of the DAPT	I	B
Patients with high risk of bleeding who need to undergo endoscopic procedures before the end of ideal DAPT period after PCI, should maintain aspirin and suspend the second antiplatelet	IIa	C
For endoscopic procedures classified as low risk of bleeding, anticoagulant therapy with NOACs may be maintained	IIa	C

**Table:** Recommendations for Patients Undergoing Ophthalmologic Procedures

Recommendation	Class of recommendation	Level of evidence
For patients recommended to maintain anticoagulant and/or antiplatelet agents, the ophthalmologist should be informed of the need to ensure adequate hemostasis	I	B
Patients undergoing ophthalmologic surgeries and are using ASA for secondary cardiovascular prevention should maintain their use in the perioperative period	I	B
Patients undergoing ophthalmic operations for glaucoma or vitrectomy and are taking clopidogrel monotherapy should discontinue their use in the perioperative period	I	C
Patients undergoing vitrectomy or trabulectomy and are on warfarin anticoagulant therapy should discontinue their use in the perioperative period	I	B
Patients on clopidogrel monotherapy for secondary cardiovascular prevention who will undergo cataract surgeries should maintain their use in the perioperative period	IIa	B
Patients receiving DAPT for a recent PCI (6 weeks after BMS stent and 6 months after DES) or acute coronary syndrome in the past year and requiring interventions with a lower hemorrhagic risk (intravitreal injections, cataract and peribulbar anesthesia) should maintain the perioperative use of DAPT	IIa	B
Patients receiving DAPT for a recent PCI (6 weeks after BMS stent and 6 months after DES) or acute coronary syndrome in the past year and requiring interventions with a higher hemorrhagic risk (vitrectomy, trabulectomy) should maintain the use of ASA and discontinue P2Y12 receptor inhibitors in the perioperative period	IIa	C

**Table:** Recommendations for Using Perioperative β-blockers

Recommendation	Class of recommendation	Level of evidence
Patients already receiving β-blockers chronically must keep using them throughout the perioperative period	I	B
Patients with symptomatic ischemia (angina) or ischemia evidenced by functional test	IIa	B
For patients who started on β-blockers, titrate the drug progressively until an HR of 55 to 65 bpm is obtained and avoid hypotension (SBP < 100 mmHg)	IIa	B
Start β-blockers less than one week before surgery	III	B

**Table:** Recommendations for Using Statins in the Perioperative Period

Recommendation	Class of recommendation	Level of evidence
Patients scheduled for vascular surgeries	I	A
Patients submitted to nonvascular surgeries with clinical indication for the use of statins due to associated diseases (CAD, cerebrovascular disease, peripheral arterial disease, diabetes)	I	C
Maintain in patients who already use them	I	B

**Table:** V. C. Recommendations for Antiplatelet Agents

Recommendation	Class of recommendation	Level of evidence
For patients taking ASA for primary prevention, the recommendation is to suspend the antiplatelet agent 7 days before noncardiac surgery	I	A
For patients taking ASA for secondary prevention, the recommendation is to maintain it at a maximum dose of 100 mg daily	I	B
Suspend ASA 7 days before neurosurgery or transurethral resection of the prostate by the conventional technique (without using green light laser)	I	A
Patients with DAPT following PCI should not undergo elective surgeries during the ideal duration of DAPT: 6 weeks after BMS (Level of evidence B); 6 months after DES (Level of evidence A) or one year after PCI in the context of acute coronary syndromes	I	A or B, depending on time
Prasugrel (in patients with DAPT) should be discontinued 7 days before noncardiac surgeries with moderate or high bleeding risk	I	B
Clopidogrel and ticagrelor (in patients with DAPT) should be discontinued 5 days before noncardiac surgeries with moderate or high bleeding risk	I	B
Patients who need surgery before the expected end of DAPT after PCI should receive ASA 100 mg/day throughout the perioperative period. The clopidogrel should be suspended 5 days before the procedure and restarted immediately, ideally up to 5 days postoperatively	IIa	C
Maintenance of DAPT can be considered for patients who need surgery before the expected end of DAPT after PCI, for procedures performed in compressible sites or by endovascular technique and with an estimate of low risk of bleeding, depending on a multidisciplinary consensus	IIb	C
Patients at very high risk for stent thrombosis, such as diabetics, PCI involving graft, PCI in the context of acute coronary syndromes, or complicated PCI, may be considered for “bridge” therapy with parenteral antiplatelet consisting of glycoprotein IIb/IIIa inhibitors	IIb	B
Initiate ASA before noncardiac surgeries	III	C
LMWH "bridge" therapy	III	B

**Table:** Recommendations for Myocardial Revascularization (CABG or PCI) Before Noncardiac Surgeries

Recommendation	Class of recommendation	Level of evidence
Patients with indication for myocardial revascularization, regardless of the perioperative context, scheduled for elective noncardiac surgeries	I	C
Perform routine myocardial revascularization exclusively with the aim of reducing perioperative cardiac events	III	B
Perform myocardial revascularization in patients requiring emergency noncardiac surgery, regardless of severity of signs, symptoms, and degree of coronary obstruction	III	C
Perform myocardial revascularization in patients with severe prognostic limitation due to extracardiac conditions, with noncardiac surgical procedures planned, such as gastrostomies, digestive bypasses, and tracheostomies	III	B

**Table:** Recommendations for the Safety Interval between Elective Myocardial Revascularization and Noncardiac Surgery

Recommendation	Class of recommendation	Level of evidence
After CABG:		
Ideal time: more than 30 days	I	C
Minimum time: according to the postoperative recovery	I	C
After balloon PCI without stent:		
Ideal time: 14 days	I	B
After PCI with BMS:		
Ideal time: more than 6 weeks	I	B
Minimum time: 14 days	I	C
After PCI with DES:		
Ideal time: 6 months	I	A
Minimum time: 3 months	I	B

**Table:** Recommendations for the Safety Interval between Myocardial Revascularization (Cabg or Pci) in the Context of Acute Coronary Syndromes and Noncardiac Surgery

Recommendation	Class of recommendation	Level of evidence
Ideal time: 1 year, regardless of the revascularization strategy	I	B
Minimum time: equal to that proposed for each specific strategy in the elective context	I	C

**Table:** I. A. General, Abdominal and Pelvic, Urological, Gynecological, Vascular, and Plastic and Reconstructive Surgeries

Very low risk for VTE (< 0.5%, Caprini score 0): no indication for pharmacological (Class of recommendation I, Level of evidence B) or mechanical (Class of recommendation IIa, Level of evidence C) thromboprophylaxis in addition to recommendation of early ambulation
Low risk for VTE (~ 1.5%, Caprini score 1-2): preferably mechanical prophylaxis with IPC** (Class of recommendation IIa, Level of evidence C)**
Moderate risk for VTE (~ 3%, Caprini score 3-4) without high risk of bleeding complications: prophylactic doses of UFH or LMWH (**Class of recommendation IIa, Level of evidence B**) or mechanical prophylaxis (preferably IPC) (**Class of recommendation IIa, Level of evidence C**)
Moderate risk for VTE (~3%, Caprini score 3-4) with high risk of bleeding complications or in patients where the consequences of bleeding may be severe: mechanical prophylaxis, preferably with IPC (**Class of recommendation IIa, Level of evidence C**)
High risk for VTE (~ 6%, Caprini score ≥ 5) without high risk of bleeding complications: prophylactic doses of UFH or LMWH (**Class of recommendation I, Level of evidence B**). Addition of mechanical to pharmacological prophylaxis with the use of elastic stockings or IPC is suggested (**Class of recommendation IIa, Level of evidence C**)
High risk for VTE submitted to surgery for neoplasia without high risk of bleeding complications: extended prophylaxis with LMWH for 4 weeks (**Class of recommendation I, Level of evidence B**)
High risk for VTE with high risk of bleeding complications or in patients where the consequences of bleeding can be severe: mechanical prophylaxis with IPC until the risk of bleeding is reduced and pharmacological prophylaxis can be initiated (**Class of recommendation IIa, Level of evidence C**).

**Table:** I. B. Bariatric Surgeries

Recommendation	Class of recommendation	Level of evidence
Routinely use thromboprophylaxis with LMWH, prophylactic UFH 8/8h, fondaparinux, or association of a pharmacological method with IPC	I	C
For patients with BMI lower than or equal to 50 kg/m^2^, use higher doses of LMWH (enoxaparin 40 mg SC 12/12h) or UFH (7,500 IU SC 8/8h) than those usually used in prophylaxis of non-obese patients	IIa	B
For patients with BMI higher than 50 kg/m^2^, use higher doses of LMWH (enoxaparin 60 mg SC 12/12h)	IIa	B

**Table:** I. C. Thoracic Surgeries

Moderate risk surgery for VTE (most thoracic surgeries) without high risk of bleeding complications: prophylactic doses of UFH or LMWH (Class of recommendation IIa, Level of evidence B) or mechanical prophylaxis with IPC (Class of recommendation IIa, Level of evidence C)
High risk surgery for VTE (extensive pulmonary resection, pneumectomy, extrapleural pneumonectomy, and esophagectomy) without high risk of bleeding complications: prophylactic doses of UFH or LMWH (**Class of recommendation I, Level of evidence B**). Addition of mechanical prophylaxis with elastic stocking or IPC is suggested (**Class of recommendation IIa, Level of evidence C**)
Moderate or high risk surgery for VTE with high risk of hemorrhagic complications: mechanical prophylaxis with IPC (**Class of recommendation IIa, Level of evidence C**)

**Table:** I. D. Craniotomies

Recommendation	Class of recommendation	Level of evidence
Most craniotomies (considered high risk for VTE): mechanical prophylaxis with IPC	IIa	C
Surgeries considered high risk for VTE (associated with neoplastic diseases): add prophylactic doses of UFH or LMWH to mechanical prophylaxis with IPC as soon as there is adequate hemostasis and decreased risk of bleeding	IIa	C

**Table:** I. E. Spinal Surgeries

Recommendation	Class of recommendation	Level of evidence
Most spinal surgeries: mechanical prophylaxis with IPC	IIa	C
Surgeries with high risk of VTE (associated with neoplasias or anteroposterior access): add pharmacological prophylaxis (UFH or LMWH) to mechanical prophylaxis (IPC) as soon as there is adequate hemostasis and decreased risk of bleeding	IIa	C

**Table:** I. F. Surgery for Major Trauma

Recommendation	Class of recommendation	Level of evidence
Most major trauma: prophylactic doses of UFH or LMWH, or mechanical prophylaxis with IPC (if there is no contraindication for injury to the lower limbs)	IIa	C
Major trauma with high risk of VTE (acute spinal cord injury, traumatic brain injury, traumatic spinal surgery): association of pharmacological and mechanical prophylaxis with IPC (if there is no contraindication for lower limb injury)	IIa	C
Trauma with high risk of bleeding with contraindication to the use of UFH or LMWH: mechanical prophylaxis with IPC (if there is no contraindication for lower limb injury) until there is a decreased risk of bleeding and the possibility of introducing pharmacological prophylaxis	IIa	C
Trauma in general: do not use inferior vena cava filter as primary prevention for VTE in major trauma	III	C

**Table:** II. A. Knee or Hip Prosthesis Surgery

Use prophylaxis for at least 10 to 14 days with LMWH, fondaparinux, apixaban, dabigatran, rivaroxaban, UFH (**Class of recommendation I, Level of evidence B**), or mechanical prophylaxis with IPC (**Class of recommendation I, Level of evidence C**). Extend outpatient prophylaxis for up to 35 days from the day of surgery (**Class of recommendation IIa, Level of evidence B**)

**Table:** II. B. Hip Fracture Surgery

Use prophylaxis for at least 10 to 14 days with LMWH, fondaparinux, UFH (**Class of recommendation I, Level of evidence B**), or mechanical prophylaxis with IPC (**Class of recommendation I, Level of evidence C**). Extend outpatient prophylaxis for up to 35 days from the day of surgery (**Class of recommendation IIa, Level of evidence B**)

**Table:** II. C. Major Orthopedic Surgeries Associated with High Risk of Hemorrhagic Complications

Use mechanical prophylaxis with IPC until there is a reduction in the risk of bleeding and the possibility of associating pharmacological prophylaxis (**Class of recommendation IIa, Level of evidence C**)

**Table:** II. D. Patients with Lesions in Lower Limbs, Distal to the Knee, Requiring Immobilization

No recommendation for thromboprophylaxis (**Class of recommendation IIa, Level of evidence C**)

**Table:** II. E. Knee Arthroscopy without Previous History of Venous Thromboembolism

No recommendation for thromboprophylaxis (**Class of recommendation IIa, Level of evidence B**)

**Table:** Recommendations I.A. Patients at High Risk for Thromboembolism

Recommendation	Class of recommendation	Level of evidence
Suspend warfarin 5 days before surgery and wait for INR < 1.5	I	C
Perform bridge therapy with UFH or LMWH at full dose when INR < 2	IIa	C
Suspend UFH 4-6 hours and LMWH 24 hours before the procedure	IIa	C
In the postoperative period, restart UFH or LMWH at full dose and warfarin at least 24 hours after the surgical procedure and suspend heparin only when INR is within the therapeutic range	IIa	C
In patients submitted to surgeries with high risk of bleeding, restart LMWH 48 to 72 hours after surgery	IIa	C

**Table:** I. C. Patients with Low Risk of Thromboembolism

Recommendation	Class of recommendation	Level of evidence
Do not use bridge therapy (suspend warfarin 5 days before surgery and wait for INR < 1.5 for the procedure)	IIa	C
Prophylactic UFH or LMWH, if indicated, may be used in the preoperative period	IIa	C
In the postoperative period, use prophylactic UFH or LMWH if indicated and restart warfarin 12 to 24 hours after the procedure	IIa	C

**Table:** Recommendations for patients with warfarin undergoing urgent or emergent surgeries

Recommendation	Class of recommendation	Level of evidence
Suspension of the antiplatelet agent, intravenous administration of vitamin K, and replacement of the deficient factors with prothrombin concentrate or FFP according to the availability of these products	I	C

**Table:** 

Recommendation for patients on chronic use of dabigatran	Class of recommendation	Level of evidence
Patients on chronic use of dabigatran with normal renal function may have the drug suspended 24 hours before surgery	I	C
In cases of moderate renal dysfunction (creatinine clearance 30-50 mL/min) or surgeries with high risk of bleeding, such as neurosurgeries, dabigatran should be suspended at least 48 hours before surgery	I	C
In cases of regional anesthesia with an epidural catheter, wait at least 6 hours after catheter withdrawal to initiate the first dose of dabigatran	I	C
Reintroduce the full dose of dabigatran for at least 24 hours after the end of the surgery, provided there is adequate hemostasis	IIa	C
In patients at high risk of bleeding, consider reintroduction of dabigatran after 48-72 hours	IIa	C

**Table:** 

Recommendation for patients on chronic use of rivaroxaban	Class of recommendation	Level of evidence
Patients on chronic use of rivaroxaban with normal renal function may suspend administration of the drug 24 hours before surgery	I	C
In cases of severe renal dysfunction (creatinine clearance 15-30 mL/min) or in surgeries with high risk of bleeding, such as neurosurgeries, rivaroxaban should be suspended at least 48 hours before the intervention	I	C
In cases of regional anesthesia with epidural catheter, wait at least 6 hours after catheter withdrawal for the next dose of rivaroxaban. In cases of epidural catheter maintained postoperatively for analgesia, withdrawal should occur after 18 hours of the last dose	I	C
Reintroduce the full dose of rivaroxaban at least 24 hours after the end of surgery, provided there is adequate hemostasis	IIa	C
In patients at high risk of bleeding, consider reintroducing the drug after 48-72 hours	IIa	C

**Table:** 

Recommendation for patients on chronic use of apixaban	Class of recommendation	Level of evidence
Patients on chronic use of apixaban with normal renal function may have the drug suspended 24 hours before surgery	I	C
In cases of moderate renal dysfunction (creatinine clearance 15-50 mL/min) or surgeries with high risk of bleeding, such as neurosurgeries, apixaban should be suspended at least 48 hours before the intervention	I	C
In cases of regional anesthesia with an epidural catheter, wait at least 6 hours after catheter withdrawal for the next apixaban dose	I	C
Reintroduce the full dose of apixaban at least 24 hours after the end of surgery, provided there is adequate hemostasis	IIa	C
In patients at high risk of bleeding, consider reintroducing the drug after 48-72 hours	IIa	C

**Table:** Recommendations for Infectious Endocarditis Prophylaxis before Dental Procedures

Recommendation	Class of recommendation	Level of evidence
Patients with risk conditions for severe IE (Chart 12)	I	B
Patients with other risk conditions for IE (Chart 12)	IIa	C

**Table:** Recommendations for Prophylaxis of Infectious Endocarditis before Genitourinary and Gastrointestinal Tract Procedures

Recommendation	Class of recommendation	Level of evidence
Patients with risk conditions for severe IE (Chart 12)	I	C
Patients with other risk conditions for IE (Chart 12)	IIa	C

**Table:** 

Recommendation	Class of recommendation	Level of evidence
Patients with intermediate to high perioperative cardiac risk assessment of ischemia should be monitored in semi-intensive or ICUs with troponin and ECG daily until the third postoperative day	I	B

**Table:** 

Recommendation for patients with perioperative MI	Class of recommendation	Level of evidence
Perioperative MI should be made using the criteria of the Universal Definition of MI^445^	I	C
Patients with perioperative MI with ST-alterations should be submitted to primary PCI as soon as possible	I	B
Patients with perioperative MI without ST-alterations should undergo optimization of secondary causes (anemia, hemodynamic instability, arrhythmias, and hypertension)	IIa	C
Patients more than 75 years old with perioperative MI, anterior wall ischemia on ECG, cardiogenic shock, electrical instability, or recurrent angina should be submitted to early invasive stratification	IIa	C
Treat the isolated increase of troponin levels as MI with double antiplatelet therapy and anticoagulation	III	C

**Table:** 

Recommendation for patients with POAF	Class of recommendation	Level of evidence
After POAF diagnosis, volume and electrolyte optimization is recommended, as well as correction of possible causal factors, such as infection, bleeding, and myocardial ischemia, in addition to pain and nausea	I	C
Perform continuous cardiac monitoring	I	C
After correction of causal factors, heart rate pharmacological reversion or control should be considered, considering the AF guideline	I	C
After 48 hours of persistent POAF, risks and benefits of anticoagulation should be considered, considering the clinical scores (CHADS_2_/CHA_2_DS_2_-VASC and HAS-BLED), in addition to the surgical conditions	I	C
Synchronized electrical cardioversion can be performed only when POAF compromises hemodynamics	IIa	C

**Table:** 

Recommendation for patients with AHF	Class of recommendation	Level of evidence
In addition to clinical evaluation, echocardiography should be performed to diagnose structural heart disease	I	B
Measurement of NT-proBNP or BNP levels should only be performed in case of diagnostic uncertainty	I	B
In addition to the usual acute HF treatment, the cause of HF should be investigated, particularly acute coronary disease, and serial measurement of troponin levels is indicated	I	B

**Table:** Recommendations for the Diagnosis of Deep Venous Thrombosis

Recommendation	Class of recommendation	Level of evidence
In patients with low probability of VTE, venous Doppler is not necessary unless D-dimer is positive	I	A
Venous Doppler for patients with intermediate to high probability	I	A
Venography only in cases where venous Doppler is not available or with uncertain results	I	A
Venography by MRI or angiotomography may be an alternative for the diagnosis of DVT	I	A
Use D-dimer alone for diagnosis of DVT	III	A

**Table:** Recommendations for the Diagnosis of Pulmonary Thromboembolism

Recommendation	Class of recommendation	Level of evidence
Diagnosis of patients with clinical suspicion of PTE should be confirmed using an imaging test and pulmonary Angio-CT should be selected	I	A
Pulmonary V/Q scintigraphy can be performed in patients when pulmonary Angio-CT is contraindicated or inconclusive or negative, and there is clinical suspicion of PTE	I	A
Pulmonary digital angiography can be performed in patients when Angio-CT and V/Q scintigraphy are contraindicated, or the results were inconclusive	I	A

**Table:** Recommendations for the Anticoagulant Agent for Venous Thromboembolism Treatment

Recommendation	Class ofrecommendation	Level of evidence
In patients with DVT or PTE and without cancer, 3-month long-term treatment with dabigatran, rivaroxaban, apixaban, or edoxaban is preferred compared to the use of vitamin K antagonists (warfarin)	IIa	C
In the absence of the new anticoagulants (dabigatran, rivaroxaban, apixaban, or edoxaban) in patients with DVT or PTE and without cancer, the use of warfarin for long-term treatment is preferred compared to use of LMWH	IIa	C
In patients with DVT or PTE with cancer, long-term (first three months) treatment with anticoagulant therapy with LMWH is preferred compared to warfarin, dabigatran, rivaroxaban, apixaban, and edoxaban	IIa	C
In patients with DVT or PTE requiring extended anticoagulant therapy, there is no need to change the anticoagulant initially used after the first three months for no reason	IIa	C

**Table:** Recommendations for the Duration of Anticoagulant Therapy

Recommendation	Class of recommendation	Level of evidence
In patients with proximal DVT or PTE due to surgical procedures, anticoagulant treatment is recommended for 3 months	I	B
In patients with DVT or PTE associated with cancer who do not have a high risk of bleeding, indefinitely extended therapy is preferred compared to therapy for 3 months	I	B
In patients with VTE and cancer associated with a high risk of bleeding, indefinitely extended therapy is preferred compared to therapy for 3 months	IIa	B
In patients with isolated distal DVT of the lower limbs caused by surgery or transient non-surgical risk factor when anticoagulation was selected, anticoagulant therapy is recommended for three months	IIa	C

**Table:** Recommendations for Patients with Acute Distal Deep Venous Thrombosis of the Lower Limb

Recommendation	Class of recommendation	Level of evidence
For patients using anticoagulation, use the same anticoagulant that would be used in case of acute proximal DVT	I	C
For patients using serial follow-up with imaging exam, do not use anticoagulation if there is no thrombus extension	I	B
Use of anticoagulation is suggested if the thrombus extends to the proximal veins after serial image test	I	C
In the absence of major symptoms or risk factors for thrombus extension, serial imaging (Doppler of the lower limbs) of the deep veins within 2 weeks is preferred to anticoagulation. In patients with important clinical symptoms or risk factors for thrombus extension, anticoagulation is preferentially recommended compared to serial monitoring by deep vein imaging	IIa	C
Anticoagulation is suggested if after serial imaging test, the thrombus extends but remains confined to the distal veins	IIa	C

**Table:** 

Aditional Recommendations for Patients with Deep Venous Thrombosis	Class of recommendation	Level of evidence
In patients with acute proximal DVT of the lower limbs, the use of anticoagulant therapy alone is suggested rather than the use of catheter-directed thrombolysis	IIa	C
Use inferior vena cava filter in patients with acute proximal DVT or PTE treated with anticoagulants	III	B
Routinely use compression stockings in patients with acute lower limb DVT to prevent post-thrombotic syndrome	III	B

**Table:** Recommendations for Patients with Subsegmental Pulmonary Embolism

Recommendation	Class of recommendation	Level of evidence
For patients with subsegmental PTE (without involvement of the more proximal pulmonary arteries) who do not present evidence of DVT of the lower limbs and who have a low risk of recurrence of VTE, clinical follow-up is preferred compared to anticoagulant therapy	IIa	C
In patients with a high risk of recurrence of VTE, the use of anticoagulant therapy is preferred compared to clinical follow-up	IIa	C

**Table:** 

Recommendation for Home Treatment of Pulmonary Thromboembolism	Class of recommendation	Level of evidence
Patients with low-risk PTE with adequate home conditions, home treatment or early hospital discharge is suggested (even before the first 5 days of treatment)	IIa	B

**Table:** Recommendations for Performing Systemic Thrombolysis

Recommendation	Class of recommendation	Level of evidence
In patients with acute hypotension-associated PTE (SBP < 90 mmHg) without a high risk of bleeding	IIa	B
In selected patients with significant clinical deterioration following the initiation of anticoagulant therapy (tachycardia, SBP fall, jugular stasis, gas exchange worsening, shock signs, progressive RV dysfunction in echocardiogram, or increased cardiac markers, such as troponin and BNP), but have not yet developed hypotension (SBP < 90 mmHg) and have a low risk of bleeding	IIb	C
In most patients in the absence of hypotension	III	B

**Table:** II. J. Pulmonary Thromboembolism Therapy with Catheter Intervention^486^

Recommendation	Class of recommendation	Level of evidence
In patients with acute PTE candidates for thrombolytic therapy, peripheral vein administration is preferred compared to direct catheter-mediated administration	IIa	C
In selected patients with acute PTE with hypotension and high risk of bleeding, failed systemic thrombolysis, or developed shock signs that can lead to death before the effect of systemic thrombolysis (in a period of hours), catheter-assisted mechanical removal of the thrombus is suggested if resources and staff are available	IIa	C

**Table:** II. K. T Recurrent Pulmonary Thromboembolism During Anticoagulant Therapy^486^

Recommendation	Class of recommendation	Level of evidence
In patients with recurrent VTE using warfarin with adequate INR, dabigatran, rivaroxaban, apixaban, or edoxaban, it is suggested to change the treatment for LMWH at least temporarily	IIa	C
In patients with recurrent VTE correctly using LMWH, it is suggested that the dose of LMWH is increased by one-third to one-quarter	IIa	C

**Table:** General Recommendations for Patients with Diabetes Mellitus^502-507^

Recommendation	Class of recommendation	Level of evidence
Request fasting glycemia and glycated hemoglobin (HbA1c) for all DM patients	I	C
Maintain fasting blood glucose between 90 and 130 mg/dL, postprandial blood glucose (2h) up to 180 mg/dL, and HbA1c < 7.0%	I	A
Individualization of goals should be considered for elderly patients, patients with HF, and pregnant women	I	C
Suspend oral drugs for diabetes control and modify the insulin scheme as indicated in chart 15 and 16	I	C
Adjustment of drug doses aiming at better glycemic control may require assistance from a specialist, especially for patients using insulin therapy	I	C
Patients with HbA1c > 9.0% (average blood glucose of 212 mg/dL) should receive blood glucose control measures before elective surgeries. Request expert consultation (if available) for faster glycemic control optimization	I	C

**Table:** Recommendations for Glycemic Controlin Hospitalized Patients with Diabetes Mellitus

Recommendation	Class of recommendation	Level of evidence
Monitoring of capillary glycemia (Level of evidence A); in patients taking oral drugs: fasting and preprandial and in patients taking insulin: pre-prandial and before bedtime (Level of evidence C)	I	A e C
Control goals for patients with hyperglycemia (may be different in specific subgroups, such as pregnant women, elderly, and patients with severe comorbidities and HF): - Pre-prandial glycemia between 80 and 140 mg/dL - Random glycemia up to 180 mg/dL- Avoid hypoglycemia: < 70 mg/dL - Reassess insulin doses if glycemia < 100 mg/dL	I	C
For rapid in-hospital glycemic control, insulinization should be used in several schemes (basal-prandial insulin with glycemic correction)	I	C

**Table:** Recommendations for Glycemic Control on the Day of Surgery (Fasting)

Recommendation	Class of recommendation	Level of evidence
Operate patients with DM preferably on the first hour of the day, especially insulin users	I	C
Avoid hypoglycemia and glycemic variability	I	C
Monitor capillary glucose every 6 hours in patients using oral drugs and every 4 hours in insulin users	I	C
Maintain glycemia between 80 and 180 mg/dL	I	C

**Table:** Postoperative Recommendations^511,512^

Recommendation	Class of recommendation	Level of evidence
Avoid hypoglycemia	I	A
Venous insulin therapy only for patients admitted to ICUs with high values (> 180 or 200 mg/dL)	I	A
For patients submitted to elective surgery with no complications, and postoperative period outside ICUs, the hypoglycemic scheme used before surgery may be continued	IIa	C
Reintroduce oral antidiabetics, initially in lower doses, as soon as the oral diet is reestablished	IIa	C
Metformin should be postponed until the risk of renal hypoperfusion is minimal. It should be postponed or not restarted in patients with significant renal, cardiac, and hepatic failure	IIa	C
Thiazolidinediones should not be used if the patient develops edematous conditions, especially pulmonary congestion due to HF or hepatic changes	IIa	C

**Table:** 

Recommendations for patients with hypothyroidism	Class of recommendation	Level of evidence
TSH dosage^514-516^ in the perioperative period of patients with risk of hypothyroidism or more than 65 years, mainly women	IIa	C
Patients undergoing hypothyroidism treatment should have normal TSH within the last 3 to 6 months to be considered adequately treated	IIa	B
For newly diagnosed hypothyroidism, in patients < 45 years and without comorbidities, start T4 (levothyroxine) 1.6 mcg/kg/day early while fasting or at bedtime	IIa	B
For newly diagnosed hypothyroidism, in patients > 45 years and without comorbidities, start levothyroxine 50 mcg/day and increase 25 mcg every 2 to 4 weeks	IIa	B
For the elderly and coronary patients, the initial dose should be 12.5 to 25 mcg and increase 12.5 to 25 mcg every 2 to 4 weeks	IIa	B
Wait for the patient with subclinical hypothyroidism to become euthyroid	III	B

**Table:** II. B. General Recommendations for Patients with Hyperthyreoidism

Recommendation	Class of recommendation	Level of evidence
Parallel evaluation by an endocrinologist should be strongly considered in the perioperative period of patients with hyperthyroidism	IIa	B
Before the elective procedure, patients should be adequately treated with drugs for hyperthyroidism; patients should only be released for surgery 3 to 8 weeks after the control of hyperthyroidism	IIa	B
Antithyroid drugs: the most commonly used are propylthiouracil (PTU) and methimazole. They inhibit the synthesis of thyroid hormones by preventing oxidation and organization of iodine. PTU has the additional benefit of inhibiting the peripheral conversion of T4 to T3 at high doses. Therefore, it is more widely used in the perioperative period. The usual dose is 100 mg every 8 hours, and the maximum dose is 400 mg for the same period. Doses of methimazole range from 10 to 120 mg daily in a single dose. The dose should be reevaluated every 4 to 6 weeks. Adverse effects are rarely serious: skin rash, fever, pruritus and arthralgia, transient increases in liver enzymes, and leukopenia. Agranulocytosis (0.5%), severe hepatitis, lupus-like syndrome, and thrombocytopenia are more severe and less frequent complications and require suspension of drug. Patients treated with PTU in the perioperative period should receive an equivalent dose of methimazole at discharge. Since this drug is more potent, it is easier to take and increases adherence	IIa	B
β-blockers: propranolol is the most commonly used at a dose of 10-80 mg every 6-8 hours (1.0 mg intravenously in the intraoperative period). Esmolol can be administered intraoperatively with an attack dose of 500 mcg/kg in 1 minute and maintenance of 25-300 mcg/kg/min	IIa	B

**Table:** II. C. Recommendations for Urgent or Emergency Surgical Procedures for Patients with Hyperthyreoidism

Recommendation	Class of recommendation	Level of evidence
β-blockers: prefer intravenous use; 0.5-1 mg propranolol in 10 min and 1-2 mg every 10 min	I	B
Iodine: it can be used for a maximum of 10 days because the inhibition of the organism (Wolff-Chaikoff effect) is transient and after that hyperthyroidism is worsened	I	B
Patients with subclinical hyperthyroidism may undergo urgent or elective surgeries. Those with cardiovascular symptoms or more than 50 years old should use β-blockers in the perioperative period	I	B
Corticosteroid: it should be administered in the perioperative period when there is no compensation of hyperthyroidism in the preoperative period to inhibit the peripheral conversion of T4 to T3. The hydrocortisone dose is 100 mg at induction and 100 mg every 8 hours in the first 24 hours. Another potential indication of the corticosteroid in this situation is the concomitance, although very rare, with Addison's disease and autoimmune thyroiditis	IIa	B
Antithyroid drugs: the drug of choice is PTU in high doses (1,000 to 1,200 mg daily, divided in three doses)	IIb	B
Lugol's solution, which contains 5% iodine and 10% potassium iodide, is the most used at a dose of 0.1 to 0.3 mL every 8 hours (3 to 5 drops); one hour after the thionamides (to avoid exacerbation of the organism)	IIb	B
Iodinated contrasts: sodium potassium and iopanoic acid are used for compensation, with the advantage of less leakage and inhibiting peripheral conversion of T4 to T3. The dose is 500 mg every 8 hours	IIb	B
Anesthesia: special attention should be given to increased metabolism of anesthetic drugs and to the risk of difficult intubation due to the presence of goiter	IIb	B
Thyrotoxic storm: it is associated with mortality rates of 20-30%. Given this severe clinical outcome, the treatment described above should be started promptly, even without laboratory confirmation	IIb	C

**Table:** 

Recommendation	Class of recommendation	Level of evidence
In the suspected diagnosis of adrenal insufficiency, patients should receive empirical treatment and have subsequent diagnostic confirmation	I	C

**Table:** III. Recommendations for Patients with Adrenal Insufficiency

Recommendation	Class of recommendation	Level of evidence
Confirm the diagnosis using appropriate tests for patients at risk for AI and consider a collaborative follow-up with an endocrinologist	I	B
If cases of need for confirmation of AF with tests, use dexamethasone, which does not interfere with the confirmatory tests	I	C
In cases of coexistence of untreated hypothyroidism and AI: first correct the AI	I	C
No need for mineralocorticoid supplementation because the corticoid doses for supplementation in surgical stress have mineralocorticoid activity, except in cases of dexamethasone replacement	I	C
If unable to confirm the diagnosis before surgery, we recommend corticoid supplementation based on the diagrams in chart 17	IIa	C
All patients submitted to emergency surgery should receive corticosteroid replacement empirically on suspicion of AAI based on the extent of the surgery	IIb	B

**Table:** IV. Recommendations for Doses of Corticoid Supplementation^525-527^

Recommendation	Class of recommendation	Level of evidence
Use high doses of corticosteroid supplementation to prevent AAI (may increase the chance of complications, such as hypertension and diabetes decompensation)	III	B

**Table:** IV. A. Mild Surgical Stress

Recommendation	Class of recommendation	Level of evidence
Double or triple the dose of corticosteroid in patients with AI and chronic users, noting that adrenal suppression can occur quickly when using high doses or even after a long time without using corticosteroids (up to 24-48 months)	IIa	C
If the patient is fasting, supplement with 50 mg of hydrocortisone intramuscularly or intravenously immediately before the surgery and maintain 25 mg of hydrocortisone twice a day or equivalent, reducing to a regular dose within 24 hours or as soon as the stress has subsided	IIa	C
In patients without definitive diagnosis but with strong suspicion, treat as if diagnosed with AI	IIb	C

**Table:** IV. B. Moderate Surgical Stress

Recommendation	Class of recommendation	Level of evidence
Supplement with 25 mg of hydrocortisone or equivalent, intramuscularly or intravenously, every 8 hours, starting on the morning of the surgery, with a 50% reduction in the daily dose up to the regular dose	IIa	C

**Table:** IV. C. High Surgical Stress

Recommendation	Class of recommendation	Level of evidence
Supplement with 50 mg of hydrocortisone or equivalent, every 8 hours, with a 50% reduction in the dose per day until regular dose is achieved or once metabolic stress ceases (usually lasts up to 48 hours in surgeries without complications due to infections or other causes)	IIa	C

**Table:** II. Specific Recommendations for Preoperative Evaluation in Elective Surgeries of Obese Patients^531-533^

Recommendation	Class of recommendation	Level of evidence
Complete history and physical examination	I	B
Track respiratory sleep disorders using appropriate score and referral for evaluation with a specialist in sleep disorders, if screening is positive	IIa	B
Evaluate the airway due to the risk of difficulty or failure in intubation. Circumference of the neck greater than 60 cm is associated with a significant increase in risk	IIa	B
ECG for patients with coronary diseases, arrhythmias, peripheral arterial disease, and cerebrovascular or cardiac structural disease, except in case of low-risk surgery	IIa	B
Fasting glycemia	IIa	B
Creatinine if patient has diabetes, hypertension, or history of nephropathy	IIa	C
Additional tests, such as coagulation studies and functional lung tests, are not mandatory and should not be routinely used in the preoperative evaluation of obese individuals. Additional tests should be selected based on medical history	IIa	B
ECG can be considered for asymptomatic patients without coronary disease and to be submitted to surgery with intermediate or high risk	IIb	B
Echocardiogram for individuals with dyspnea of unknown origin or with diagnosis of HF and worsening dyspnea or clinical condition	IIb	B
Reassessment of ventricular function can be considered in stable patients with a last ECO more than one year ago	IIb	C
Noninvasive oximetry can be helpful. If saturation is lower than 95%, additional assessment is indicated due to the risk of significant respiratory disease	IIb	C

**Table:** III. Recommendations to Reduce the Risk of Obese Patients^531,533,535-537^

Recommendation	Class of recommendation	Level of evidence
Cessation of smoking six weeks before surgery	I	B
Respiratory physiotherapy	IIa	C
If sleep apnea documented by polysomnography, consider installing CPAP preoperatively in patients who do not use CPAP, and do not discontinue CPAP in those who already use it	IIa	B
Early ambulation	IIa	B
Recommend men to remove beards to avoid difficulties in placing the mask for ventilation, if needed	IIa	C

**Table:** III. A. Intraoperative Care of Obese Patients

Recommendation	Class of recommendation	Level of evidence
Monitoring blood pressure with appropriate cuff for obese	I	B
Provide appropriate equipment for obese, including stretchers, surgical tables, and chairs. Care with lesions due to positioning in the surgical bed	IIa	C
Reverse Trendelemburg positioning during anesthetic induction	IIa	B
Pre-oxygenation (performed by providing 100% oxygen through a mask with the patient breathing spontaneously for a period of three minutes) or sitting with head elevated	IIa	B
Application of positive end-expiratory pressure (PEEP) improves oxygenation and prevents atelectasis	IIa	B
Rapid sequence of anesthetic induction with cricoid pressure during intubation	IIa	B
Prefer regional anesthesia, whenever possible	IIa	B^538^
An anesthesia team with experience in managing obese patients and additional staff to adequately move the patient and for potential complications are recommended	IIa	C^539^

**Table:** III. B. Postoperative Care of Obese Patients

Recommendation	Class of recommendation	Level of evidence
Postoperative care in ICUs for patients at high risk due to comorbidities who had postoperative extubation failure and suffered intraoperative or superobese complications (BMI > 70)	I	C
Handle the patient in a sitting or bedside position, raise to 45 Classs, and elevate chin	I	C
Continuous non-invasive oximetry during anesthesia recovery, measurement after recovery from anesthesia (if normal, does not need to be repeated), and continuous measurement during sleep (in interventions with intermediate to high extent in patients with apnea)	I	C
Supplement with oxygen until patient has mobility	I	C
Install CPAP in cases of prior diagnosis of sleep apnea and residential use of the equipment	I	B
Maintenance of normovolemia	IIa	C
Respiratory physiotherapy for all patients submitted to intermediate- to high-risk surgery	IIa	C

**Table:** Prophylaxis for Deep Venous Thrombosis in Obese Patients

Recommendation	Class of recommendation	Level of evidence
Drug prophylaxis with LMWH or UFH	I	A

**Table:** Recommendations for Perioperative Transfusion of Red Blood Cell Concentrates

Recommendation	Class of recommendation	Level of evidence
Asymptomatic patients without baseline ischemic heart disease should receive hemoglobin ≤ 7.0 g/dL (restrictive transfusion trigger)	I	A
Patients with anemia and evidence of organic ischemia, with risk or presence of bleeding, and who are susceptible to complications resulting from inadequate oxygenation should be transfused	I	C
In cases of acute coronary syndrome, a more liberal transfusion strategy (maintaining Hb > 8.0 g/dL) is recommended	I	C

**Table:** Recommendations for Perioperative Management In Patients with Sickle Cell Disease (SS/SC/Sβtal)^556-561^

Recommendation	Class of recommendation	Level of evidence
Careful preoperative hydration, oxygenation monitoring, and meticulous postoperative management including respiratory physiotherapy are indicated for all patients submitted to general anesthesia	I	C
In patients submitted to minor surgical procedures not requiring general anesthesia, preoperative transfusion is not indicated routinely	I	C
For patients submitted to low/intermediate risk procedures (including laparoscopic cholecystectomy), preoperative transfusion is recommended to raise the hemoglobin levels to 10 g/dL	I	C*
Partial transfusion to reduce hemoglobin S levels to 30% or lower should be considered for high-risk procedures and for patients with a history of pulmonary disease requiring prolonged anesthesia	I	C†

* if patient has hemoglobin levels 9, clinical evaluation with hematologist required;

† clinical evaluation with hematologist required

**Table:** Recommendations for Platelet Transfusion^568^

Recommendation	Class of recommendation	Level of evidence
For major surgeries or invasive procedures, such as lumbar puncture, epidural anesthesia, liver biopsy, endoscopies with biopsy, and placement of a central venous catheter, when the platelet count is lower than 50,000/mm^3^	IIa	C
For surgeries in critical locations, ophthalmologic surgeries, and neurosurgeries, when the platelet count is lower than 100,000/mm^3^	IIa	C

**Table:** Recommendations for Perioperative Use of Anticoagulants in Patients with Thrombophilia (Acquired or Hereditary)

Recommendation	Class of recommendation	Level of evidence
In asymptomatic patients with hereditary thrombophilia and persistently positive antiphospholipid tests, antithrombotic prophylaxis in the postoperative period is recommended	I	C
In patients with hereditary thrombophilia or antiphospholipid syndrome undergoing anticoagulant treatment, "bridge" treatment in the perioperative period is recommended	I	C

**Table:** 

Recommendation for Patients with Von Willebrand Disease	Class of recommendation	Level of evidence
All surgical procedures should be based on laboratory measurements of factor VIII activity (FVIII:C) and the activity of the ristocetin cofactor (vWF:RCo) after administration of DDAVP (desmopressin) and/or infusion of concentrate with factor von Willebrand	I	B
During the intraoperative period, FVIII:C and vWF:RCo concentrations should be maintained at 100 IU/dL by infusing the vWF-containing concentrate or in responsive patients by administration of DDAVP	I	B
Whenever possible, surgical procedures should be performed in hospital with medical staff, including hematologist and surgeon, experienced in the treatment of hemorrhagic diseases and with specialized laboratory support	IIa	C
In the postoperative period, FVIII:C concentrations should be 150-250 IU/mL or lower and vWF:RCo equal to or lower than 200 IU/dL to reduce thrombotic risk	IIa	C
Pharmacological antithrombotic prophylaxis should be performed in the postoperative period	IIa	C

**Table:** 

Recommendation for patients with PH	Class of recommendation	Level of evidence
Perioperative evaluation of patients with PH should be performed by a multidisciplinary team	I	C
Evaluation should be based on the clinical data obtained using chest X-ray, pulmonary function test, ECG, echocardiogram, and measurement of biomarkers, such as BNP	I	C
Patients with chronic PTE should maintain anticoagulant therapy throughout the perioperative period	I	C
Specific medications for PH control should be maintained throughout the perioperative period	I	C
Monitoring with invasive blood pressure and pulmonary artery catheter may be used	I	C
Preferentially use vasoactive drugs that do not interfere with PVR	I	C
Right cardiac catheterization can be indicated in the preoperative period of noncardiac surgeries, depending on the clinical condition and the surgical procedure	IIa	C
Inhaled nitric oxide can be used in the perioperative period to control PVR	IIa	C
Patients with PH should preferably perform the surgery in medical centers specialized in this area	IIa	C

**Table:** Recommendations for the Use of Perioperative Steroids

Recommendation	Class of recommendation	Level of evidence
Patients with asthma	IIa	C
Patients with COPD or interstitial lung diseases	IIb	C

**Table:** IV. Recommendations IV. A. Cessation of Smoking in the Preoperative Period

Recommendation	Class of recommendation	Level of evidence
Patients undergoing preoperative evaluation should be encouraged to stop smoking regardless of the time left until the surgery	I	A
Therapeutic intervention should always include the cognitive-behavioral approach associated or not with pharmacological treatment	I	A
Cessation of smoking in this subpopulation reduces surgical and clinical complications	I	A
Any first-line pharmacological option (NRT, bupropion and varenicline), alone or in combination (transdermal nicotine associated with nicotine gum or tablet or bupropion associated with nicotine transdermally, in gum or tablet), may be used in this population, considering individual contraindications. However, there is more evidence supporting NRT	IIa	B

**Table:** IV. B. Smoking Cessation in Hospitalized Patients

Recommendation	Class of recommendation	Level of evidence
Hospitalized patients should be actively approached for antecedent and smoking status	I	C
Smokers should be asked about their intention to quit smoking and about nicotine withdrawal symptoms	I	C
NRT should be initiated in hospitalized smokers experiencing withdrawal symptoms	I	C
Patients treated during hospitalization should be followed for at least one month after discharge to remain abstinent	I	B
NRT is safe and effective in individuals with cardiac disease, even high-risk individuals, including stable coronary disease	IIa	A
Treatments with individualized doses to achieve better control of withdrawal symptoms are safe and well tolerated, but there is no solid evidence that they offer higher success rates in the long term	IIa	B
Prescribe NRT for patients with a history of recent high risk acute coronary syndrome (less than six weeks) and patients with complex ventricular arrhythmias	IIb	C

**Chart 15:** Time for suspension of oral drugs for diabetes control

Class	Drugs	Time for suspension before surgery
Biguanides	metformin	24 to 48 hours
1st G sulfonylureas	chlopropramide	48 to 72 hours
2nd G sulfonylureas	glicazide, glibenclamide, glipizide, glimepiride	on the day of surgery
Thiazolidinediones	pioglitazone	on the day of surgery
Acarbose	acarbose	24 hours
Glinides	repaglinide, nateglinide	on the day of surgery
DPP4 inhibitors	sitagliptin, saxagliptin, vildagliptin, liragliptin, alogliptin	can be maintained even in fasting
GLP1 agonists*	exenatide, liraglutide, lixizenatide	on the day of surgery
SLGT2 inhibitors**	dapagliflozin, canagliflozin, empagliflozin	on the day of surgery

* Slow down gastric emptying;

** Risk of perioperative euglycemic ketoacidosis. G: generation; DPP4: dipeptidyl peptidase 4; GLP1: glucagon like peptide; SLGT2: sodium glucose transporter type 2.
