# Erratum

**DOI:** 10.5935/abc.20180213

**Published:** 2018-10

September issue of 2017, vol. 109 (3), Suppl. 1, pages 1-104

In “3rd Guideline for Perioperative Cardiovascular Evaluation of the Brazilian Society of Cardiology”, consider Marcondes-Braga FG as the correct form for the name of the author Fabiana Goulart Marcondes Braga.

